# Mass balance and long-term soil accumulation of trace elements in arable crop systems amended with urban composts or cattle manure during 17 years

**DOI:** 10.1007/s11356-019-07166-8

**Published:** 2019-12-17

**Authors:** Aurélia Marcelline Michaud, Philippe Cambier, Valérie Sappin-Didier, Valentin Deltreil, Vincent Mercier, Jean-Noël Rampon, Sabine Houot

**Affiliations:** 1 UMR ECOSYS, INRA, AgroParisTech, Université Paris Saclay, 78850 Thiverval-Grignon, France; 2 UMR SAS, INRA, AgroCampusOuest, 65 rue de Saint Brieuc, 35042 Rennes, France; 3 UMR ISPA, INRA, 33883 Villenave d’Ornon, France; 4 Veolia recherche & innovation, Chemin de la digue, 78603 Maisons-Laffitte, France

**Keywords:** Field experiment, Heavy metals, Organic waste products, PNEC, Soil quality, Soil-plant transfer

## Abstract

Organic waste products (OWP) application to crop lands makes possible nutrients recycling. However, it can result in long-term accumulation of trace elements (TE) in soils. The present study aimed at (i) assessing the impact of regular applications of urban composts and manure on the TE contents of topsoils and crops in a long-term field experiment, (ii) comparing the TE mass balances with the stock variations of TE in soils, and (iii) proposing a prospective evaluation of this practice, based on estimated soil safe threshold values and simulations of soil TE accumulation for 100 years. In the long-term field experiment, physico-chemical properties and TE contents (Cd, Cr, Cu, Hg, Ni, Pb and Zn) have been measured in OWP, soils, plants and leaching waters for the period 1998–2015, and used for mass balance calculations and long-term simulations of TE accumulations. The composts of green wastes and sludge (GWS) and of municipal solid waste (MSW) were the OWP with the largest TE contents, while the farmyard manure tended to have the lowest. Repeated application of OWP led to significant accumulation of Zn and Cu in the topsoil layer (not for Cr, Cd, Hg, Ni, Pb), especially with GWS, without overpassing calculated protective threshold values. No effect of repeated application of OWP has been observed on TE contents in grains (wheat, maize, barley). The positive mass balance has been dominated by the input flux of TE through OWP and resulted in the observed increases of soil stocks for Cu and Zn. Prospective simulation of soil content evolution until 2100 showed that soil content reached 0.4 mg Cd kg^−1^ soil (GWS, MSW), 38 mg Cu kg^−1^ soil (GWS) and 109 mg Zn kg^−1^ soil (GWS), which remained lower than protective threshold values.

**Electronic supplementary material:**

The online version of this article (10.1007/s11356-019-07166-8) contains supplementary material, which is available to authorized users.

## Introduction

The biggest challenge that agriculture faces is to produce food enough in quantity and quality to feed increasing population, in the complex context of climate change. Agriculture sustainability needs to consider soil fertility, facing soil threats such as decreasing soil organic carbon (Richard et al. 2019). After industrial revolution, nutrient recycling has been neglected due to the worldwide increased use of mineral fertilizers (Richard et al. 2019), although manure application on soils has traditionally contributed to the recycling of nutrients they contain. Recycling of organic waste products (OWP) in agriculture contributes to circular economy through the recycling of nutrients and organic matter and represents a valuable alternative to their landfilling or incineration (Houot et al. 2014; Noirot-Cosson et al. 2016; Alvarenga et al. 2017). The large variety of waste sources and treatment processes result in large diversity of OWP with various properties (Houot et al. 2014; Sharma et al. 2017). The use of OWP from urban and industrial origin may replace the traditional use of manure in areas where animal breeding is lacking and may be used as a substitute to chemical fertilizers (Noirot-Cosson et al. 2016; Sharma et al. 2017).

However, the recycling of OWP may represent potential risks to the environment and alimentation, due to their contents in contaminants, such as organic contaminants, biological pathogens and trace elements (Gigliotti et al. 1996; Weber et al. 2007; Alvarenga et al. 2017; Bourdat-Deschamps et al. 2017). The repeated use of OWP as organic fertilizer or soil amendments can thus result in long-term accumulation of contaminants in the soil (Delschen 1999) which can cause problems if certain concentration levels are exceeded (Moolenaar et al. 1997a). Prevention of trace elements (TE) accumulation is needed for sustainable agricultural productions (Moolenaar et al. 1997a). Among TE, the most concern is for non-essentials and toxic ones (e.g. As, Cd, Hg, Pb), while trace elements also considered as micronutrients (e.g. B, Cu, Zn) can be phytotoxic at concentrations that pose little risks to human and animal health (Lopez-Rayo et al. 2016). Nevertheless, Cu and Zn are micronutrients of most concern. Indeed, studies showed that TE accumulation including Cu and Zn was associated with decreases in diversity and activity of soil microorganisms, suggesting that the soil microorganisms are more sensitive to TE than higher plants, which can impair biogeochemical processes such as specific pathways of nutrient cycling and organic matter decomposition (McGrath et al. 1995; Charlton et al. 2016). Even with quality thresholds regulating the application of OWP onto soil, the regular application of OWP may increase TE concentrations in soils (Kirchmann et al. 2017) to levels toxic to soil microorganisms and affect biological processes (Moolenaar and Beltrami 1998). Recently, tools and framework have been developed to estimate thresholds values and provide a risk assessment based on eco-toxicological studies (Heemsbergen et al. 2009; Smolders et al. 2009): the predicted-no-effect concentrations (PNECs) calculator and the “threshold calculator for metals in soil” (Oorts et al. 2018). These methods not only consider total TE concentration in soils which is a poor indicator of TE toxicity risks, but also soil physico-chemical properties known to influence the speciation of TE and alter their toxicity and the specific sensitivity of various species to TE toxicity. Risks associated to relatively low TE concentrations found in soils after repeated applications of OWP have seldom been studied, except for example by Heemsgergen et al. (2009) about the guidelines for amended soils with biosolids.

The balance of nutrients, including elemental major inputs and outputs within the field boundary, is an important approach to evaluate the sustainability of an agrosystem and the effects of long-term activities on the soil (Yu et al. 2011). This balance approach has proven to be useful in soil fertility studies to demonstrate depletion of nutrients from soils. It has also been used in cases of soil pollution to demonstrate accumulation of TE in agricultural soils (Moolenaar and Lexmond 1998). Further use of the mass balance is to calculate the long-term change of TE content in the upper layer of soil. As presented by Moolenaar and Lexmond (1998), the change in total TE content in the topsoil layer results from the balance between inputs and outputs. Recently this mass balance approach has been used at the regional and national scale to simulate the evolution of TE concentration in the topsoil (Six and Smolders 2014; Sterckeman et al. 2018). Analysis at national level cannot consider relevant processes on smaller scales because of the average values and estimations used at larger scale. Belon et al. (2012) recognized that their approach at the department scale was not sufficient to help farmers in making decisions. They also pointed out that at the plot scale the relative importance of the diverse sources of TE may differ from the national and department ones. Furthermore, a balance assessment at the larger scale may lead to wrong conclusions locally. To that respect, it is important to complete and validate national-scale studies by field-scale studies considering precise agronomical practices and actual data for the agronomic compartments (i.e. fertilizer and amendment inputs, atmospheric depositions, plant offtake, leaching) (Moolenaar et al. 1997b; Moolenaar and Lexmond 1998). Nevertheless, the assessment of TE balance is often impossible in many situations due to the lack of outputs data with crop offtakes and leaching (Moolenaar et al. 1997a; Yu et al. 2011). In addition, models and simulations are not a substitute for real data and data obtained from long-term studies are essential for setting legislative limits for concentrations of TE in agricultural soils (Uprety et al. 2009).

Most field experimentations devoted to the evaluation of OWP application effects have been carried out in short-term studies (Douglas et al. 2003; Warman and Termeer 2005), often focusing on nitrogen fertilization (Bell and Leclerc 2015). As underlined by Lopez-Rayo et al. (2016), long-term experiments can give realistic overview of cumulative effects on soil properties and plant quality when applying OWP. Among short- and long-term field experiments devoted to the study of OWP application effects on TE dynamic, sewage sludge and manures were mainly studied (McGrath et al. 1995; Chaudri et al. 2000, 2007; Uprety et al. 2009; Hamner and Kirchman 2015; Charlton et al. 2016). Few short-term experiments were carried out with municipal solid waste or sewage sludge composts (Giusquiani et al. 1994; Businelli et al. 1996; Gigliotti et al. 1996; Weber et al. 2007). Much less studies considered various urban OWP including composts in long-term experiments: the work of Delschen (1999), Riber et al. (2014) and associated studies carried out in the CRUCIAL field experiment, and Houot et al. (2002) and related studies on the “QualiAgro” field experiment. To our knowledge, none of these studies provided actual TE mass balance at the field scale with in situ measured data (i.e. inputs, outputs, and total balance) after repeated application of various urban OWP, and tested the mass balance model to simulate long-term accumulation of TE in the topsoil layer.

Thus, the aim of the present study was to (i) assess the impact of regular applications of urban composts and manure on the TE contents of topsoils and crops in the “QualiAgro” field experiment, (ii) compare mass balances with the stock variations of TE in the topsoil layer, and (iii) propose a prospective evaluation based on soil safe threshold values and simulation of TE accumulation in the topsoil layer for 100 years of agricultural repeated spreading of various OWP.

## Materiel and methods

### QualiAgro field experiment

The QualiAgro field experiment was set up in 1998 to investigate the long-term effects of repeated applications of urban composts on soil fertility and potential contamination with inorganic and organic contaminants (Houot et al. 2002; Cambier et al. 2019). It is located within the Plateau des Alluets (Yvelines, France), on a silt loam Luvisol, according to IUSS-FAO classification and USDA texture triangle, developed on aeolian loess. The soil of about more than 1.2 m deep is developed on carbonated loess deposits. The upper horizons include a plough layer (i.e. topsoil) and a plough pan layer or ancient ploughed layer between 28 and 35 cm. Horizons of clay accumulation appear around 50–60 cm depth. All these horizons are decarbonated, the carbonated silt loess appearing below 1.4–1.6 m depth. The initial physico-chemical characteristics of the topsoil layer are presented in Table 1. From 1999 to 2015, the 6-ha field experiment has been cropped with a biennial rotation of maize and winter wheat, except in 2007, when spring barley was inserted because of infestation by chrysomela in the region (Appendix A in Electronic supplementary material). After grain harvest, the maize residues are incorporated into the topsoil layer but the wheat crop residues are exported. The climate is oceanic, with mean annual precipitation of 572 mm and mean annual temperature of 11 °C. The experiment could be considered as representative of cultivated soils of the central Paris Basin and of extended agricultural areas of NW Europe.

**Table 1:** Chemical properties and trace element contents measured in the topsoil layer (0–25 cm, sieved < 2 mm) at the beginning of the experiment in 1998, with the following: texture (g kg^−1^ DM; DM for dry matter), pH, cationic exchange capacity (CEC; cmol^+^ kg^−1^ DM), content of organic carbon (Org. C), total nitrogen (Total N) and Olsen extractable phosphorus (Olsen P_2_O_5_) (g kg^−1^ DM), trace element content for Cd, Cr, Cu, Hg, Ni, Pb and Zn (mg kg^−1^ DM); mean values (standard deviations for all plots), compared to the mean region contents

Clay	Silt	Sand	pH	CEC	Org. C	Total N	Olsen P_2_O_5_
	g kg^−1^ DM			cmol^+^ kg^−1^ DM	g kg^−1^ DM		
145.8 (7.5)	785.0 (5.4)	69.2 (4.5)	7.06 (0.19)	9.7 (0.8)	10.6 (0.6)	1.12 (0.05)	0.09 (0.01)
	Cd	Cr	Cu	Hg	Ni	Pb	Zn
				mg kg^-1^ DM			
	0.24 (0.01)	45.6 (3.1)	12.0 (1.0)	0.10 (0.02)	14.8 (0.7)	25.7 (4.1)	51.6 (3.5)
Referential^a^	0.25 (0.16)	41 (12)	12 (11)	0.07 (0.16)	18 (6)	23 (12)	52 (20)

^a^Topsoil layers of agricultural soils of “Ile-de-France” (central Paris Basin): medians (standard deviations), *n* between 2000 and 4000, according to Baize et al. (2007)

**Fig. 1:**
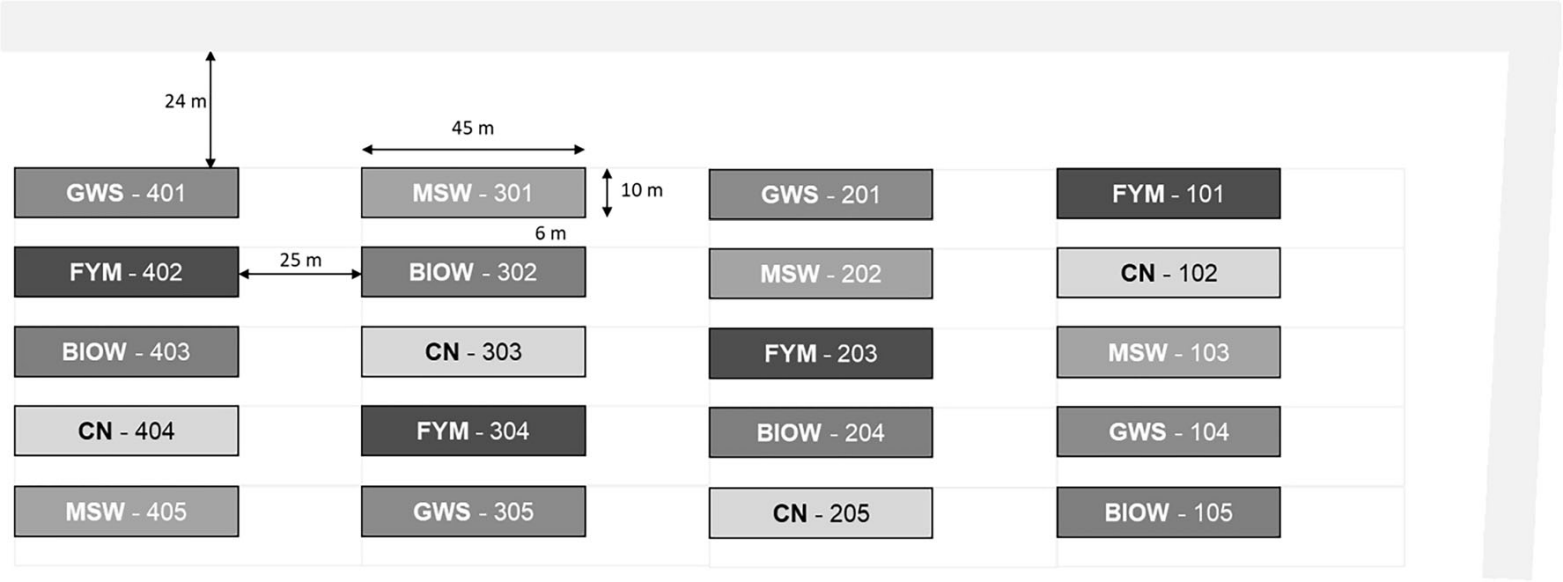
Experimental plan of the QualiAgro device with the following: GWS for co-compost of sewage sludge with green waste and wood chips, BIOW for co-compost of biowaste and green waste, MSW for compost of organic fraction of municipal solid waste, FYM for farmyard manure of dairy cows on straw, CN for control without amendments

Figure 1 presents the experimental plan of the QualiAgro field experiment, with the distribution of the 20 plots of 45 × 10 m^2^ in 4 blocks of replicates of the following 5 treatments randomly distributed within each block:

(1) Co-compost of sewage sludge with green waste and wood chips (GWS),

(2) Co-compost of the home-sorted fermentable fraction of municipal solid waste and green waste, also called biowaste compost (BIOW),

(3) Compost of residual municipal solid waste after sorting of recyclable packaging and papers (MSW),

(4) Farmyard manure from a dairy farm, used as a reference organic amendment (FYM),

(5) No organic amendment (control or CN).

For the period 1998–2013, additional mineral nitrogen fertilization was applied in the form of “Solution 39” (urea + ammonitrate), at doses between 100 and 170 kg N ha^−1^ on wheat and between 0 and 80 kg N ha^−1^ on maize, depending on mineral N stocks measured in plots down to 90 cm, at the end of winter before fertilization (Noirot-Cosson et al. 2016). No mineral fertilization of potassium and phosphorus was applied during the period 1998–2013. All treatments and plots received similar pesticide treatments and cropping practices. In 2014 and 2015, according to the new management strategy of the experiment which has been converted into organic farming practices, the additional fertilization was applied with the organic commercial product “Axe N+P”.

Each OWP application was made on the wheat stubbles every 2 years in September, at doses equivalent to 4 t organic C ha^−1^. This corresponded to average doses of 18.3 (MSW), 26.2 (GWS), 27.7 (BIOW) and 34.6 (FYM) tons of fresh matter per hectare, i.e. about 1.5- to 3-fold larger than usual practices (average recommended amounts: 20–30 t ha^−1^ per 3 years for farmyard manure and 10–20 t ha^−1^ per 3 years for composts). More precise characteristics of each amendment are given in “Characteristics of applied organic waste products” section.

### Sampling and analyses

Soils were sampled before each OWP spreading. Each plot was sampled in the topsoil layer between 0 and 25 cm depth (10 spadesful further pooled). After air drying, the fine earth (< 2 mm) was prepared according to the standard ISO 11464 and kept for further analyses. In 1998, 2010 and 2015, soils samples were also taken from the layer located below the topsoil layer (25–35 cm) for physico-chemical analyses.

In 2013–2014 and 2014–2015, soil solutions were sampled for further analyses at the depth of 45 cm during drainage seasons with lysimeters with fibreglass wicks (45 cm deep) (Cambier et al. 2014; Chabauty et al. 2016).

Between 1998 and 2013, a total of 9 OWP spreading have been carried out (Appendix A in Electronic supplementary material). All OWP were sampled during application for analysis.

Just before the mechanical harvest, plants were sampled (i.e. for each plot harvested on 10 subplots of 0.5 m triple row for wheat and 5 subplots of 2.5 m double row for maize, pooled to obtain one composite sample per plot) and grains and aerial crop residues separated to measure crop yields and biomasses of residues.

The soil bulk density was measured in the top soil layers simultaneously to soil sampling before OWP spreading, in each plot in 1998, 2004, 2009 and 2013, by using vertical stainless cylinders (French standard X31-501 or ISO 11272).

Soils, OWP and leachates were analysed by the central soil testing laboratory of INRA (INRA LAS, Arras) according to standardized French (AFNOR) or international procedures (ISO). Soil and OWP pH were measured in water using a 1:5 soil/solution ratio (NF EN 15933). Soil organic carbon was determined by dry combustion (corrected from the carbonates) (NF ISO 10694); effective cation exchange capacity (CEC) was determined by cobaltihexamine chloride extraction (NF ISO 23470). In OWP and soil samples, total trace element concentrations of Cd, Cr, Cu, Ni, Pb and Zn were determined on 250 μm crushed samples by HF–HClO_4_ extraction (NF X31-147). Trace elements analyses were performed by inductively coupled plasma atomic emission spectroscopy (ICP-AES) for Cr, Cu, Ni and Zn or inductively coupled plasma mass spectrometry (ICP-MS) for Cd and Pb. Total Hg concentration was estimated by dry combustion on 250 μm crushed samples. Other chemical properties were determined as follows: in soils, N total content by NF ISO 13878, P extracted by the Olsen method NF ISO 11263; in OWP, organic N was calculated from total N (dry combustion) and mineral N, organic C by dry combustion, P by HF–HClO_4_ extraction NF X31-147, CaCO_3_ content by NF ISO 10693. TE concentrations in leachates were analysed according to standard methods by ICP-MS.

All plant analyses were carried out in the central plant testing laboratory of INRA (INRA USRAVE, France) according to standardized French (AFNOR) or international procedures (ISO). Grains and crop residues were oven dried (*T*° < 40 °C) then ground under 0.5 mm. Plant samples were digested in a microwave oven with a sequential procedure including digestion with concentrated nitric acid, H_2_O_2_ and HF–HClO_4_. Trace elements analyses were performed by ICP-AES for Cu, Zn or ICP-MS for Cd, Cr, Ni and Pb. Total Hg concentration was analysed by dry combustion on < 0.5 mm crushed samples.

### Calculations

#### Soil stocks of trace elements in the topsoil layer

Organic waste products have been incorporated into the topsoil layer. The stock *Q*_top,*i*,*n*_ (kg ha^−1^) of any element contained in the topsoil layer is given by:

1 $$ {Q}_{top,i,n}={\left[ TE\right]}_{top,i,n}\times {W}_{top,i,n}\times 0.001={\left[ TE\right]}_{top,i,n}\times \kern0.33em h\kern0.33em \times {d}_{top,i,n}\times 10 $$

Where,

top refers to the topsoil layer, *i* to the treatment and *n* to the year.

*W*_top,*i*,*n*_ is the soil mass (t ha^−1^) in the topsoil layer (Appendix B in Electronic supplementary material).

[TE]_top,*i*,*n*_ is the trace element content (mg kg^−1^ or g t^−1^) (Appendix C in Electronic supplementary material).

*h* is the height of the soil layer.

*d*_top,*i*,*n*_ is the soil density.

Since the beginning of the experiment, the soil bulk density was measured in 1998, 2004, 2009 and 2013, and calculated using an adjusted polynomial equation for other sampling dates (Appendix B in Electronic supplementary material). It increased in the topsoil layers, more in the control treatment than in the organic treatments. This led to an increase of the soil mass since 1998, especially in the control treatment. Nevertheless, soil stocks must be calculated on a fixed soil mass basis (Ellert and Bettany 1995; Peltre et al. 2012). As the soil bulk density of the control treatment in 2015 was the largest one of all, its mass was considered as the basis for further calculations of corrected soil stock. The mass *W*_top,CN,2015_ of 3800 t ha^−1^ was thus used, corresponding to the bulk soil density of 1.52 and a soil depth of 0.25 cm.

According to Peltre et al. (2012), we calculated corrected stocks in the topsoil layer of year *n* (Qc_top,*i*,*n*_) by adding a stock of an element present in the sub-layer progressively incorporated between 1998 and year *n* (*Q*_sub,*i*,*n*_).

The stock of TE from the sub-layer to be added to the topsoil layer stock was calculated with:

2 $$ {Q}_{sub,i,n}=\left({W}_{top, CN,2015}-{W}_{top,i,n}\right)\kern0.33em \times \kern0.33em {\left[ TE\right]}_{sub,i,n} $$

Where,

sub refers to the sub-layer (25–35 cm), *i* to the treatment and *n* to the year.

*W*_top_ is the soil mass in the topsoil layer.

[TE]_sub,*i*,*n*_ is the trace element content (mg kg^−1^) in the sub-layer (Appendix D in Electronic supplementary material).

The TE contents of sub-layers under the topsoil layer have been measured in 1998 in a few places, and in 2010 and 2015 for each plot (Table D.1. in Appendix D, Electronic supplementary material). Considering the lack of data for the beginning of the experiment in 1998, [TE]_sub,CN,2015_ (with sub for sub-layer, CN for the control treatment and 2015 for the considered year) was considered as the reference for [TE]_sub,*i*,1998_ at the beginning of the experiment (i.e. 1998) in all treatments, while the [TE]_sub,*i*,2015_ measured in the OWP treatments in 2015 was considered as the final contents in 2015. Then, we used linear functions to interpolate [TE]_sub,*i*,*n*_ between 1998 and 2015, [TE]_sub,CN,*n*_ being assumed constant (detailed calculations and equations in Appendix D):

3 $$ {\left[ TE\right]}_{sub,i,n}=a\kern0.33em \times \kern0.33em \left(n-1998\right)+{\left[ TE\right]}_{sub, CN,2015} $$

The corrected stock of TE in the topsoil layer (Qc_top_), corresponding to the constant mass of soil of 3800 t ha^−1^, was calculated with (detailed calculations in Appendix E):

4 $$ Q{c}_{top,i,n}={Q}_{top,i,n}+{Q}_{sub,i,n} $$

Where, *Q*_top,*i*,*n*_ (kg ha^−1^) is the stock of TE contained in the topsoil layer and *Q*_sub,*i*,*n*_ is the stock of TE from the sub-layer to be added to the stock of the topsoil layer.

#### Mass balance calculations

Input and output fluxes of TE were calculated at the field scale (kg ha^−1^): inputs through OWP applications and atmospheric deposits; outputs through leaching water and crop uptakes. The input fluxes due to organic fertilizers in 2014 and 2015 represented less than 0.7% of the total input of TE with OWP application so that it has been neglected in the following calculations (data in Appendix G).

A balance of trace elements in soil relates the rates of accumulation input − output (Moolenaar et al. 1997b),

5 $$ Mass\kern0.33em balance=\sum \kern0.33em Q\kern0.33em input\kern0.33em -\kern0.33em \sum \kern0.33em Q\kern0.33em output $$

Where,

6 $$ Q\kern0.33em input=\sum \kern0.33em {Q}_{atm,i,n}+\kern0.33em \sum \kern0.33em {Q}_{OWP,i,n} $$

7 $$ Q\kern0.33em ouput=\kern0.33em \sum \kern0.33em {Q}_{lea,i,n}+\sum \kern0.33em {Q}_{crop,i,n} $$

*Q*_atm,*i*,*n*_ (kg ha^−1^) is the input flux related to atmospheric deposits of TE, calculated from Azimi et al. (2004) with mean TE atmospheric deposition rate obtained in the Parisian region (g ha^−1^ year^−1^).

*Q*_OWP,*i*,*n*_ (kg ha^−1^) is the TE input flux related to OWP application, calculated for each application from measured applied amount of OWP (*q*_OWP,*i*,*n*_ in tons DM ha^−1^) and TE measured in the corresponding OWP ([TE]_OWP,*i*,*n*_ in mg kg^−1^ DM) (*Q*_OWP,*i*,*n*_ = [TE]_OWP,*i*,*n*_ × *q*_OWP,*i*,*n*_) (detailed OWP characteristics used for *Q*_OWP_ calculations in Appendix F).

*Q*_lea,*i*,*n*_ (kg ha^−1^) is the output flux of TE associated to leached waters collected at 45 cm depth, based on the mean annual flux measured during 2 drainage periods (2013–2015) (detailed leaching waters characteristics used for *Q*_lea,*i*,*n*_ calculations in Appendix H).

*Q*_crop,*i*,*n*_ (kg ha^−1^) is the output flux of TE related to crop uptake, calculated each year from the yields measured for each crop (*q*_crop,*i*,*n*_, ton DM ha^−1^) for exported grains and wheat crop residues and TE concentration measured in corresponding plant samples (mg kg^−1^ DM) (*Q*_crop,*i*,*n*_ = [TE]_crop,*i*,*n*_ × *q*_crop,*i*,*n*_ × 0.001) (detailed crop characteristics used for *Q*_crop,*i*,*n*_ calculations in Appendix I). For the [TE]_crop,*i*,*n*_ lower than the quantification limits, we used raw values when they were given by the laboratory or quantification limit divided by 2 were considered to estimate *Q*_crop,*i*,*n*_.

#### Simulation of soil trace element content

The increase in TE content in the topsoil layer is the result of the net balance between input and output flows divided by the plough layer weight (Moolenaar et al. 1997b).

We adapted the equations of Six and Smolders (2014) and Sterckeman et al. (2018) to calculate annually the TE content in the topsoil layer and simulate long-term evolution of trace element contents in the topsoil layer, i.e. [TE]_top,*i*,*n*_:

8 $$ {\left[ TE\right]}_{top,i,n}={\left[ TE\right]}_{top,i,n-1}+\frac{Qm\kern0.33em input- Qm\kern0.33em output}{Wtop} $$

Where,

The soil TE content over the year *n* ([TE]_top,*i*,*n*_, in mg TE kg^−1^ soil) is the result of that in year *n* − 1 ([TE]_top,*i*, *n* − 1_, in mg TE kg^−1^ soil) and of the balance between the mean input (*Q*_*m*,*i*_ input, g ha^−1^) and the mean output (*Q*_*m*,i_ output, g ha^−1^) of TE in the considered topsoil layer, whose weight is *W*_top_ (3 800 tonnes ha^−1^).

*Q*_*m*,*i*_ input and *Q*_*m*,*i*_ output correspond to average annual quantity of TE calculated from data presented in “Mass balance calculations” section (detailed mean annual fluxes data in Appendix J):

9 $$ {Q}_{m,i}\kern0.33em input=\frac{\sum Qatm+\sum QOWP}{Number\kern0.33em of\kern0.33em years} $$

10 $$ {Q}_{m,i}\kern0.33em output=\frac{\sum Qlea+\sum Qcrop}{Number\kern0.33em of\kern0.33em years}. $$

#### Threshold contents for trace elements in soil

Ecological threshold contents in soils for trophic levels (i.e. plants, invertebrates and microbial processes) were estimated with the threshold calculator for metal in soil V2.0® (Arche Consulting, https://www.arche-consulting.be/) (Oorts et al. 2018). The calculator has been developed using the studies of Smolders et al. (2009) and Checkai et al. (2014) based on datasets derived from scientific literature, research projects and European REACH dossiers (Registration, Evaluation, Authorisation and Restriction of Chemicals EC No 1907/2006).

The 5^th^ percentile of a chronic toxicity distribution has been chosen under the EU REACH Regulation as a concentration that is protective for most species in a community (namely 95%). Therefore, the PNEC value (i.e. predicted no effect concentration) is based on the 5% probability level (i.e. 5% Hazardous Concentration or HC_5_) of the reliable EC10 (i.e. Effective Concentration for which 10% of the community is affected by the concentration of the trace element) divided by an assessment factor between 1 and 5 (ECHA 2008; OECD 2016). Finally, the HC_5_ of EC 10 values, without additional assessment factor, has been chosen as a protective threshold value for Cd, Cu, Ni, Pb and Zn for the trophic levels plants, invertebrates and microorganisms. No sufficient soil eco-toxicity data are available for Hg and Cr to use a species sensitivity distribution approach, nor for implementation of bioavailability corrections for the derivation of ecological threshold concentrations of these elements.

The soil properties required as input parameters for bioavailability corrections were measured by the adequate methods mentioned by Oorts (2018) for organic C, clay content and effective cation exchange capacity. Only pH 0.01 M CaCl_2_ was estimated from the measured pH_H2O_ by using the proposed equation pH_CaCl2_ = − 0.54 + 1.00 × pH_H2O_.

### Statistic treatments

Parametric or non-parametric tests were applied after testing normality of residues and equality of variances. Tests were performed as follows using StaBoxAgri® (2009) and XLSTAT® (V 2018.2, Addinsoft): ANOVA parametric and Kruskal-Wallis non-parametric tests were performed to identify the treatment effect per year; Newman-Keuls parametric or Bilateral Dunn non-parametric tests were performed to identify significant differences between treatments per year; Friedman’s non-parametric test was performed to evaluate significant effect of years with Bilateral Nemenyi non-parametric method used for multiple comparisons for paired samples.

## Results and discussion

### Characteristics of applied organic waste products

The 4 OWP applied in the QualiAgro field experiment differed in their physico-chemical characteristics (Table 2) with average characteristics in agreement with previous studies (Sen Tran et al., 1996; Houot et al. 2014). As already stressed by Obriot et al. (2016), the largest pH was found in FYM and BIOW, largest content in carbonates in BIOW, largest organic carbon content in FYM, largest organic nitrogen content in GWS and FYM, and largest content in phosphorus and potassium were found in GWS and FYM, respectively.

**Table 2:** Physico-chemical properties of the organic waste products studied in the QualiAgro field experiment, as compared to the French regulation level NFU 44 051/095 for each trace element, with the following: dry matter (DM) expressed as percentage of fresh matter (FM), pH, content of CaCO_3_, organic carbon (Org. C) and nitrogen (Org. N), total content of phosphorus (P_2_O_5_) and potassium (K) (g kg^−1^ DM), trace element content for Cd, Cr, Cu, Hg, Ni, Pb and Zn (mg kg^−1^ DM); average values for the period 1998–2013 ± standard deviations (*n* = 9); co-compost of sewage sludge and green waste (GWS), biowaste compost (BIOW), compost of residual municipal solid waste (MSW), farmyard manure (FYM)

OWP	DM	pH	CaCO_3_	Org. C	Org. N	P_2_O_5_	K	Cd	Cr	Cu	Hg	Ni	Pb	Zn
	% FM		g kg^−1^ DM					mg kg^−1^ DM						
GWS	63 ± 9	7.5 ± 0.6	28 ± 12	265 ± 45	21 ± 2	29 ± 8	15 ± 5	1.1 ± 0.7	40 ± 7	174 ± 43	0.8 ± 0.3	27 ± 7	62 ± 11	406 ± 89
BIOW	70 ± 9	8.1 ± 0.5	90 ± 62	211 ± 47	17 ± 4	11 ± 4	21 ± 3	0.7 ± 0.7	38 ± 15	62 ± 26	0.2 ± 0.1	25 ± 24	87 ± 56	235 ± 100
MSW	68 ± 13	7.5 ± 0.5	69 ± 23	310 ± 45	17 ± 2	8 ± 2	10 ± 3	1.4 ± 0.7	86 ± 57	143 ± 87	0.8 ± 0.8	30 ± 18	140 ± 87	388 ± 206
FYM	40 ± 10	9.1 ± 0.3	47 ± 17	324 ± 68	21 ± 3	13 ± 3	35 ± 3	1.1 ± 1.1	34 ± 24	95 ± 70	0.1 ± 0.0	12 ± 9	115 ± 127	342 ± 189
NFU 44 051/095								3	120	300	2	60	180	600

**Fig. 2:**
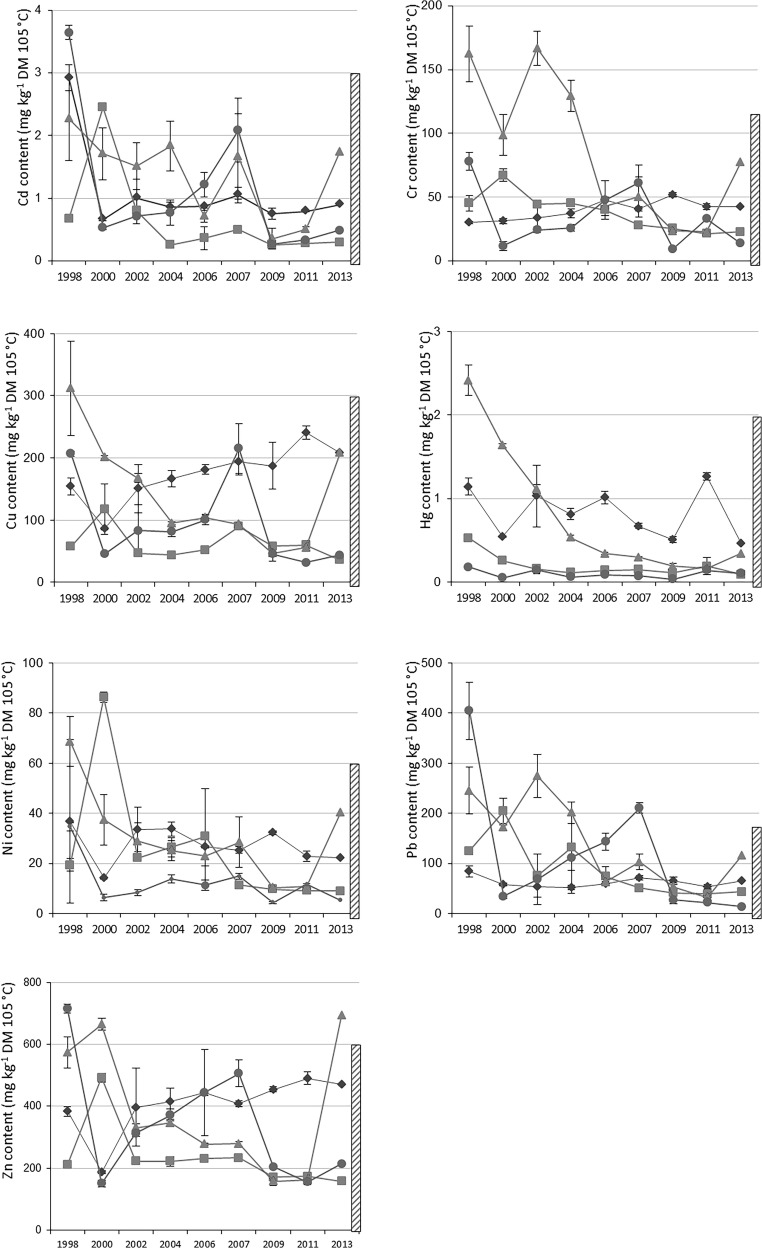
Evolution of trace element contents in applied organic waste products (OWP) over the period 1998–2015 for Cd, Cr, Cu, Hg, Ni, Pb and Zn (mg kg^−1^ DM; DM for dry matter). Diamonds stand for co-compost of sewage sludge and green waste (GWS), squares stand for biowaste compost (BIOW), triangles stand for compost of residual municipal solid waste (MSW) and circles stand for farmyard manure (FYM). The hatched rectangle corresponds to the French regulation level for each trace element. The error bars are standard deviations

Average contents in total TE in the OWP are presented in Table 2 and Fig. 2. Over the period 1998–2015, all GWS composts presented TE contents lower than regulatory thresholds defined in NFU 44-095 in application since 2002 for such composts. In 2000, the BIOW composts were above the thresholds for Pb and Ni defined in NFU 44-051 in application since 2006. Surprisingly, the contents in Cd, Pb, Zn were above the thresholds for FYM in 1998. The MSW composts overpassed the limits more often: for Cr and Pb in 1998, 2002, 2004; for Cu, Hg and Ni in 1998; for Zn in 2000 and 2013; but MSW composts conformed to the regulation in 2006, 2007, 2009, 2011, and Cd was always under the regulatory limit.

For most TE, the contents in GWS and MSW composts often appeared above those in BIOW and FYM, although not always significantly (Table 2; statistics in Appendix F in Electronic supplementary material). On average, total contents in TE were larger in the MSW compost for Cd, Cr, Ni and Pb compared to the other OWP, in the GWS compost for Cu and Zn. The TE contents were in agreement with previous studies (McBride and Spiers 2001; Zhang et al. 2012; Alvarenga et al. 2015, 2017).

An overall decrease of contents of most TE was observed with time, in the different OWP except for GWS compost, suggesting improvement of the OWP quality with time (Fig. 2). Therefore, a more meaningful comparison between OWP is given below through the cumulated TE inputs (Table 3).

**Table 3:** Input fluxes by organic waste products (OWP) studied in the QualiAgro field experiment, with the following: the applied quantity expressed as tons of dry matter per hectare (t DM ha^−1^), the input flux for organic carbon (Org. C) and the trace elements (Cd, Cr, Cu, Hg, Ni, Pb, Zn) expressed as kg ha^−1^; average input per spreading, cumulated input over the period 1998–2015, cumulated input relative to 10 years of spreading as compared to the French regulation level NFU 44 051/095 (NFU) for each trace element; co-compost of sewage sludge and green waste (GWS), biowaste compost (BIOW), compost of residual municipal solid waste (MSW), farmyard manure (FYM)

**OWP**	**Input type**	Applied quantity	Org. C	Cd	Cr	Cu	Hg	Ni	Pb	Zn
		t DM ha^−1^	kg ha^−1^							
GWS	Average input	16.5	4357	0.017	0.7	2.8	0.013	0.4	1.0	6.6
BIOW	Average input	19.1	3922	0.014	0.8	1.2	0.004	0.5	1.8	4.7
MSW	Average input	12.2	3745	0.017	1.1	1.8	0.010	0.4	1.7	5.0
FYM	Average input	12.8	4058	0.015	0.5	1.3	0.001	0.2	1.5	4.5
GWS	Cumulated input	149	39213	0.150	5.9	25.6	0.119	4.0	9.1	59.6
BIOW	Cumulated input	172	35302	0.126	6.8	10.9	0.032	4.8	15.9	42.3
MSW	Cumulated input	110	33705	0.154	9.5	16.0	0.089	3.4	15.5	45.3
FYM	Cumulated input	115	36524	0.133	4.1	11.3	0.012	1.5	13.9	40.6
GWS	Relative to 10 years	87	23066	0.088	3.5	15.0	0.070	2.4	5.4	35.1
BIOW	Relative to 10 years	101	20766	0.074	4.0	6.4	0.019	2.8	9.4	24.9
MSW	Relative to 10 years	65	19826	0.091	5.6	9.4	0.052	2.0	9.1	26.7
FYM	Relative to 10 years	68	21485	0.078	2.4	6.6	0.007	0.9	8.2	23.9
NFU	Relative to 10 years			0.15	6.0	10.0	0.10	3.0	9.0	30.0

The TE input for each spreading and their accumulation were calculated and reported to a 10-year period for comparison with the relevant regulatory French limits, i.e. in application since 2002 for NFU 44-095 and since 2006 for NFU 44-051. Despite a total input of Zn and Cu which exceeded the French regulation for GWS, and of Pb with the MSW and BIOW composts, the OWP studied in QualiAgro field experiment were globally in accordance with French national regulations. The input of Pb was about two times less with GSW applications than with other products; the input of Ni was low with FYM; the input of Hg was lower with BIOW and FYM than with GWS and MSW applications. The general observation, with a few exceptions, is that the inputs for each TE were comparable among treatments and within the range of the regulatory maximum. It must be noticed that amounts of applied OWP have been about 1.5- to 3-fold larger than usual practices, which led to maximize risks related to input fluxes of TE. Copper and zinc were added in greater amounts (6.6–15.0 kg ha^−1^ 10 years^−1^ for Cu; 23.9–35.1 kg ha^−1^ 10 years^−1^ for Zn) compared to the other TE. Mercury and cadmium were added in the rates respectively of 0.01–0.07 kg ha^−1^ 10 years^−1^ and 0.07–0.09 kg ha^−1^ 10 years^−1^. Repeated spreading of GWS led to greater amounts of Cd, Cu, Hg and Zn. Spreading of BIOW led to greater amounts of Ni and Pb, and MSW presented the largest input fluxes of Cd and Cr. Spreading of FYM led globally to the lowest inputs of TE.

### Effect of organic waste product applications on soil physico-chemical properties and trace elements contents

The physico-chemical characteristics of the topsoil layer sampled in 1998 before the first OWP application have been summarized in Table 1. Before the first OWP application, the total contents of TE did not significantly differ between the plots selected for each treatment (*P* = 0.05, *n* = 4, test of Newman-Keuls for Cd, Cr, Cu, Hg, Ni and Zn, non-parametric test for Pb). Initial TE contents in the topsoil layer were in the range of those found in similar soils of the Parisian region (Baize et al. 2007), and with median contents found in France (BDETM 2019) (Table 4) with the following sequence: Zn > Cr > Pb > Ni > Cu > Cd > Hg. The initial soil pH was 7.06, and the organic carbon content was in average 10.61 g kg^−1^ of dry soil.

**Table 4:** Chemical properties and total trace element contents measured in 2015 in the topsoil layer (0–25 cm, sieved < 2 mm), compared to the average French and European trace element contents, with the following: mean values ± standard deviation of the four replicates (Abc letters stand for significant difference between treatments); co-compost of sewage sludge and green waste (GWS), biowaste compost (BIOW), compost of residual municipal solid waste (MSW), farmyard manure (FYM), no organic amendment (CN); na for not available

Treat.	pH	Org. C	Total N	Olsen P_2_O_5_	CEC	Cd	Cr	Cu	Hg	Ni	Pb	Zn
		g kg^−1^ DM			cmol^+^ kg^−1^ DM	mg kg^−1^ DM						
GWS	7.0 ± 0.0 (c)	16.9 ± 0.9 (a)	1.7 ± 0.1 (a)	0.20 ± 0.02 (b)	10.7 ± 0.4 (b)	0.25 ± 0.01 (ab)	73.4 ± 10.2 (ab)	19.9 ± 1.1 (a)	0.12 ± 0.02 (a)	15.6 ± 0.2 (a)	27.3 ± 5.5 (a)	68.2 ± 2.2 (b)
BIOW	7.8 ± 0.1 (a)	16.5 ± 0.3 (a)	1.6 ± 0.0 (a)	0.10 ± 0.01 (ab)	12.0 ± 0.4 (a)	0.25 ± 0.04 (b)	75.1 ± 7.5 (b)	15.0 ± 0.9 (b)	0.08 ± 0.01 (bc)	15.9 ± 0.8 (a)	26.1 ± 2.1 (a)	60.5 ± 1.1 (ab)
MSW	7.8 ± 0.1 (a)	13.2 ± 0.2 (c)	1.3 ± 0.0 (a)	0.07 ± 0.00 (ab)	10.5 ± 0.5 (b)	0.23 ± 0.01 (ab)	74.3 ± 4.7 (ab)	16.2 ± 0.6 (b)	0.10 ± 0.02 (bc)	15.5 ± 0.6 (a)	27.7 ± 4.1 (a)	61.3 ± 2.7 (ab)
FYM	7.4 ± 0.0 (b)	14.8 ± 0.4 (b)	1.4 ± 0.0 (a)	0.11 ± 0.01 (ab)	10.5 ± 0.4 (b)	0.24 ± 0.01 (ab)	74.1 ± 3.1 (ab)	15.5 ± 1.1 (b)	0.08 ± 0.02 (bc)	15.5 ± 0.8 (a)	25.3 ± 0.9 (a)	61.1 ± 5.3 (ab)
CN	6.8 ± 0.2 (d)	10.0 ± 0.3 (d)	1.0 ± 0.0 (a)	0.06 ± 0.01 (a)	8.5 ± 0.6 (c)	0.20 ± 0.02 (a)	72.5 ± 8.0 (a)	12.0 ± 0.8 (c)	0.07 ± 0.01 (c)	15.3 ± 0.9 (a)	21.3 ± 0.9 (b)	49.3 ± 2.1 (a)
Average contents in EU^a^						0.09	21.72	13.01	0.04	18.36	15.3	na
Median contents in France^b^						0.28	38.3	13.3	0.046	19.5	21.7	56.4

^a^Cited in Toth et al. (2016)

^b^BDETM (https://www.gissol.fr/le-gis/programmes/base-de-donnees-elements-traces-metalliques-bdetm-65, Accessed in 18 July 2019)

Several significant and lasting differences of soil physico-chemical properties appeared among the treatments at dates comprised between 2004 and 2015, depending on the element considered (Peltre et al. 2012; Obriot et al. 2016) (Appendix C in Electronic supplementary material). A decrease in soil organic C content (SOC) occurred in the control treatment CN and it has become significantly higher in GWS and BIOW treatments compared to CN since 2004 and since 2011 in the case of MSW and FYM treatments. In 2015, SOC content was significantly different among treatments and increased as follows: CN < MSW < FYM < BIO = GWS (Table 4). In 2015, the amended treatments always presented significant increases compared to the CN for the soil pH (up to 1 unit for BIOW and MSW), the organic carbon content (up to + 69% for GWS and + 65% for BIOW), and the cation exchange capacity (up to + 41% for BIOW). Those soil properties are known to influence the availability and mobility of TE, thus their bioavailability and toxicity (Harmsen 2007; Smolders et al. 2009; Smolders et al. 2012).

**Fig. 3:**
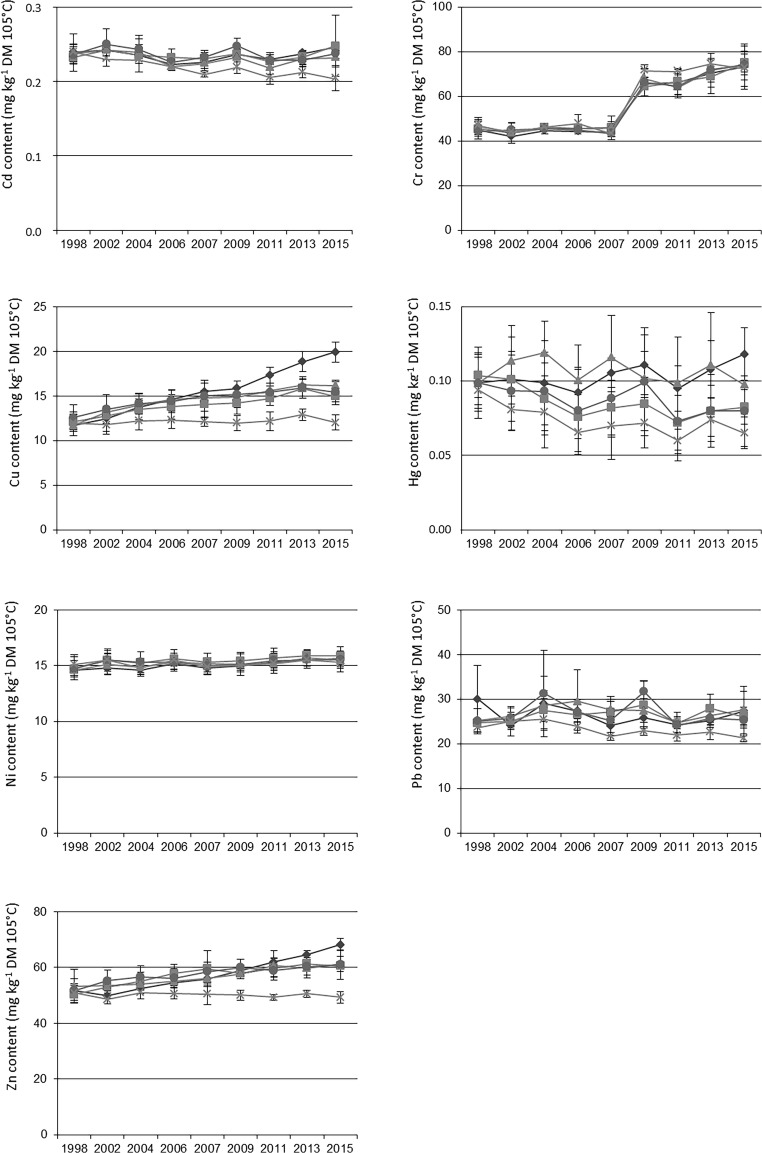
Evolution of trace element contents in the topsoil layer over the period 1998–2015 for Cd, Cr, Cu, Hg, Ni, Pb and Zn (mg kg^−1^ DM; DM for dry matter). Diamonds stand for co-compost of sewage sludge and green waste (GWS), squares stand for biowaste compost (BIOW), triangles stand for compost of residual municipal solid waste (MSW), circles stand for farmyard manure (FYM) and crosses stand for control (CN). The error bars are standard deviations

Figure 3 summarizes the evolution of TE content in the topsoil layer over the period 1998–2015 (detailed statistics are available in Appendix C):

- For Cd, a slight and generally not significant decrease occurred in the control treatment (− 17% between 1998 and 2015), while significant differences have been observed between OWP treatments and CN since 2011.
- A large increase of all Cr values appeared in 2009 compared to previous years probably coming from a change in the analytical methods in the laboratory. This made difficult to analyse the evolution in Cr content in the different treatments. No lasting significant differences appeared among treatments.
- Cu became significantly higher in the amended treatments compared to CN in 2004, namely after 3 OWP applications, with no significant differences between amended treatments until 2011, when the plots receiving the GWS compost became significantly above the other amended plots. Significant increase with time was observed for the soils receiving OWP, up to + 69% for GWS.
- For Hg, despite the low contents and relatively high standard errors compared to other elements, tendencies were observed with decrease observed in CN, BIOW and FYM treatments; significant differences have been observed with MSW and GWS > CN in 2004, 2006, 2007, 2011 and 2015 (only for GWS).
- For Ni, no significant differences were ever observed between the treatments and with years. Nevertheless, a slight but not significant increase was observed for the BIOW compost over the period 1998–2015.
- With respect to Pb, relatively large standard errors have been observed, since the beginning of the experiment, which can be due to initial spatial heterogeneity. Nevertheless, significant differences between all organic treatments and CN appeared only in 2015.
- Finally Zn content has become significantly larger in most organic treatments compared to CN in 2006, with the largest contents observed in the GWS treatment since 2013. Zn content increased significantly with time in soils receiving GWS (+ 32%), BIOW (+ 20%) and MSW (+ 15%).

In summary, the most conclusive significant differences of TE contents in the OWP compared to CN treatment have been observed: since 2004 for Cu and since 2006 for Zn due to significant increase with time in soils receiving OWP, since 2011 for Cd and 2015 for Pb as related to apparent decrease in the control treatment. Losses of TE applied to soil in OWP have been reported in previous studies, as a relation to a combination of small losses from leaching, erosion plus unexplained experimental errors and movement due to tillage operations (McGrath and Lane 1989; Berti and Jacobs 1998). This could explain decrease for Cd and Pb observed in the present study.

In 2015, the contents of Cu and Zn have increased with respect to 1998. As observed in the present study and as already stressed by Smith (2009), composts and sewage sludges contain larger contents of TE than the background values found in soil (Tables 2 and 4), so that their regular application to land gradually raises the total TE content of soil in the long term. These results confirmed the numerous previous studies that showed significant accumulation of Zn, Cu, and less often Cd and Pb in soil, after repeated applications of biosolids (Moolenaar and Beltramy 1998; Richards et al. 1998; Warman and Termeer 2005; Kirchman et al. 2017), or composts (Gigliotti et al. 1996; Delschen 1999; Weber et al. 2007; Alvarenga et al. 2017). McBride and Spiers (2001) showed that sewage sludge can lead to much higher soil accumulations of TE (e.g. Cu, Zn) compared to FYM and mineral fertilization. Our results also confirmed that TE content increases are slow and slight. Thus, long-term evaluations are required to evaluate precisely the effects of repeated applications of OWP on TE accumulation in soils, especially for some toxic TE such as Pb and Cd. Indeed short-term studies usually do not reveal TE increases in soils (Douglas et al. 2003).

To complete the Fig. 3, Table 4 presents average contents found after 9 applications of OWP in 2015 for the 5 treatments, as compared to the average contents found in the topsoil layer in Europe (Toth et al. 2016) and the median contents found in France (BDETM 2019). When comparing to contents found in Europe and France, TE contents measured in 2015 were globally in the range or slightly larger than usual contents found in Europe and France, as follows:

- Cu, Hg, Pb and Zn, in the range or slightly larger than average contents found in Europe and France, knowing that some agricultural practices are known to cause larger Cu concentrations in soils due to inputs of Cu-based pesticides used in vineyard and orchards (Belon et al. 2012).
- Cd, larger than average contents found in Europe and in the range of median contents found in France.
- Ni, slightly lower than average contents found in Europe and France.

### Effect of organic waste product applications on plant trace element contents

Table 5 summarizes the average contents of TE in wheat, maize and barley grains. Yields are also presented. Statistical analyses are detailed in Appendix I (Electronic supplementary data). The TE contents decreased in the order Zn > Cu > Cr > Ni. In addition, for toxic TE, i.e. Cd, Cr, Hg, Ni and Pb, most values were lower than the quantification limit, for the 3 species. A few significant differences appeared between the organic treatments with respect to CN or among the organic treatments, but not repeatedly in time. Compost application (GWS, BIOW, MSW) did not induce significant increase of TE content in grains for the 3 species studied. Indeed large variations between years were observed, as shown by large standard deviation. This finding was already noticed by Chaudri et al. (2007) who found annual differences in grain Cd uptake. Indeed agroclimatic conditions and varietal differences are important factors in the uptake of Cd by crops. For Cu and Zn, essential elements for plant nutrition, values were above the quantification limit. Average grain contents of TE found in QualiAgro were globally equal or below regulatory (Codex Alimentarius 2015) and published values for Cd, Hg, Ni and Pb; while contents of Cr, Cu and Zn were in the range of published values for grains (Kabata-Pendias and Pendias 2001; Feix and Tremel 2005) (Table 5).

**Table 5:** Average yield (t DM ha^−1^; DM for dry matter) and average trace element contents in consumed exported grains of wheat, barley and maize over the period 1998–2015 for Cd, Cr, Cu, Hg, Ni, Pb and Zn (mg kg^−1^ DM), with the following: mean values ± standard deviation which stands for the year variation; co-compost of sewage sludge and green waste (GWS), biowaste compost (BIOW), compost of residual municipal solid waste (MSW), farmyard manure (FYM), no organic amendment (CN)

Treatment	Species	Yield	Cd	Cr	Cu	Hg	Ni	Pb	Zn
		t DM ha^−1^	mg kg^−1^ DM						
GWS	Wheat 1998–2013	8.0 ± 0.7	0.015 ± 0.003 ^(<)^	0.22 ± 0.13 ^(<)^	3.0 ± 0.5	0.003 ± 0.004 ^(<)^	0.11 ± 0.08 ^(<)^	0.12 ± 0.08 ^(<)^	20.0 ± 3.9
BIOW	Wheat 1998–2013	7.9 ± 0.6	0.014 ± 0.006 ^(<)^	0.26 ± 0.16 ^(<)^	3.3 ± 0.4	0.003 ± 0.004 ^(<)^	0.12 ± 0.05 ^(<)^	0.12 ± 0.09 ^(<)^	20.4 ± 3.7
MSW	Wheat 1998–2013	7.9 ± 0.7	0.016 ± 0.005 ^(<)^	0.23 ± 0.15 ^(<)^	3.5 ± 0.5	0.003 ± 0.005 ^(<)^	0.14 ± 0.05 ^(<)^	0.16 ± 0.09 ^(<)^	21.5 ± 3.3
FYM	Wheat 1998–2013	8.1 ± 0.7	0.018 ± 0.005 ^(<)^	0.21 ± 0.10 ^(<)^	3.2 ± 0.5	0.003 ± 0.004 ^(<)^	0.13 ± 0.08 ^(<)^	0.15 ± 0.10 ^(<)^	21.3 ± 3.4
CN	Wheat 1998–2013	7.2 ± 0.7	0.020 ± 0.009 ^(<)^	0.22 ± 0.12 ^(<)^	3.3 ± 0.5	0.002 ± 0.004 ^(<)^	0.21 ± 0.11 ^(<)^	0.14 ± 0.09 ^(<)^	20.0 ± 3.6
GWS	Barley 2007 & 2015	7.2 ± 0.3	0.003 ± 0.002 ^(<)^	0.19 ± 0.27 ^(<)^	4.7 ± 1.1	0.001 ± 0.001 ^(<)^	0.10 ± 0.13 ^(<)^	0.06 ± 0.03 ^(<)^	29.9 ± 2.5
BIOW	Barley 2007 & 2015	6.7 ± 0.3	0.003 ± 0.002 ^(<)^	0.17 ± 0.24 ^(<)^	4.8 ± 1.0	0.001 ± 0.001 ^(<)^	0.06 ± 0.04 ^(<)^	0.05 ± 0.02 ^(<)^	27.9 ± 0.0
MSW	Barley 2007 & 2015	6.6 ± 1.1	0.006 ± 0.001 ^(<)^	0.19 ± 0.26 ^(<)^	5.0 ± 1.2	0.001 ± 0.001 ^(<)^	0.08 ± 0.05 ^(<)^	0.06 ± 0.01 ^(<)^	29.7 ± 0.7
FYM	Barley 2007 & 2015	6.8 ± 1.3	0.005 ± 0.002 ^(<)^	0.18 ± 0.26 ^(<)^	5.2 ± 1.6	0.001 ± 0.000 ^(<)^	0.09 ± 0.05 ^(<)^	0.08 ± 0.03 ^(<)^	28.7 ± 2.4
CN	Barley 2007 & 2015	5.7 ± 1.0	0.007 ± 0.003 ^(<)^	0.18 ± 0.22 ^(<)^	4.4 ± 0.6	0.001 ± 0.000 ^(<)^	0.08 ± 0.00 ^(<)^	0.25 ± 0.22 ^(<)^	25.3 ± 1.7
GWS	Maize 1999–2014	9.1 ± 0.7	0.005 ± 0.006 ^(<)^	0.07 ± 0.07 ^(<)^	2.0 ± 0.5	0.0002 ± 0.0004 ^(<)^	0.28 ± 0.26 ^(<)^	0.05 ± 0.05 ^(<)^	17.8 ± 2.4
BIOW	Maize 1999–2014	9.1 ± 0.7	0.006 ± 0.007 ^(<)^	0.08 ± 0.06 ^(<)^	2.2 ± 0.4	0.0002 ± 0.0002 ^(<)^	0.20 ± 0.11 ^(<)^	0.06 ± 0.06 ^(<)^	19.2 ± 2.5
MSW	Maize 1999–2014	8.9 ± 1.0	0.008 ± 0.008 ^(<)^	0.11 ± 0.08 ^(<)^	2.2 ± 0.5	0.0003 ± 0.0004 ^(<)^	0.22 ± 0.11 ^(<)^	0.06 ± 0.04 ^(<)^	19.9 ± 3.2
FYM	Maize 1999–2014	9.3 ± 1.0	0.006 ± 0.005 ^(<)^	0.08 ± 0.06 ^(<)^	2.1 ± 0.5	0.0002 ± 0.0003 ^(<)^	0.22 ± 0.11 ^(<)^	0.06 ± 0.05 ^(<)^	19.2 ± 3.4
CN	Maize 1999–2014	8.1 ± 1.9	0.006 ± 0.005 ^(<)^	0.09 ± 0.08 ^(<)^	2.2 ± 0.5	0.0004 ± 0.0006 ^(<)^	0.26 ± 0.09 ^(<)^	0.10 ± 0.10 ^(<)^	19.1 ± 3.5
Feix and Tremel 2005	Barley grain		0.02	0.02	5.0	0.01	0.20	0.45	28
	Wheat grain		0.03	0.10	4.5	0.01	0.30	0.50	25
Kabata-Pendias and Pendias 2001	Grain contents		0.02–0.04	0.2	1.8–10		0.14–0.90	0.18–0.40	23–37
Codex Alimentarius 2015^a^	Cereal grains		0.1					0.2	
	Wheat		0.2						

^(<)^at least half of values were inferior to the quantification limit

^a^Contents expressed as milligram per kilogram of fresh matter

These results confirmed that there was few or no effect of repeated application of OWP on TE contents in cereal grains (Businelli et al. 1996; Gigliotti et al. 1996; Smith 2009; Alvarenga et al. 2017). Plant physiology tends to regulate TE in the shoots and the grains so that most elements do not reach levels which could cause even chronic toxicity to plants or consumers (McBride 1995; Chaney 2012). The exception is for some elements, e.g. Cd, which are readily absorbed and translocated to shoots without causing phytotoxicity, and can be transferred in the food chain (Chaney 2012). Another reason explaining the lack of differences between treatments is the zone of root uptake which is often deeper than the plough layer (McBride 1995).

### Mass balances

The mass balance approach is useful to evaluate the sustainability of a system, and to anticipate depletion and accumulation of TE (Moolenaar et al. 1997b; Yu et al. 2011). Mass balance calculations can thus be used to assess the impacts of cropping practices, including spreading of OWP, on TE contents in soils (i.e. accumulation, steady-state, or depletion). It considers the major inputs and outputs. In the case of QualiAgro, we considered atmospheric depositions and OWP inputs as inputs, and crop uptake and leaching as outputs. In the present calculation, the impact of surface runoff and erosion was not considered. Inputs of TE via pesticides, mineral N fertilizers and organic fertilizers were also neglected. All calculations are presented in Electronic supplementary material.

**Fig. 4:**
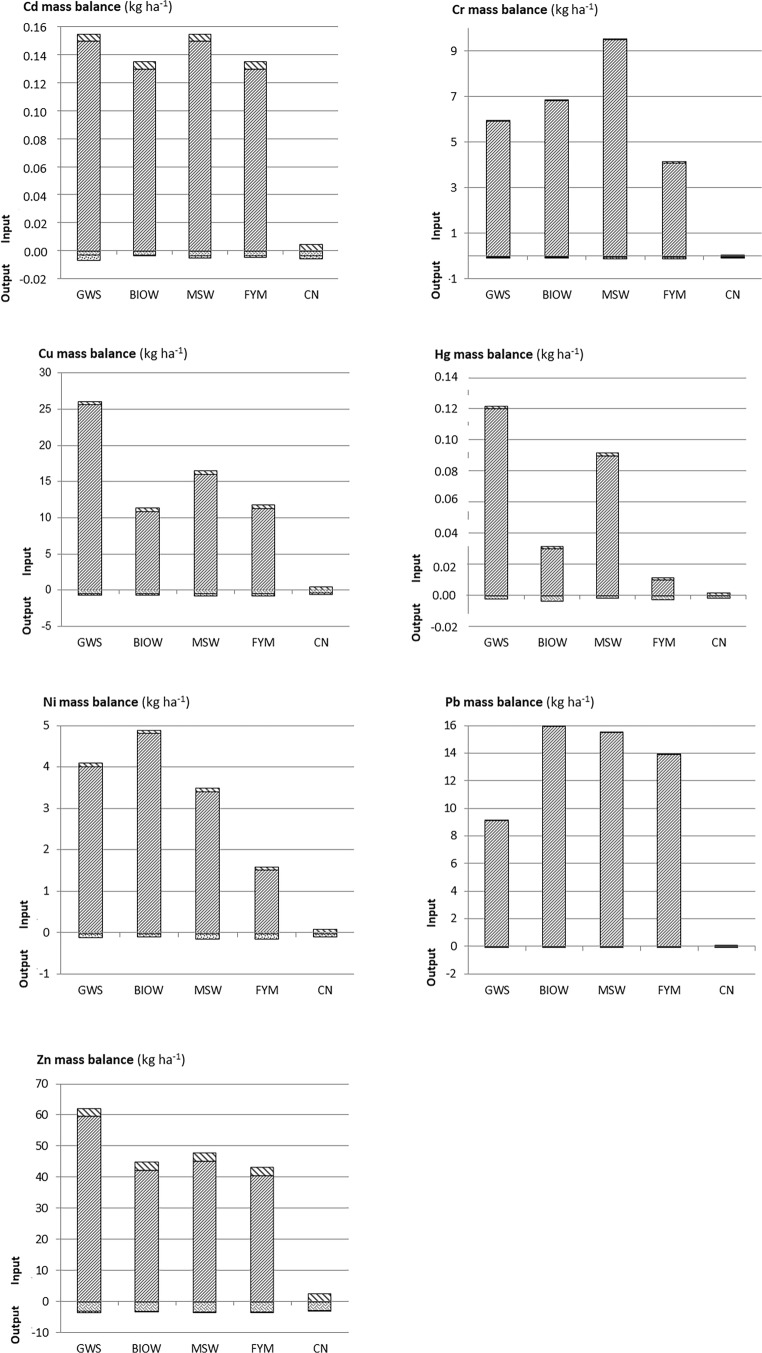
Mass balance of fluxes between compartments over the period 1998–2015, for Cd, Cr, Cu, Hg, Ni, Pb and Zn (kg ha^−1^). With co-compost of sewage sludge and green waste (GWS), biowaste compost (BIOW), compost of residual municipal solid waste (MSW), farmyard manure (FYM) and control (CN). Inputs fluxes stand for atmospheric deposits and OWP inputs (respectively bold hatchings at the top and fine hatchings at the bottom of input), output fluxes stand for plant exportations and water drainage (respectively zigzag at the top and little squares at the bottom of output)

Field mass balance for the period 1998–2015 is presented in Fig. 4 (Appendix J in Electronic supplementary material), with cumulated fluxes for inputs and outputs. Input of TE by OWP were much larger compared to atmospheric inputs and outputs (i.e. crop offtake and leaching). It represented up to 17% of the initial soil stock for Cd (MSW and GWS), 56% for Cu (GWS), 34% for Hg (GWS), 8% for Ni (BIOW), 17% for Pb (BIOW) and 30% for Zn (GWS) (Table 6). Among OWP, FYM most often presented the smallest TE mass balances while GWS and MSW presented the largest ones.

**Table 6:** Cumulated input through organic waste product spreading (Input OWP flux), soil stock variation (Delta stock) and mass balance (Balance inputs − outputs) of trace elements over the period 1998–2015 for Cd, Cr, Cu, Hg, Ni, Pb and Zn (kg ha^−1^), with the following: the percentage of the considered flux with respect to the initial soil stock written into brackets; co-compost of sewage sludge and green waste (GWS), biowaste compost (BIOW), compost of residual municipal solid waste (MSW), farmyard manure (FYM), no organic amendment (CN); nc stands for not concerned

		Cd	Cu	Hg	Ni	Pb	Zn
		kg ha^−1^					
GWS	Input OWP flux	0.15 (17.0%)	25.6 (56.1%)	0.12 (34.0%)	4.0 (7.1%)	9.1 (8.4%)	59.6 (30.4%)
	Delta stock	0.05 (5.2%)	29.2 (63.9%)	0.09 (25.8%)	3.0 (5.3%)	− 5.0 (− 4.6%)	60.8 (31.0%)
	Balance inputs − outputs	0.15 (16.8%)	25.4 (55.8%)	0.12 (33.9%)	4.0 (7.0%)	9.1 (8.4%)	58.7 (30.0%)
BIOW	Input OWP flux	0.13 (15.1%)	10.9 (23.3%)	0.03 (8.1%)	4.8 (8.5%)	15.9 (17.4%)	42.3 (22.1%)
	Delta stock	0.07 (8.6%)	10.0 (21.4%)	− 0.06 (− 15.1%)	3.7 (6.4%)	8.0 (8.7%)	38.1 (19.9%)
	Balance inputs − outputs	0.13 (15.2%)	10.7 (22.9%)	0.03 (7.6%)	4.8 (8.4%)	15.9 (17.4%)	41.6 (21.7%)
MSW	Input OWP flux	0.15 (17.1%)	16.0 (35.2%)	0.09 (25.5%)	3.4 (6%)	15.5 (16.7%)	45.3 (22.6%)
	Delta stock	− 0.005 (− 0.6%)	15.4 (33.8%)	0.02 (4.7%)	2.5 (4.5%)	11.4 (12.3%)	30.5 (15.2%)
	Balance inputs − outputs	0.15 (17.1%)	15.7 (34.5%)	0.1 (25.6%)	3.3 (5.9%)	15.5 (16.7%)	44.4 (22.2%)
FYM	Input OWP flux	0.13 (14.8%)	11.3 (23.4%)	0.01 (2.8%)	1.5 (2.6%)	13.9 (15.0%)	40.6 (20.7%)
	Delta stock	0.022 (2.5%)	10.3 (21.3%)	− 0.05 (− 13.8%)	2.2 (3.9%)	3.3 (3.5%)	35.5 (18.1%)
	Balance inputs − outputs	0.13 (14.9%)	11.0 (22.8%)	0.01 (2.6%)	1.4 (2.5%)	13.9 (15.0%)	39.7 (20.3%)
CN	Input OWP flux	nc	nc	nc	nc	nc	nc
	Delta stock	− 0.11 (− 12.5%)	− 0.6 (− 1.2%)	− 0.09 (− 26.7%)	0.1 (0.2%)	− 7.1 (− 8.1%)	− 5.7 (− 3%)
	Balance inputs − outputs	− 0.0001 (0.0%)	− 0.05 (− 0.1%)	0.0003 (0.1%)	− 0.01 (0.0%)	0.002 (0.0%)	− 0.5 (− 0.3%)

Outputs were generally very low. For Cr, Pb and Zn, the percentages of leaching were lower than 2% of TE OWP inputs. Leaching represented respectively for Cd, Cu and Ni: up to 2.3% of Cd inputs flux by GWS, 2.7% of Cu inputs by FYM and 7.8% of Ni inputs by FYM. For Cu and Zn, for which TE content was higher than the quantification limits in grains and residues, crop uptake represented 1.8% (GWS) to 4.4% (BIOW) of the Cu inputs by OWP; and 5.1% (GWS) to 7.3% (BIOW) for Zn. For the other TE, for which [TE]_crop_ in grains was globally lower than the quantification limit, crop uptake has been assessed to represent less than 2% of TE inputs by OWP (except for Hg in BIOW).

For all OWP amendments, the resulting positive mass balance (inputs − outputs) was similar to the input flux by OWP for Cd, Hg, Ni and Pb (Table 6). For Cu and Zn, the calculated outputs resulted in slight differences between the positive mass balance and the input flux by OWP (respectively − 0.2 to 0.3 kg ha^−1^ and −0.7 to 0.9 kg ha^−1^). Globally over the period 1998–2015, mass balance corresponded to the delta soil stock for Cu, Ni and Zn (Table 6; Appendix E, Table E.2). For Cd and Hg, mass balance was larger than the delta soil stock, suggesting losses such as leaching for Cd or evaporation for Hg (Cobbett and Van Heyst 2004). For Cd, content in the topsoil layer remained around 0.24 mg kg^−1^ in OWP treatments in 1998–2015, while it decreased to 0.20 mg kg^−1^ in the control treatment in 2015 (Tables 1 and 4). Putative leaching of Cd observed in the control treatment could occur for each treatment. In OWP treatments this could explain the stability observed in the topsoil layer Cd content in 1998–2015 despite slight positive mass balance (Table 6). For Pb, mass balance was also larger than the delta soil stock. However, there were large variabilities in soil contents so that it was difficult to conclude. In contrary to amended plots, negative delta soil stocks have been measured in the control treatment. This depletion in control soils was as follows: Pb > Zn > Cu > Cd > Hg. The calculated mass balance in this control treatment was less negative than the measured decreased in soil stocks. Such differences could be due to overestimation of atmospheric inputs or underestimation of leaching outputs, since these fluxes have not been measured on our site or not during the whole period in case of leaching.

### Prospective evaluation: threshold values and “very” long-term simulation of total soil TE content

As said by Lofts et al. (2007): “At what point does this contamination become pollution (contamination that results in adverse biological effects)?” We tried to answer that question at first by (i) estimating threshold values considered as soil safe limits, and then by (ii) simulating the long-term accumulation of TE in soils exposed to a precise and reasonable scenario of OWP amendments.

In the present study, as explained in “Threshold contents for trace elements in soil” section, we used the “Threshold calculator for metals in soils” (Oorts et al. 2018) to estimate threshold values considered as PNEC. The “Threshold calculator” is based on species sensitivity distributions (SSD) found in the literature and applied to estimate hazardous concentration (HC), taking into account influencing soil physico-chemical properties. In the present study, hazardous concentration for 5% of the species (HC_5_) derived from EC10 distributions (Effective concentration for which 10% of the community is affected by the concentration of the trace element) has been chosen as it is proposed as a PNEC and “safe” concentration (Oorts 2018).

The results of these calculations, using soil parameters measured in 2015, are presented in Table 7. After 9 applications of OWP, the TE contents in the topsoil layer in 2015 (Table 4) were below the HC_5_ of EC_10_, or PNEC values, for Cd, Cu, Ni, Pb and Zn for the 5 treatments. It can be noticed that PNEC values for the control treatment were always below those found for OWP amended soils, except for Cd whose PNEC are similar in all treatments. This could be related to the so-called sludge protection mechanisms, also valid for all organic amendments (sewage sludge, manure, compost) as shown by Smolders et al. (2012). Addition of organic matter can have protective effects by decreasing metal bio/availability, through increasing soil retention factors, including pH, soil organic matter and CEC.

**Table 7:** Threshold values carried out by the threshold calculator for the 5% probability level (5% Hazardous concentration or HC_5_) of the reliable effective concentration EC_10_ (i.e. effective concentration for which 10% of the community is affected by the concentration of the trace element), with the following: mean values per treatment ± standard deviation of the four replicates (except for Cd for which the calculator calculations were not available for raw data); co-compost of sewage sludge and green waste (GWS), biowaste compost (BIOW), compost of residual municipal solid waste (MSW), farmyard manure (FYM), no organic amendment (CN)

Metal	Treatment	HC_5_ of EC_10_ values
		mg kg^−1^ DM
Cd	GWS	2.7
	BIO	2.7
	MSW	2.7
	FYM	2.7
	CN	2.7
Cu	GWS	77.9 ± 2.0
	BIO	75.2 ± 1.3
	MSW	67.9 ± 1.1
	FYM	72.5 ± 1.5
	CN	64.2 ± 2.0
Ni	GWS	52.5 ± 2.5
	BIO	59.4 ± 2.3
	MSW	51.1 ± 2.6
	FYM	51.2 ± 2.2
	CN	40.6 ± 3.3
Pb	GWS	240.7 ± 11.7
	BIO	273.3 ± 10.4
	MSW	234.4 ± 12.6
	FYM	234.9 ± 10.8
	CN	182.5 ± 16.8
Zn	GWS	142.0 ± 4.6
	BIO	166.3 ± 4.6
	MSW	151.7 ± 5.8
	FYM	146.4 ± 4.5
	CN	116.8 ± 9.1

The quotients of total TE contents divided by the soil PNEC values stand in the range 0.07–0.09 for Cd, 0.19–0.26 for Cu, 0.27–0.38 for Ni, 0.10–0.12 for Pb and 0.36–0.48 for Zn. They suggest that TE contents in soils receiving repeated applications of OWP would be safe for a long time for Cd, Pb, even for Cu and Ni. For Zn, calculations showed increasing risks associated with the contents of Zn in soils which tend towards the protective threshold in the following order: GWS > CN > FYM > MSW > BIOW. Indeed Zn is the element in OWP-treated soils identified as the main concern in relation to eco-toxicity and long-term soil fertility (Smith 2009; Yoshida et al. 2018).

Results confirmed that repeated application of OWP, even in large amounts such as those applied in the QualiAgro experiment, did not alter the soil quality in terms of TE contents since they remained far below the calculated protective threshold value for Cd, Cu, Ni, Pb and Zn. However, based on the observation of the correspondence between the balance approach and the evolution of TE stocks in the topsoil layer during 17 years, we aimed at completing our evaluation of such practices at a longer time scale, i.e. about 100 years.

The evolution of the total TE concentrations in topsoil layer has thus been simulated until 2100 using the Eq. (8) (“Simulation of soil trace element content” section) and the average measured inputs and outputs summarized in Appendix J, considering approaching usual agronomic spreading amounts of OWP (i.e. 1.5 lower than amounts of OWP applied in the QualiAgro device). This scenario would be considered as an extreme local scenario in which OWP would be applied during a period of 100 years at an average rate of 30 t ha^−1^ per 3 years for farmyard manure and 20–30 tons ha^−1^ per 3 years for composts. In practice, this extreme local scenario would not occur due to the need of maintaining adequate balance of macronutrients in the soils. According to Moolenaar et al. (1997b), the time scale of one century was considered here, since calculations for longer periods should be avoided. Calculations presented in Appendix K showed that the model gave good predictions of [TE]_top,*i*,2015_ for Cr, Cu, Ni and Zn (i.e. deviations < 7% in absolute value), acceptable prediction for Cd and Pb (< 21% of deviation) and no accuracy for Hg which presented large variation in results obtained in QualiAgro, as presented previously. Differences between the model and measured data in QualiAgro could be partly explained by losses for Cd such as underestimated leaching measurements, variability in data for Pb and Hg, and volatilization of Hg. Other limits could also alter simulated results, such as neglecting erosion and using fixed average values, particularly for leaching TE.

**Fig. 5:**
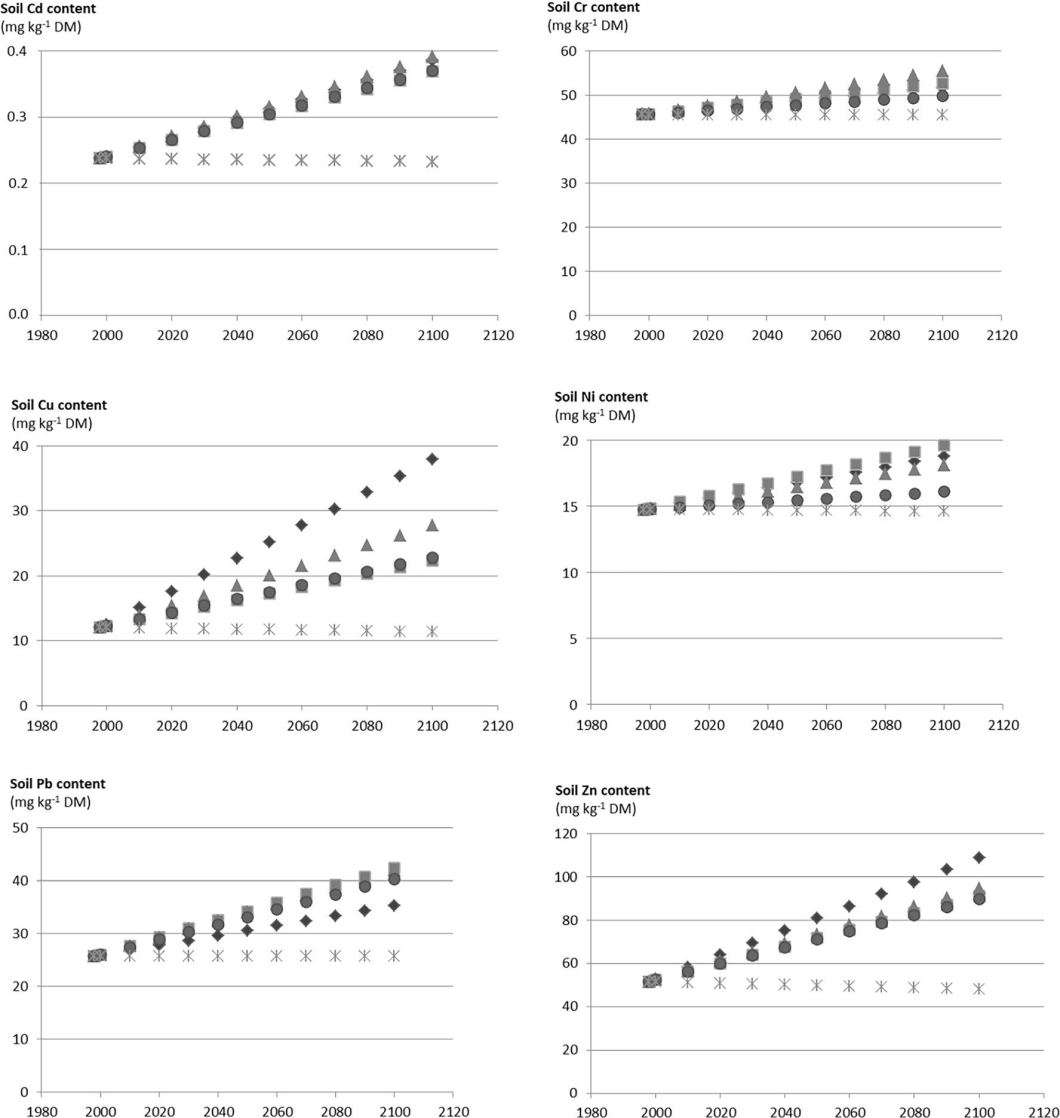
Long-term simulation of the topsoil layer content of Cd, Cr, Cu, Ni, Pb and Zn (mg kg^−1^ DM; DM for dry matter), considering the example of an extreme local scenario. Diamonds stand for co-compost of sewage sludge and green waste (GWS), squares stand for biowaste compost (BIOW), triangles stand for compost of residual municipal solid waste (MSW), circles stand for farmyard manure (FYM) and crosses stand for control (CN)

The outputs of the calculation using usual agronomic amounts of OWP are presented in Fig. 5 for the evolution of the total content in the topsoil layer for Cd, Cr, Cu, Ni, Pb and Zn. Simulation results showed increases in concentrations in 2100 until 0.4 mg Cd kg^−1^ DM (GWS, MSW), 56 mg Cr kg^−1^ DM (MSW), 38 mg Cu kg^−1^ DM (GWS), 20 mg Ni kg^−1^ DM (BIOW), 42 mg Pb kg^−1^ DM (BIOW, MSW) and 109 mg Zn kg^−1^ DM (GWS). With simulations from 1998 to 2100 using an extreme local scenario, application of OWP in lands could lead to long-term accumulation of TE in the topsoil layer up to + 64% for Cd (MSW), + 22% for Cr (MSW), + 215% for Cu (GWS), + 33% for Ni (BIOW), + 63–65% for Pb (BIOW, MSW) and + 111% for Zn (GWS).

In the case of Cd, Sterckeman et al. (2018) found 3% of increase by the end of the next century for the average Cd content in French soils under annual crops, while Six and Smolders (2014) found a decrease in European soil Cd content for a 100-year period. In both studies, simulations of the soil Cd content was based on average inputs/outputs estimated at larger scale (i.e. sub-region, region), and on Cd concentration in leaching water predicted from soil pH, Cd and organic C content. Contrary to Sterckeman et al. (2018), fixed values for [Cd]_lea_ and *Q*_lea_ were used in the simulation model in the Eq. (8). The model used in the present study, validated with measured data, could be considered as an extreme local scenario, nevertheless possible. This could explain the larger increase in Cd contents found in the present study compared to the ones cited above. Belon et al. (2012) underlined the lack of output data for crop uptake and leaching. Any accurate simulation approach should also consider suitable inputs and outputs, as well as its outputs validation according to measured data, especially in long-term field experiments such as QualiAgro. In fact in the work of Sterckeman et al. (2018), long-term experiments (including QualiAgro and PROspective devices of the SOERE PRO network) were taken as reference to adjust leaching predictions.

Finally, trace element contents simulated in 2100 (i.e. [TE]_top,*i*,2100_) remained below the thresholds values of HC_5_EC_10_ (i.e. PNEC) presented in Table 7. Nevertheless, these results also showed that applications of OWP in usual agronomic amounts would increase the soil Zn content to levels close to the corresponding safe threshold. With TE contents in OWP conform to existing French regulations (NFU 44-051, 2006 and NFU 44-095, 2002), and if OWP are applied in appropriate amounts, repeated application of OWP should not lead to critical contents in soils in the long term (Delschen 1999). Indeed, estimated increases of TE in the topsoil layer were lower than the TE content increase found by Charlton et al. (2016) to affect the microbial biomass in soil.

## Conclusion and perspectives

OWP applications to lands offer valuable advantages. The BIOW compost could be considered as a good soil amendment, for acidic soil pH correction and soil organic matter increase; the GSW compost can be used also as soil organic amendment but as well for phosphorus and nitrogen fertilization; the FYM could be considered as an organic amendment and fertilizer and the MSW compost could be used as source of organic matter implied in stimulation of biological activities and aggregate stability. However, TE contents in OWP are often larger than those found in agricultural soils. The GWS and MSW composts were the OWP with the largest TE contents, while the farmyard manure tended to have the lowest TE contents. In QualiAgro, repeated application of OWP led to significant accumulation of Zn and Cu in the topsoil layer, especially with the compost of green waste and sludges (GWS). Nevertheless, TE contents in soils did not overpass calculated protective threshold values for Cd, Cu, Ni, Pb and Zn. Among OWP studied, the mass balance represented up to 17% of the initial soil stock for Cd (MSW and GWS), 56% for Cu (GWS), 34% for Hg (GWS), 8% for Ni (BIOW), 17% for Pb (BIOW) and 30% for Zn (GWS). Positive mass balances corresponded to the observed increases in delta soil stocks, especially for Cu and Zn. Weak outputs (i.e. leaching, crop offtakes) were observed compared to the OWP inputs. The QualiAgro data confirmed previous studies showing little or no effect of repeated application of OWP on TE contents in cereal grains. Values were below or in the range of usual TE contents found in grains. Simulation results using the average inputs/outputs measured in the QualiAgro field experiment showed increases in concentrations in 2100 up to 0.4 mg Cd kg^−1^ DM (GWS, MSW), 56 mg Cr kg^−1^ DM (MSW), 38 mg Cu kg^−1^ DM (GWS), 20 mg Ni kg^−1^ DM (BIOW), 42 mg Pb kg^−1^ DM (BIOW, MSW) and 109 mg Zn kg^−1^ DM (GWS).

The mass balance calculations pointed out the difficulty to collect complete data set from the beginning of the experimentation and the necessity to estimate some data. Complete monitoring and saving samples from the beginning of an experiment at same sampling periods for topsoil layer and the soil sub-layer would permit to avoid missing data.

The mass balance approach could be used to calculate input rates of TE through OWP application to avoid potential adverse effects of TE accumulation in soils. To maintain this state, regulation should consider lower TE contents in OWP and/or lower spreading amounts, based on studies such as the QualiAgro one. In the case of Zn, considering usual amounts of OWP (< 10 t DM ha^−1^ per 2 years) and simulated evolution of soil contents, limiting input flux of Zn by OWP applied in lands would lead to avoid long-term Zn accumulation in the topsoil layer overpassing protective threshold. For instance, the regulatory limit of total Zn input flux could be fixed at a lower level than 30 kg ha^−1^ per 10 years (e.g. at the level of 20 kg ha^−1^ per 10 years).

Such study should be applied in the future in other field experiments integrated in the SOERE PRO (network of long-term experiments dedicated to the study of impacts of organic waste product recycling). This would (i) complete mass balances and references of average inputs/outputs that could be used in regional/national-scale evaluations and (ii) validate the simulation model used in the present study for various agro-pedo-climatic contexts and different OWP. In addition, for complete examination of the TE status in soils receiving repeated applications of OWP, the present study should be done for wider range of TE and completed by measurements of TE bioavailability.

## Electronic supplementary material

ESM 1 (DOCX 465 kb)
