# Supplementary material for: Mass balance and long-term soil accumulation of trace elements in arable crop systems amended with urban composts or cattle manure during 17 years
Source: Environ Sci Pollut Res Int. 2019 Dec 17;27(5):5367–86. doi: 10.1007/s11356-019-07166-8 (PMC7028826; doi:10.1007/s11356-019-07166-8)
Supplement: Supplementary file 1 — (DOCX 465 kb) [file 11356_2019_7166_MOESM1_ESM.docx]

**Mass balance and long-term soil accumulation of trace elements in arable crop systems amended with urban composts or cattle manure during 17 years**

***Journal: Environmental Science and Pollution Research***

Authors: Aurélia Marcelline Michaud^ab*^, Philippe Cambier^a^, Valérie Sappin-Didier^c^, Valentin Deltreil^d^, Vincent Mercier ^a^, Jean-Noël Rampon^a^, Sabine Houot^a^

^a^ UMR ECOSYS, INRA, AgroParisTech, Université Paris-Saclay, 78850, Thiverval-Grignon, France

^b^ UMR SAS, INRA, AgroCampusOuest, 65 rue de Saint Brieuc, 35042 Rennes, France

^c^ UMR ISPA, INRA, 33883 Villenave d’Ornon

^d^ Veolia recherche & innovation, Chemin de la digue, 78603, Maisons-Laffitte, France

* Corresponding author: [aurelia.michaud@inra.fr](mailto:aurelia.michaud@inra.fr)

**Electronic Supplementary Material**

# Appendix A: Field management and sampling periods

*
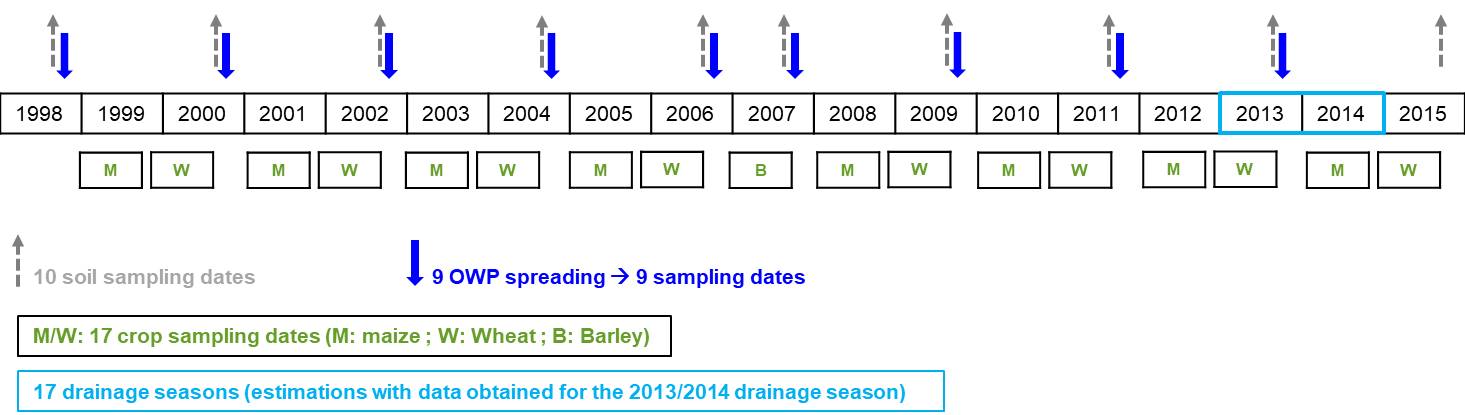
*

*Organic waste products (OWP)*

# Appendix B: Soil density and calculation of soil mass in the top soil layer for the period 1998-2015

### Table B.1: Soil bulk density (d_top,i,n_) in the topsoil layer measured in 1998, 2004, 2009 and 2013.

With mean values ± standard deviation of the four replicates.

*Co-compost of sewage sludge and green waste (GWS), biowaste compost (BIOW), compost of residual municipal solid waste (MSW), farmyard manure (FYM), no organic amendment (CN); Organic waste products (OWP).*

|  | **Soil bulk density, t m^-3^** | | | |
| --- | --- | --- | --- | --- |
| **Treatment** | **1998** | **2004** | **2009** | **2013** |
| GWS | 1.23 ± 0.10 (a) | 1.29 ± 0.03 (a) | 1.31 ± 0.03 (b) | 1.35 ± 0.03 (b) |
| BIOW | 1.26 ± 0.03 (a) | 1.30 ± 0.02 (a) | 1.30 ± 0.00 (b) | 1.38 ± 0.03 (b) |
| MSW | 1.29 ± 0.07 (a) | 1.30 ± 0.01 (a) | 1.34 ± 0.02 (ab) | 1.37 ± 0.01 (b) |
| FYM | 1.27 ± 0.03 (a) | 1.30 ± 0.03 (a) | 1.33 ± 0.03 (ab) | 1.38 ± 0.03 (b) |
| CN | 1.27 ± 0.07 (a) | 1.32 ± 0.04 (a) | 1.39 ± 0.02 (a) | 1.48 ± 0.02 (a) |
|  |  |  |  |  |
| OWP (all) | 1.26* ± 0.02 | 1.30 ± 0.00 | 1.32 ± 0.02 | 1.37 ± 0.01 |
| CN | 1.27* ± nc | 1.32 ± nc | 1.39 ± nc | 1.48 ± nc |

*nc: not concerned*

** Average soil bulk density of 1.26 measured in 1998.*

Bilateral Dunn’s non-parametric test was performed to identify significant differences between treatments (Abc letters stand for significant difference between treatments).

### Part B.2: Polynomial equation used to estimate the lacking values of soil density for the period 1998-2015.

With the equation,

For the OWP treatments (i.e. GWS, BIOW, MSW, FYM), y = 0.0002x² + 0.0041x + 1.26; r² = 0.9778

For the control (CN) treatment, y = 0.0006x² + 0.0053x + 1.26; r² = 0.9952

### Table B.3: Estimated values and percentage of accuracy between estimated values and measured data.

With measured and estimated bulk density for the period 1998-2015. The following data which are in **bold and underlined** were used in calculation of the soil stocks of trace elements in the topsoil layer (Q_top,i,n_).

|  |  | **Soil bulk density, t m^-3^** | | | | | | | | |
| --- | --- | --- | --- | --- | --- | --- | --- | --- | --- | --- |
|  | **Year** | **1998** | **2002** | **2004** | **2006** | **2007** | **2009** | **2011** | **2013** | **2015** |
| Measured data | OWP | **1.26** |  | **1.30** |  |  | **1.32** |  | **1.37** |  |
| Measured data | CN | **1.27** |  | **1.32** |  |  | **1.39** |  | **1.48** |  |
| Estimated data | OWP | 1.26 | **1.28** | 1.29 | **1.31** | **1.31** | 1.33 | **1.35** | 1.37 | **1.39** |
| Estimated data | CN | 1.26 | **1.29** | 1.31 | **1.34** | **1.36** | 1.39 | **1.43** | 1.47 | **1.52** |
| % of accuracy* | OWP | **99.8** |  | **99.5** |  |  | **100.5** |  | **99.6** |  |
| % of accuracy* | CN | **99.3** |  | **99.8** |  |  | **100.1** |  | **99.7** |  |

** Percentage of accuracy of the estimated values compared to the data measured in 1998, 2004, 2009 and 2013.*

### Table B.4: Calculation of the soil mass of the topsoil layer (W_top,i,n_)

With soil density (d_top,i,n_), soil layer depth, soil mass and differences between the topsoil mass of the control treatment in 2015* which was the largest one of all (W_top,CN,2015_) and the topsoil mass calculated for each treatment per year (W_top,i,n_).

To calculate the stock of TE on a fixed soil mass basis, W_top,CN,2015_, we must add the stock corresponding to the sublayer progressively mixed up between 1998 and 2015. The following soil mass differences were thus used to estimate the trace element soil stock of the sub-layer to be added to the trace element soil stock of the topsoil (see Appendix E).

*Co-compost of sewage sludge and green waste (GWS), biowaste compost (BIOW), compost of residual municipal solid waste (MSW), farmyard manure (FYM), no organic amendment (CN).*

|  |  | **Soil density** | **Soil depth** | **Soil mass** | **Soil mass differences** |
| --- | --- | --- | --- | --- | --- |
| **Treatment** | **Year** | t m^-3^ | m | t ha^-1^ | t ha^-1^ |
| GWS | 1998 | 1.26 | 0.25 | 3150 | 650 |
| GWS | 2002 | 1.28 | 0.25 | 3200 | 600 |
| GWS | 2004 | 1.30 | 0.25 | 3250 | 550 |
| GWS | 2006 | 1.31 | 0.25 | 3275 | 525 |
| GWS | 2007 | 1.31 | 0.25 | 3275 | 525 |
| GWS | 2009 | 1.32 | 0.25 | 3300 | 500 |
| GWS | 2011 | 1.35 | 0.25 | 3375 | 425 |
| GWS | 2013 | 1.37 | 0.25 | 3425 | 375 |
| GWS | 2015 | 1.39 | 0.25 | 3475 | 325 |
| BIOW | 1998 | 1.26 | 0.25 | 3150 | 650 |
| BIOW | 2002 | 1.28 | 0.25 | 3200 | 600 |
| BIOW | 2004 | 1.30 | 0.25 | 3250 | 550 |
| BIOW | 2006 | 1.31 | 0.25 | 3275 | 525 |
| BIOW | 2007 | 1.31 | 0.25 | 3275 | 525 |
| BIOW | 2009 | 1.32 | 0.25 | 3300 | 500 |
| BIOW | 2011 | 1.35 | 0.25 | 3375 | 425 |
| BIOW | 2013 | 1.37 | 0.25 | 3425 | 375 |
| BIOW | 2015 | 1.39 | 0.25 | 3475 | 325 |
| MSW | 1998 | 1.26 | 0.25 | 3150 | 650 |
| MSW | 2002 | 1.28 | 0.25 | 3200 | 600 |
| MSW | 2004 | 1.30 | 0.25 | 3250 | 550 |
| MSW | 2006 | 1.31 | 0.25 | 3275 | 525 |
| MSW | 2007 | 1.31 | 0.25 | 3275 | 525 |
| MSW | 2009 | 1.32 | 0.25 | 3300 | 500 |
| MSW | 2011 | 1.35 | 0.25 | 3375 | 425 |
| MSW | 2013 | 1.37 | 0.25 | 3425 | 375 |
| MSW | 2015 | 1.39 | 0.25 | 3475 | 325 |
| FYM | 1998 | 1.26 | 0.25 | 3150 | 650 |
| FYM | 2002 | 1.28 | 0.25 | 3200 | 600 |
| FYM | 2004 | 1.30 | 0.25 | 3250 | 550 |
| FYM | 2006 | 1.31 | 0.25 | 3275 | 525 |
| FYM | 2007 | 1.31 | 0.25 | 3275 | 525 |
| FYM | 2009 | 1.32 | 0.25 | 3300 | 500 |
| FYM | 2011 | 1.35 | 0.25 | 3375 | 425 |
| FYM | 2013 | 1.37 | 0.25 | 3425 | 375 |
| FYM | 2015 | 1.39 | 0.25 | 3475 | 325 |
| CN | 1998 | 1.27 | 0.25 | 3175 | 625 |
| CN | 2002 | 1.29 | 0.25 | 3225 | 575 |
| CN | 2004 | 1.32 | 0.25 | 3300 | 500 |
| CN | 2006 | 1.34 | 0.25 | 3350 | 450 |
| CN | 2007 | 1.36 | 0.25 | 3400 | 400 |
| CN | 2009 | 1.39 | 0.25 | 3475 | 325 |
| CN | 2011 | 1.43 | 0.25 | 3575 | 225 |
| CN | 2013 | 1.48 | 0.25 | 3700 | 100 |
| CN | 2015 | 1.52 | 0.25 | **3800*** | 0 |

** The soil mass in the topsoil layer of the control treatment in 2015 of 3800 t ha^-1^, i.e. W_top,CN,2015_, was used as the reference soil mass (W_top,i,n_). The differences between this reference mass and each soil topsoil layer mass were calculated for each treatment and year.*

# Appendix C: Trace element contents measured in the topsoil layer (0-25 cm) for the period 1998-2915

### Table C.1: Chemical properties and trace element contents in the topsoil layer over the period 1998-2015 ([TE]_top,i,n_)

With pH, organic carbon content (Org. C), cationic exchange capacity (CEC), and content of Cd, Cr, Cu, Hg, Ni, Pb and Zn; mean values ± standard deviation of the four replicates.

*Co-compost of sewage sludge and green waste (GWS), biowaste compost (BIOW), compost of residual municipal solid waste (MSW), farmyard manure (FYM), no organic amendment (CN).*

| **Soil** |  |  | **pH** | **Org. C** | **CEC** |  | **Cd** | **Cr** | **Cu** | **Hg** | **Ni** | **Pb** | **Zn** |
| --- | --- | --- | --- | --- | --- | --- | --- | --- | --- | --- | --- | --- | --- |
| **Treatment** | **Year** |  |  | g kg^-1^ DM | cmol+ kg^-1^ DM |  | mg kg^-1^ DM | mg kg^-1^ DM | mg kg^-1^ DM | mg kg^-1^ DM | mg kg^-1^ DM | mg kg^-1^ DM | mg kg^-1^ DM |
| GWS | 1998 |  | 7.0 ± 0.2 (a) | 10.6 ± 1.0 (a) | 9.4 ± 0.7 (b) |  | 0.24 ± 0.02 (a) | 44.8 ± 4.0 (a) | 11.8 ± 1.2 (a) | 0.10 ± 0.02 (a) | 14.6 ± 0.5 (a) | 30.0 ± 7.5 (a) | 51.8 ± 2.5 (a) |
| BIOW | 1998 |  | 7.2 ± 0.3 (a) | 10.8 ± 0.7 (a) | 9.8 ± 0.8 (ab) |  | 0.23 ± 0.01 (a) | 45.5 ± 3.4 (a) | 12.1 ± 1.1 (a) | 0.10 ± 0.02 (a) | 14.7 ± 0.8 (a) | 24.7 ± 0.8 (a) | 50.3 ± 1.2 (a) |
| MSW | 1998 |  | 7.0 ± 0.2 (a) | 10.4 ± 0.7 (a) | 9.3 ± 0.9 (b) |  | 0.24 ± 0.01 (a) | 46.8 ± 3.8 (a) | 11.7 ± 0.3 (a) | 0.10 ± 0.02 (a) | 14.6 ± 0.5 (a) | 25.2 ± 2.7 (a) | 53.2 ± 6.0 (a) |
| FYM | 1998 |  | 7.0 ± 0.1 (a) | 10.6 ± 0.3 (a) | 9.8 ± 0.8 (ab) |  | 0.24 ± 0.01 (a) | 44.5 ± 1.7 (a) | 12.6 ± 1.4 (a) | 0.10 ± 0.02 (a) | 14.8 ± 1.0 (a) | 25.1 ± 2.8 (a) | 51.7 ± 4.3 (a) |
| CN | 1998 |  | 7.1 ± 0 (a) | 10.7 ± 0.2 (a) | 10.1 ± 0.9 (a) |  | 0.24 ± 0.01 (a) | 46.7 ± 3.0 (a) | 12.0 ± 0.9 (a) | 0.09 ± 0.01 (a) | 15.1 ± 0.8 (a) | 23.7 ± 0.9 (a) | 50.9 ± 2.7 (a) |
| ***Treatment effect*** | |  | ***ns*** | ***ns*** | ***ns*** |  | ***ns*** | ***ns*** | ***ns*** | ***ns*** | ***ns*** | ***ns*** | ***ns*** |
| GWS | 2002 |  | 6.9 ± 0.1 (a) | 11.4 ± 1.2 (a) | 9.5 ± 0.7 (a) |  | 0.24 ± 0.01 (a) | 42.0 ± 2.8 (a) | 12.4 ± 1.4 (a) | 0.10 ± 0.02 (a) | 14.8 ± 0.5 (a) | 24.1 ± 2.4 (a) | 49.9 ± 0.8 (a) |
| BIOW | 2002 |  | 7.3 ± 0.1 (a) | 12.0 ± 0.7 (a) | 10.4 ± 0.4 (a) |  | 0.24 ± 0.01 (a) | 43.8 ± 1.7 (a) | 12.8 ± 0.9 (a) | 0.10 ± 0.03 (a) | 15.5 ± 0.6 (a) | 25.1 ± 1.0 (a) | 53.1 ± 1.0 (a) |
| MSW | 2002 |  | 7.4 ± 0.2 (a) | 11.1 ± 1.1 (a) | 10.0 ± 0.7 (a) |  | 0.24 ± 0.01 (a) | 43.8 ± 4.7 (a) | 13.1 ± 0.6 (a) | 0.11 ± 0.02 (a) | 15.1 ± 0.9 (a) | 26.1 ± 2.3 (a) | 53.5 ± 2.6 (a) |
| FYM | 2002 |  | 7.2 ± 0.1 (a) | 12.3 ± 1.3 (a) | 10.1 ± 0.7 (a) |  | 0.25 ± 0.02 (a) | 45.0 ± 3.2 (a) | 13.5 ± 1.6 (a) | 0.09 ± 0.03 (a) | 15.5 ± 1.0 (a) | 25.8 ± 2.2 (a) | 55.3 ± 3.8 (a) |
| CN | 2002 |  | 7.0 ± 0.1 (a) | 10.5 ± 0.6 (a) | 9.8 ± 0.5 (a) |  | 0.23 ± 0.01 (a) | 44.0 ± 2.2 (a) | 11.8 ± 1.0 (a) | 0.08 ± 0.01 (a) | 15.5 ± 0.9 (a) | 25.1 ± 1.9 (a) | 48.5 ± 1.5 (a) |
| ***Treatment effect*** | |  | ***ns*** | ***ns*** | ***ns*** |  | ***ns*** | ***ns*** | ***ns*** | ***ns*** | ***ns*** | ***ns*** | ***ns*** |
| GWS | 2004 |  | 6.8 ± 0.1 (c) | 12.3 ± 0.8 (a) | 9.0 ± 0.3 (b) |  | 0.24 ± 0.00 (a) | 44.6 ± 1.5 (a) | 13.7 ± 1.5 (a) | 0.10 ± 0.03 (b) | 14.6 ± 0.4 (a) | 29.0 ± 6.1 (a) | 52.4 ± 1.8 (ab) |
| BIOW | 2004 |  | 7.4 ± 0.1 (a) | 12.3 ± 0.5 (a) | 10.0 ± 0.6 (a) |  | 0.24 ± 0.02 (a) | 46.2 ± 0.8 (a) | 13.5 ± 1.2 (a) | 0.09 ± 0.02 (bc) | 15.3 ± 0.4 (a) | 27.5 ± 2.6 (a) | 55.1 ± 3.2 (ab) |
| MSW | 2004 |  | 7.4 ± 0.1 (a) | 11.3 ± 0.7 (b) | 9.7 ± 0.8 (a) |  | 0.24 ± 0.01 (a) | 45.6 ± 0.8 (a) | 14.0 ± 0.5 (a) | 0.12 ± 0.02 (a) | 14.9 ± 0.6 (a) | 28.7 ± 3.3 (a) | 54.1 ± 2.1 (ab) |
| FYM | 2004 |  | 7.2 ± 0.1 (b) | 12.1 ± 0.5 (a) | 9.9 ± 0.4 (a) |  | 0.24 ± 0.02 (a) | 45.5 ± 2.5 (a) | 14.1 ± 1.2 (a) | 0.09 ± 0.03 (bc) | 15.3 ± 0.9 (a) | 31.4 ± 9.7 (a) | 56.6 ± 3.9 (a) |
| CN | 2004 |  | 6.9 ± 0.2 (c) | 10.4 ± 0.2 (c) | 8.8 ± 0.9 (b) |  | 0.23 ± 0.02 (a) | 46.2 ± 1.4 (a) | 12.2 ± 1.1 (b) | 0.08 ± 0.02 (c) | 14.8 ± 0.8 (a) | 25.5 ± 2.0 (a) | 51.0 ± 2.1 (b) |
| ***Treatment effect*** | |  | ******** | ******** | ******** |  | ***ns*** | ***ns*** | ******* | ******** | ***ns*** | ***ns*** | ******* |
| GWS | 2006 |  | 6.8 ± 0.2 (d) | 12.6 ± 1.2 (a) | 8.8 ± 0.8 (bc) |  | 0.22 ± 0.01 (a) | 44.2 ± 0.9 (a) | 14.6 ± 1.1 (a) | 0.09 ± 0.02 (ab) | 15.2 ± 0.3 (a) | 27.4 ± 2.5 (a) | 54.5 ± 1.1 (a) |
| BIOW | 2006 |  | 7.6 ± 0.1 (a) | 12.2 ± 0.8 (a) | 10.0 ± 0.5 (a) |  | 0.23 ± 0.01 (a) | 45.7 ± 0.9 (a) | 13.8 ± 1.4 (a) | 0.08 ± 0.01 (bc) | 15.6 ± 0.5 (a) | 26.6 ± 0.9 (a) | 57.8 ± 3.3 (a) |
| MSW | 2006 |  | 7.4 ± 0.2 (b) | 11.6 ± 0.8 (a) | 9.2 ± 0.7 (bc) |  | 0.22 ± 0.00 (a) | 45.6 ± 0.9 (a) | 14.6 ± 0.3 (a) | 0.10 ± 0.02 (a) | 15.3 ± 0.6 (a) | 29.5 ± 7.1 (a) | 55.0 ± 2.2 (a) |
| FYM | 2006 |  | 7.2 ± 0.1 (c) | 12.6 ± 1.1 (a) | 9.5 ± 0.6 (ab) |  | 0.23 ± 0.01 (a) | 45.1 ± 1.0 (a) | 14.4 ± 1.3 (a) | 0.08 ± 0.03 (bc) | 15.3 ± 0.8 (a) | 27.1 ± 2.4 (a) | 56.2 ± 3.3 (a) |
| CN | 2006 |  | 6.9 ± 0.3 (d) | 9.9 ± 0.8 (b) | 8.6 ± 0.8 (c) |  | 0.22 ± 0.01 (a) | 47.9 ± 4.0 (a) | 12.3 ± 1.0 (b) | 0.07 ± 0.01 (c) | 15.5 ± 0.9 (a) | 23.9 ± 0.9 (a) | 50.6 ± 1.9 (b) |
| ***Treatment effect*** | |  | ******** | ******** | ******** |  | ***ns*** | ***ns*** | ******* | ******** | ***ns*** | ***ns*** | ******** |
| GWS | 2007 |  | 6.9 ± 0.1 (b) | 14.0 ± 0.4 (a) | 9.2 ± 0.5 (bc) |  | 0.23 ± 0.01 (a) | 44.0 ± 2.0 (a) | 15.5 ± 0.9 (a) | 0.11 ± 0.01 (a) | 14.8 ± 0.3 (a) | 24.0 ± 2.1 (ab) | 56.0 ± 2.4 (ab) |
| BIOW | 2007 |  | 7.7 ± 0.1 (a) | 14.3 ± 1.4 (a) | 10.3 ± 0.6 (a) |  | 0.23 ± 0.01 (a) | 45.9 ± 5.5 (a) | 14.1 ± 1.5 (ab) | 0.08 ± 0.02 (ab) | 15.3 ± 0.2 (a) | 27.1 ± 2.4 (a) | 59.6 ± 6.4 (a) |
| MSW | 2007 |  | 7.4 ± 0.1 (a) | 12.0 ± 0.2 (bc) | 9.3 ± 0.5 (abc) |  | 0.22 ± 0.01 (ab) | 45.9 ± 2.7 (a) | 14.8 ± 0.3 (a) | 0.12 ± 0.03 (a) | 15.2 ± 0.9 (a) | 27.7 ± 3.0 (a) | 56.0 ± 2.9 (ab) |
| FYM | 2007 |  | 7.2 ± 0.1 (ab) | 13.8 ± 0.9 (ab) | 9.8 ± 0.5 (ab) |  | 0.23 ± 0.01 (a) | 43.4 ± 1.3 (a) | 15.0 ± 1.7 (a) | 0.09 ± 0.03 (ab) | 15.0 ± 0.8 (a) | 25.2 ± 2.7 (ab) | 58.5 ± 3.6 (a) |
| CN | 2007 |  | 6.8 ± 0.1 (b) | 10.5 ± 0.3 (c) | 8.5 ± 0.8 (c) |  | 0.21 ± 0.00 (b) | 43.2 ± 1.0 (a) | 12.1 ± 0.6 (b) | 0.07 ± 0.02 (b) | 14.9 ± 0.7 (a) | 21.7 ± 0.9 (b) | 50.5 ± 3.8 (b) |
| ***Treatment effect*** | |  | ******** | ******** | ******* |  | ***ns*** | ***ns*** | ******* | ***ns*** | ***ns*** | ******* | ***ns*** |
| GWS | 2009 |  | 6.9 ± 0 (ab) | 14.8 ± 0.8 (b) | 9.8 ± 0.5 (ab) |  | 0.24 ± 0.01 (ab) | 65.6 ± 1.9 (a) | 15.8 ± 0.8 (b) | 0.11 ± 0.02 (a) | 15.0 ± 0.4 (a) | 25.8 ± 2.0 (ab) | 59.0 ± 2.0 (ab) |
| BIOW | 2009 |  | 7.7 ± 0 (c) | 14.8 ± 0.6 (b) | 10.9 ± 0.4 (b) |  | 0.24 ± 0.01 (ab) | 64.4 ± 4.1 (a) | 14.2 ± 1.2 (ab) | 0.08 ± 0.02 (a) | 15.4 ± 0.7 (a) | 28.7 ± 5.2 (ab) | 58.0 ± 2.0 (ab) |
| MSW | 2009 |  | 7.5 ± 0.1 (bc) | 12.4 ± 0.3 (ab) | 9.7 ± 0.4 (ab) |  | 0.23 ± 0.01 (ab) | 67.8 ± 4.4 (a) | 14.9 ± 0.3 (ab) | 0.10 ± 0.02 (a) | 15.2 ± 0.4 (a) | 27.4 ± 2.6 (ab) | 57.8 ± 0.9 (ab) |
| FYM | 2009 |  | 7.3 ± 0 (abc) | 14.3 ± 0.4 (ab) | 10.1 ± 0.2 (ab) |  | 0.25 ± 0.01 (b) | 66.3 ± 6.3 (a) | 15.2 ± 1.5 (ab) | 0.10 ± 0.04 (a) | 15.1 ± 1.0 (a) | 31.7 ± 2.4 (b) | 60.0 ± 2.8 (b) |
| CN | 2009 |  | 6.8 ± 0.1 (a) | 10.6 ± 0.3 (a) | 8.6 ± 0.7 (a) |  | 0.22 ± 0.01 (a) | 71.5 ± 2.7 (a) | 12.0 ± 0.9 (a) | 0.07 ± 0.02 (a) | 15.1 ± 0.6 (a) | 22.9 ± 1.0 (a) | 50.1 ± 1.8 (a) |
| ***Treatment effect*** | |  | ******** | ******** | ******** |  | ******* | ***ns*** | ******* | ***ns*** | ***ns*** | ******* | ******* |
| GWS | 2011 |  | 6.9 ± 0.0 (d) | 15.6 ± 0.3 (a) | 10.1 ± 0.6 (b) |  | 0.23 ± 0.01 (a) | 66.3 ± 5.7 (a) | 17.4 ± 0.9 (a) | 0.10 ± 0.02 (a) | 15.3 ± 0.7 (a) | 24.3 ± 1.9 (a) | 62.0 ± 1.3 (a) |
| BIOW | 2011 |  | 7.8 ± 0.0 (a) | 15.2 ± 0.5 (a) | 11.3 ± 0.6 (a) |  | 0.23 ± 0.00 (a) | 66.7 ± 5.8 (a) | 14.7 ± 0.8 (b) | 0.07 ± 0.02 (ab) | 15.7 ± 0.9 (a) | 24.6 ± 0.2 (a) | 59.8 ± 3.4 (a) |
| MSW | 2011 |  | 7.5 ± 0.1 (b) | 12.8 ± 0.6 (c) | 9.9 ± 0.8 (b) |  | 0.22 ± 0.01 (a) | 64.1 ± 3.1 (a) | 15.6 ± 0.8 (b) | 0.10 ± 0.03 (a) | 15.2 ± 0.3 (a) | 24.8 ± 2.3 (a) | 60.7 ± 5.3 (a) |
| FYM | 2011 |  | 7.3 ± 0.1 (c) | 14.4 ± 0.5 (b) | 10.2 ± 0.6 (b) |  | 0.23 ± 0.01 (a) | 64.5 ± 5.1 (a) | 15.4 ± 0.7 (b) | 0.07 ± 0.02 (ab) | 15.5 ± 0.8 (a) | 24.1 ± 1.7 (a) | 58.8 ± 2.0 (a) |
| CN | 2011 |  | 6.7 ± 0.1 (e) | 10.4 ± 0.3 (d) | 8.4 ± 1.0 (c) |  | 0.21 ± 0.01 (b) | 71.3 ± 1.1 (a) | 12.2 ± 1.1 (c) | 0.06 ± 0.01 (b) | 15.2 ± 0.9 (a) | 22.0 ± 1.3 (a) | 49.4 ± 1.0 (b) |
| ***Treatment effect*** | |  | ******** | ******** | ******** |  | ******** | ***ns*** | ******** | ******** | ***ns*** | ***ns*** | ******** |
| GWS | 2013 |  | 6.8 ± 0.1 (d) | 15.7 ± 0.2 (b) | 9.8 ± 0.6 (ab) |  | 0.24 ± 0.00 (a) | 70.4 ± 6.2 (a) | 18.9 ± 1.1 (a) | 0.11 ± 0.02 (a) | 15.5 ± 0.2 (a) | 25.2 ± 2.6 (ab) | 64.5 ± 1.6 (a) |
| BIOW | 2013 |  | 7.6 ± 0.0 (a) | 16.1 ± 0.4 (a) | 10.7 ± 0.5 (b) |  | 0.23 ± 0.01 (a) | 68.9 ± 7.5 (a) | 15.9 ± 1.1 (b) | 0.08 ± 0.02 (a) | 15.9 ± 0.5 (a) | 27.9 ± 3.2 (a) | 61.4 ± 2.0 (b) |
| MSW | 2013 |  | 7.4 ± 0.1 (b) | 13.2 ± 0.4 (d) | 9.8 ± 0.8 (ab) |  | 0.23 ± 0.01 (a) | 71.8 ± 5.4 (a) | 16.2 ± 0.6 (b) | 0.11 ± 0.04 (a) | 15.7 ± 0.7 (a) | 26.3 ± 2.6 (ab) | 59.9 ± 2.6 (b) |
| FYM | 2013 |  | 7.2 ± 0.1 (c) | 14.9 ± 0.4 (c) | 9.9 ± 0.6 (ab) |  | 0.23 ± 0.01 (a) | 72.1 ± 1.3 (a) | 15.9 ± 1.2 (b) | 0.08 ± 0.02 (a) | 15.5 ± 0.8 (a) | 25.8 ± 1.6 (ab) | 60.4 ± 4.1 (b) |
| CN | 2013 |  | 6.6 ± 0.2 (e) | 10.5 ± 0.2 (e) | 7.9 ± 0.8 (a) |  | 0.21 ± 0.01 (b) | 74.6 ± 4.8 (a) | 12.9 ± 0.7 (c) | 0.07 ± 0.01 (a) | 15.5 ± 0.6 (a) | 22.7 ± 1.7 (b) | 50.6 ± 1.2 (c) |
| ***Treatment effect*** | |  | ******** | ******** | ******* |  | ******** | ***ns*** | ******** | ******* | ***ns*** | ******* | ******** |
| GWS | 2015 |  | 7.0 ± 0.0 (c) | 16.9 ± 0.9 (a) | 10.7 ± 0.4 (b) |  | 0.25 ± 0.01 (ab) | 73.4 ± 10.2 (ab) | 19.9 ± 1.1 (a) | 0.12 ± 0.02 (a) | 15.6 ± 0.2 (a) | 27.3 ± 5.5 (a) | 68.2 ± 2.2 (b) |
| BIOW | 2015 |  | 7.8 ± 0.1 (a) | 16.5 ± 0.3 (a) | 12.0 ± 0.4 (a) |  | 0.25 ± 0.04 (b) | 75.1 ± 7.5 (b) | 15.0 ± 0.9 (b) | 0.08 ± 0.01 (bc) | 15.9 ± 0.8 (a) | 26.1 ± 2.1 (a) | 60.5 ± 1.1 (ab) |
| MSW | 2015 |  | 7.8 ± 0.1 (a) | 13.2 ± 0.2 (c) | 10.5 ± 0.5 (b) |  | 0.23 ± 0.01 (ab) | 74.3 ± 4.7 (ab) | 16.2 ± 0.6 (b) | 0.10 ± 0.02 (bc) | 15.5 ± 0.6 (a) | 27.7 ± 4.1 (a) | 61.3 ± 2.7 (ab) |
| FYM | 2015 |  | 7.4 ± 0.0 (b) | 14.8 ± 0.4 (b) | 10.5 ± 0.4 (b) |  | 0.24 ± 0.01 (ab) | 74.1 ± 3.1 (ab) | 15.5 ± 1.1 (b) | 0.08 ± 0.02 (bc) | 15.5 ± 0.8 (a) | 25.3 ± 0.9 (a) | 61.1 ± 5.3 (ab) |
| CN | 2015 |  | 6.8 ± 0.2 (d) | 10.0 ± 0.3 (d) | 8.5 ± 0.6 (c) |  | 0.20 ± 0.02 (a) | 72.5 ± 8.0 (a) | 12.0 ± 0.8 (c) | 0.07 ± 0.01 (c) | 15.3 ± 0.9 (a) | 21.3 ± 0.9 (b) | 49.3 ± 2.1 (a) |
| ***Treatment effect*** | |  | ******** | ******* | ******* |  | ******* | ******** | ******** | ******** | ***ns*** | ******** | ******** |

ANOVA and Kruskal-Wallis tests were performed to test the treatment effect: ns stand for no significant difference while * and ** indicate significant difference at the probability level of *P<0.05* and *P<0.01* respectively. Abc letters stand for significant difference between treatments for each year, according to Newman-Keuls’s parametric and Bilateral Dunn’s non-parametric tests.

### Table C.2: Temporal statistics for the chemical properties and trace element contents in the topsoil layer over the period 1998-2015 ([TE]_top,i,n_)

*Co-compost of sewage sludge and green waste (GWS), biowaste compost (BIOW), compost of residual municipal solid waste (MSW), farmyard manure (FYM), no organic amendment (CN).*

| **Treatment** | **Year** |  | **pHeau** | **Org. C** | **Total N** | **CEC** |  | **Cd** | **Cu** | **Hg** | **Ni** | **Pb** | **Zn** |
| --- | --- | --- | --- | --- | --- | --- | --- | --- | --- | --- | --- | --- | --- |
| GWS | 1998 |  | ab | c | b | ab |  | ab | c | a | a | a | bc |
| GWS | 2002 |  | ab | bc | b | ab |  | ab | bc | a | a | a | c |
| GWS | 2004 |  | ab | abc | b | b |  | ab | abc | a | a | a | abc |
| GWS | 2007 |  | ab | abc | ab | b |  | b | abc | a | a | a | abc |
| GWS | 2009 |  | ab | abc | ab | ab |  | ab | abc | a | a | a | abc |
| GWS | 2011 |  | ab | abc | ab | ab |  | ab | abc | a | a | a | abc |
| GWS | 2013 |  | b | ab | ab | ab |  | ab | ab | a | a | a | ab |
| GWS | 2015 |  | a | a | a | a |  | a | a | a | a | a | a |
| BIOW | 1998 |  | b | b | c | b |  | a | c | a | b | a | b |
| BIOW | 2002 |  | b | ab | bc | ab |  | a | bc | a | ab | a | ab |
| BIOW | 2004 |  | ab | ab | bc | b |  | a | abc | ab | ab | a | ab |
| BIOW | 2007 |  | ab | ab | abc | ab |  | a | abc | ab | ab | a | ab |
| BIOW | 2009 |  | ab | ab | abc | ab |  | a | abc | ab | ab | a | ab |
| BIOW | 2011 |  | a | ab | abc | ab |  | a | abc | b | ab | a | ab |
| BIOW | 2013 |  | ab | a | ab | ab |  | a | a | ab | a | a | a |
| BIOW | 2015 |  | a | a | a | a |  | a | ab | ab | ab | a | a |
| MSW | 1998 |  | b | b | ab | ab |  | ab | b | a | b | ab | b |
| MSW | 2002 |  | ab | ab | ab | ab |  | a | ab | a | ab | ab | b |
| MSW | 2004 |  | ab | ab | b | ab |  | ab | ab | a | ab | a | ab |
| MSW | 2007 |  | ab | ab | ab | b |  | ab | ab | a | ab | ab | ab |
| MSW | 2009 |  | ab | ab | ab | ab |  | ab | ab | a | ab | ab | ab |
| MSW | 2011 |  | a | ab | ab | ab |  | b | ab | a | ab | b | ab |
| MSW | 2013 |  | ab | a | a | ab |  | ab | a | a | a | ab | ab |
| MSW | 2015 |  | a | a | a | a |  | ab | a | a | ab | ab | a |
| FYM | 1998 |  | b | b | b | a |  | a | b | a | a | ab | a |
| FYM | 2002 |  | ab | ab | ab | a |  | a | ab | ab | a | ab | a |
| FYM | 2004 |  | ab | ab | b | a |  | a | ab | ab | a | ab | a |
| FYM | 2007 |  | ab | ab | ab | a |  | a | ab | ab | a | ab | a |
| FYM | 2009 |  | ab | ab | ab | a |  | a | ab | ab | a | a | a |
| FYM | 2011 |  | ab | ab | ab | a |  | a | ab | b | a | b | a |
| FYM | 2013 |  | ab | a | a | a |  | a | a | ab | a | ab | a |
| FYM | 2015 |  | a | a | a | a |  | a | a | ab | a | ab | a |
| CN | 1998 |  | a | a | a | a |  | a | ab | a | a | ab | a |
| CN | 2002 |  | ab | ab | ab | a |  | ab | b | ab | a | ab | a |
| CN | 2004 |  | ab | ab | abc | ab |  | ab | ab | ab | a | a | a |
| CN | 2007 |  | abc | ab | abc | ab |  | ab | ab | ab | a | ab | a |
| CN | 2009 |  | abc | ab | abc | ab |  | ab | ab | ab | a | ab | a |
| CN | 2011 |  | bc | ab | c | ab |  | b | ab | b | a | ab | a |
| CN | 2013 |  | c | ab | abc | b |  | ab | a | ab | a | ab | a |
| CN | 2015 |  | abc | b | bc | ab |  | ab | ab | ab | a | b | a |

Friedman’s non-parametric test was performed to test effect of years per treatment; Abc letters stand for significant difference between years per treatment, according to Bilateral Nemenyi’s non-parametric method.

# Appendix D: Trace element contents measured in the soil sub-layer (25-35 cm) and estimated values for the period 1998-2015

### Table D.1: Chemical properties and trace element contents in the soil sub-layer measured in 1998, 2010 and 2015 ([TE]_sub,i,n_)

With pH, organic carbon content (Org. C), cationic exchange capacity (CEC), and content of Cd, Cr, Cu, Hg, Ni, Pb and Zn; samples were sieved at 2 mm; mean values ± standard deviation of the four replicates, with 1998 (no replicates), 2010 and 2015 (4 replicates); na stands for not available.

*Co-compost of sewage sludge and green waste (GWS), biowaste compost (BIOW), compost of residual municipal solid waste (MSW), farmyard manure (FYM), no organic amendment (CN).*

|  |  |  | **pH** | **Org. C** | **CEC** |  | **Cd** | **Cr** | **Cu** | **Hg** | **Ni** | **Pb** | **Zn** |
| --- | --- | --- | --- | --- | --- | --- | --- | --- | --- | --- | --- | --- | --- |
| **Treatment** | **Year** |  |  | g kg^-1^ DM | cmol+ kg^-1^ DM |  | mg kg^-1^ DM | mg kg^-1^ DM | mg kg^-1^ DM | mg kg^-1^ DM | mg kg^-1^ DM | mg kg^-1^ DM | mg kg^-1^ DM |
| GWS | 1998 |  | 7.4 | 3.6 | na |  | 0.10 | 52.3 | 9.5 | 0.03 | 21.4 | 18.3 | 52.3 |
| GWS | 2010 |  | 7.0 ± 0.1 (bc) | 12.0 ± 1.2 (a) | na |  | 0.22 ± 0.01 (a) | 71.6 ± 4.3 (a) | 14.3 ± 1.3 (a) | 0.11 ± 0.03 (a) | 14.8 ± 0.5 (a) | 23.2 ± 1.7 (a) | 54.0 ± 2.5 (a) |
| GWS | 2015 |  | 7.2 ± 0.2 (bc) | 13.6 ± 2.1 (a) | 10.1 ± 0.7 (ab) |  | 0.23 ± 0.02 (a) | 75.2 ± 2.5 (a) | 17.0 ± 2.2 (a) | 0.10 ± 0.01 (a) | 15.6 ± 0.5 (a) | 25.0 ± 2.4 (a) | 60.8 ± 4.5 (a) |
| BIOW | 1998 |  | 7.4 | 3.6 | na |  | 0.10 | 52.3 | 9.5 | 0.03 | 21.4 | 18.3 | 52.3 |
| BIOW | 2010 |  | 7.8 ± 0.1 (a) | 11.9 ± 1.2 (a) | na |  | 0.23 ± 0.01 (a) | 67.7 ± 6.7 (ab) | 13.6 ± 1.1 (ab) | 0.09 ± 0.02 (ab) | 15.3 ± 0.7 (a) | 24.8 ± 1.6 (a) | 54.3 ± 3.2 (a) |
| BIOW | 2015 |  | 7.9 ± 0.0 (a) | 13.5 ± 1.1 (a) | 10.9 ± 0.5 (a) |  | 0.23 ± 0.01 (a) | 72.8 ± 4 (ab) | 14.6 ± 1.3 (ab) | 0.08 ± 0.02 (ab) | 16.2 ± 0.8 (a) | 25.9 ± 3.6 (a) | 58.5 ± 2.2 (a) |
| MSW | 1998 |  | 7.4 | 3.6 | na |  | 0.10 | 52.3 | 9.5 | 0.03 | 21.4 | 18.3 | 52.3 |
| MSW | 2010 |  | 7.6 ± 0.1 (ab) | 10.7 ± 0.6 (bc) | na |  | 0.22 ± 0.00 (a) | 68.7 ± 5.8 (b) | 14.4 ± 0.4 (ab) | 0.10 ± 0.02 (ab) | 15.1 ± 0.5 (a) | 24.6 ± 2.2 (a) | 55.3 ± 2.0 (ab) |
| MSW | 2015 |  | 7.8 ± 0.1 (ab) | 10.1 ± 1.0 (bc) | 9.6 ± 0.4 (bc) |  | 0.21 ± 0.02 (a) | 71.4 ± 0.9 (b) | 14.5 ± 1.2 (ab) | 0.09 ± 0.02 (ab) | 15.7 ± 0.6 (a) | 25.4 ± 3.5 (a) | 55.6 ± 2.7 (ab) |
| FYM | 1998 |  | 7.4 | 3.6 | na |  | 0.10 | 52.3 | 9.5 | 0.03 | 21.4 | 18.3 | 52.3 |
| FYM | 2010 |  | 7.5 ± 0.0 (abc) | 12.0 ± 0.4 (ab) | na |  | 0.23 ± 0.01 (a) | 69.2 ± 8.0 (a) | 14.5 ± 1.5 (ab) | 0.09 ± 0.03 (ab) | 15.1 ± 1.2 (a) | 23.9 ± 2.3 (ab) | 54.9 ± 2.9 (a) |
| FYM | 2015 |  | 7.5 ± 0.1 (abc) | 11.7 ± 1.8 (ab) | 9.8 ± 0.6 (abc) |  | 0.22 ± 0.02 (a) | 75.7 ± 3.6 (a) | 14.9 ± 1.2 (ab) | 0.08 ± 0.03 (ab) | 16.0 ± 1.1 (a) | 23.9 ± 2.7 (ab) | 58.2 ± 3.2 (a) |
| CN | 1998 |  | 7.4 | 3.6 | na |  | 0.10 | 52.3 | 9.5 | 0.03 | 21.4 | 18.3 | 52.3 |
| CN | 2010 |  | 7.0 ± 0.2 (c) | 9.4 ± 0.6 (c) | na |  | 0.21 ± 0.01 (a) | 71.6 ± 5.6 (ab) | 12.0 ± 0.9 (b) | 0.08 ± 0.01 (b_ẟ) | 15.3 ± 1.2 (a) | 21.7 ± 1.6 (b_ẟ) | 48.4 ± 1.8 (b) |
| CN | 2015 |  | 6.9 ± 0.1 (c) | 8.6 ± 1.3 (c) | 8.7 ± 0.7 (c) |  | 0.20 ± 0.03 (a) | 73.6 ± 2.9 (ab) | 13.2 ± 3.4 (b) | 0.06 ± 0.01 (b_ẟ) | 16.0 ± 0.7 (a) | 20.7 ± 0.7 (b_ẟ) | 50.5 ± 2.8 (b) |
|  |  |  |  |  |  |  |  |  |  |  |  |  |  |
| ***Treatment effect*** | 2010 |  | ** | ** | * |  | ns | ns | ns | ns | ns | ns | * |
|  | 2015 |  | ** | ** | * |  | ns | ns | ns | ns | ns | ns | * |

Kruskal-Wallis’s non-parametric test was performed to identify the treatment effect: ns stand for no significant difference while * and ** indicate significant difference at the probability level of *P<0.05* and *P<0.01* respectively.

Bilateral Dunn’s non-parametric test was performed to identify significant differences between treatments (Abc letters stand for significant difference between treatments).

Friedman’s non-parametric test was performed to evaluate putative significant differences between samples for the control treatment in 2010 and 2015, ẟ stands for significant difference between years per treatment, according to Bilateral Nemenyi’s non-parametric method.

### Tables D.2: Linear regression equations used to estimate the trace element contents in the sub-layer for each treatment per year ([TE]_sub,i,n_)

*Co-compost of sewage sludge and green waste (GWS), biowaste compost (BIOW), compost of residual municipal solid waste (MSW), farmyard manure (FYM), no organic amendment (CN).*

| **[TE]_sub,i,2015_ as references** | **x** | **0*** | **17**** |  | **a** | **b** |
| --- | --- | --- | --- | --- | --- | --- |
| Cd | GWS | 0.20 | 0.23 |  | 0.0016 | 0.20 |
| Cd | BIOW | 0.20 | 0.23 |  | 0.0018 | 0.20 |
| Cd | MSW | 0.20 | 0.21 |  | 0.0005 | 0.20 |
| Cd | FYM | 0.20 | 0.22 |  | 0.0011 | 0.20 |
| Cd | CN | 0.20 | 0.20 |  | 0 | 0.20 |
| Cr | GWS | 73.60 | 75.23 |  | 0.0963 | 73.60 |
| Cr | BIOW | 73.60 | 72.75 |  | -0.0497 | 73.60 |
| Cr | MSW | 73.60 | 71.38 |  | -0.1302 | 73.60 |
| Cr | FYM | 73.60 | 75.74 |  | 0.1261 | 73.60 |
| Cr | CN | 73.60 | 73.60 |  | 0 | 73.60 |
| Cu | GWS | 13.24 | 17.04 |  | 0.2236 | 13.24 |
| Cu | BIOW | 13.24 | 14.61 |  | 0.0807 | 13.24 |
| Cu | MSW | 13.24 | 14.45 |  | 0.0714 | 13.24 |
| Cu | FYM | 13.24 | 14.86 |  | 0.095 | 13.24 |
| Cu | CN | 13.24 | 13.24 |  | 0 | 13.24 |
| Hg | GWS | 0.06 | 0.10 |  | 0.0023 | 0.06 |
| Hg | BIOW | 0.06 | 0.08 |  | 0.001 | 0.06 |
| Hg | MSW | 0.06 | 0.09 |  | 0.0017 | 0.06 |
| Hg | FYM | 0.06 | 0.08 |  | 0.0011 | 0.06 |
| Hg | CN | 0.06 | 0.06 |  | 0 | 0.06 |
| Ni | GWS | 16.02 | 15.60 |  | -0.0247 | 16.02 |
| Ni | BIOW | 16.02 | 16.15 |  | 0.0079 | 16.02 |
| Ni | MSW | 16.02 | 15.72 |  | -0.0177 | 16.02 |
| Ni | FYM | 16.02 | 15.97 |  | -0.003 | 16.02 |
| Ni | CN | 16.02 | 16.02 |  | 0 | 16.02 |
| Pb | GWS | 20.72 | 25.03 |  | 0.2536 | 20.72 |
| Pb | BIOW | 20.72 | 25.93 |  | 0.3069 | 20.72 |
| Pb | MSW | 20.72 | 25.37 |  | 0.2736 | 20.72 |
| Pb | FYM | 20.72 | 23.92 |  | 0.1886 | 20.72 |
| Pb | CN | 20.72 | 20.72 |  | 0 | 20.72 |
| Zn | GWS | 50.46 | 60.75 |  | 0.6057 | 50.46 |
| Zn | BIOW | 50.46 | 58.52 |  | 0.4743 | 50.46 |
| Zn | MSW | 50.46 | 55.59 |  | 0.3021 | 50.46 |
| Zn | FYM | 50.46 | 58.23 |  | 0.4576 | 50.46 |
| Zn | CN | 50.46 | 50.46 |  | 0 | 50.46 |

** Trace element contents measured in the sub-layer for the control treatment in 2015, i.e. [TE]_sub,CN,2015_, as reference for [TE]_sub,CN,1998_ (year 0, or 1998).*

*** Trace element contents measured in the sub-layer for the OWP treatments in 2015, i.e. [TE]_sub,OWP,2015_, as reference for high values in the regression (year 17, or 2015).*

### Tables D.3: Estimated values of the trace element contents in the sub-layer for each treatment per year ([TE]_sub,i,n_)

Considering the linear equations cited in Table D.2.

*Co-compost of sewage sludge and green waste (GWS), biowaste compost (BIOW), compost of residual municipal solid waste (MSW), farmyard manure (FYM), no organic amendment (CN).*

|  |  | **Cd** | **Cr** | **Cu** | **Hg** | **Ni** | **Pb** | **Zn** |
| --- | --- | --- | --- | --- | --- | --- | --- | --- |
| **Treatment** | **Year** | mg kg^-1^ DM | mg kg^-1^ DM | mg kg^-1^ DM | mg kg^-1^ DM | mg kg^-1^ DM | mg kg^-1^ DM | mg kg^-1^ DM |
| GWS | 1998 | 0.20 | 73.60 | 13.24 | 0.06 | 16.02 | 20.72 | 50.46 |
| GWS | 2002 | 0.21 | 73.98 | 14.13 | 0.07 | 15.92 | 21.73 | 52.88 |
| GWS | 2004 | 0.21 | 74.17 | 14.58 | 0.08 | 15.87 | 22.24 | 54.09 |
| GWS | 2006 | 0.22 | 74.37 | 15.03 | 0.08 | 15.82 | 22.75 | 55.30 |
| GWS | 2007 | 0.22 | 74.46 | 15.25 | 0.08 | 15.80 | 23.00 | 55.91 |
| GWS | 2009 | 0.22 | 74.66 | 15.70 | 0.09 | 15.75 | 23.51 | 57.12 |
| GWS | 2010 | 0.22 | 74.85 | 16.15 | 0.09 | 15.70 | 24.01 | 58.33 |
| GWS | 2013 | 0.23 | 75.04 | 16.59 | 0.10 | 15.65 | 24.52 | 59.54 |
| GWS | 2015 | 0.23 | 75.23 | 17.04 | 0.10 | 15.60 | 25.03 | 60.75 |
| BIOW | 1998 | 0.20 | 73.60 | 13.24 | 0.06 | 16.02 | 20.72 | 50.46 |
| BIOW | 2002 | 0.21 | 73.40 | 13.56 | 0.07 | 16.05 | 21.94 | 52.35 |
| BIOW | 2004 | 0.21 | 73.30 | 13.72 | 0.07 | 16.07 | 22.56 | 53.30 |
| BIOW | 2006 | 0.22 | 73.20 | 13.89 | 0.07 | 16.08 | 23.17 | 54.25 |
| BIOW | 2007 | 0.22 | 73.15 | 13.97 | 0.07 | 16.09 | 23.48 | 54.72 |
| BIOW | 2009 | 0.22 | 73.05 | 14.13 | 0.07 | 16.10 | 24.09 | 55.67 |
| BIOW | 2010 | 0.23 | 72.95 | 14.29 | 0.08 | 16.12 | 24.71 | 56.62 |
| BIOW | 2013 | 0.23 | 72.85 | 14.45 | 0.08 | 16.14 | 25.32 | 57.57 |
| BIOW | 2015 | 0.23 | 72.75 | 14.61 | 0.08 | 16.15 | 25.93 | 58.52 |
| MSW | 1998 | 0.20 | 73.60 | 13.24 | 0.06 | 16.02 | 20.72 | 50.46 |
| MSW | 2002 | 0.20 | 73.08 | 13.53 | 0.07 | 15.95 | 21.81 | 51.66 |
| MSW | 2004 | 0.21 | 72.82 | 13.67 | 0.07 | 15.91 | 22.36 | 52.27 |
| MSW | 2006 | 0.21 | 72.56 | 13.81 | 0.08 | 15.88 | 22.91 | 52.87 |
| MSW | 2007 | 0.21 | 72.43 | 13.88 | 0.08 | 15.86 | 23.18 | 53.17 |
| MSW | 2009 | 0.21 | 72.16 | 14.03 | 0.08 | 15.82 | 23.73 | 53.78 |
| MSW | 2010 | 0.21 | 71.90 | 14.17 | 0.09 | 15.79 | 24.27 | 54.38 |
| MSW | 2013 | 0.21 | 71.64 | 14.31 | 0.09 | 15.75 | 24.82 | 54.99 |
| MSW | 2015 | 0.21 | 71.38 | 14.45 | 0.09 | 15.72 | 25.37 | 55.59 |
| FYM | 1998 | 0.20 | 73.60 | 13.24 | 0.06 | 16.02 | 20.72 | 50.46 |
| FYM | 2002 | 0.21 | 74.10 | 13.62 | 0.07 | 16.01 | 21.47 | 52.29 |
| FYM | 2004 | 0.21 | 74.35 | 13.81 | 0.07 | 16.00 | 21.85 | 53.20 |
| FYM | 2006 | 0.21 | 74.61 | 14.00 | 0.07 | 15.99 | 22.23 | 54.12 |
| FYM | 2007 | 0.21 | 74.73 | 14.10 | 0.07 | 15.99 | 22.41 | 54.57 |
| FYM | 2009 | 0.21 | 74.98 | 14.29 | 0.08 | 15.99 | 22.79 | 55.49 |
| FYM | 2010 | 0.22 | 75.24 | 14.48 | 0.08 | 15.98 | 23.17 | 56.40 |
| FYM | 2013 | 0.22 | 75.49 | 14.67 | 0.08 | 15.97 | 23.55 | 57.32 |
| FYM | 2015 | 0.22 | 75.74 | 14.86 | 0.08 | 15.97 | 23.92 | 58.23 |
| CN | 1998 | 0.20 | 73.60 | 13.24 | 0.06 | 16.02 | 20.72 | 50.46 |
| CN | 2002 | 0.20 | 73.60 | 13.24 | 0.06 | 16.02 | 20.72 | 50.46 |
| CN | 2004 | 0.20 | 73.60 | 13.24 | 0.06 | 16.02 | 20.72 | 50.46 |
| CN | 2006 | 0.20 | 73.60 | 13.24 | 0.06 | 16.02 | 20.72 | 50.46 |
| CN | 2007 | 0.20 | 73.60 | 13.24 | 0.06 | 16.02 | 20.72 | 50.46 |
| CN | 2009 | 0.20 | 73.60 | 13.24 | 0.06 | 16.02 | 20.72 | 50.46 |
| CN | 2010 | 0.20 | 73.60 | 13.24 | 0.06 | 16.02 | 20.72 | 50.46 |
| CN | 2013 | 0.20 | 73.60 | 13.24 | 0.06 | 16.02 | 20.72 | 50.46 |
| CN | 2015 | 0.20 | 73.60 | 13.24 | 0.06 | 16.02 | 20.72 | 50.46 |

### Tables D.4: Percentage of accuracy between the estimated values calculated by the linear equations and the measured data

*Co-compost of sewage sludge and green waste (GWS), biowaste compost (BIOW), compost of residual municipal solid waste (MSW), farmyard manure (FYM), no organic amendment (CN).*

|  |  | **Cd** | **Cr** | **Cu** | **Hg** | **Ni** | **Pb** | **Zn** |
| --- | --- | --- | --- | --- | --- | --- | --- | --- |
| Treatment | Year | mg kg^-1^ DM | mg kg^-1^ DM | mg kg^-1^ DM | mg kg^-1^ DM | mg kg^-1^ DM | mg kg^-1^ DM | mg kg^-1^ DM |
| GWS | 1998 | 206 | 141 | 139 | 213 | 75 | 113 | **97** |
| GWS | 2010 | **99** | **104** | 113 | 83 | 106 | **103** | 108 |
| GWS | 2015 | 100 | 100 | 100 | 100 | 100 | 100 | 100 |
|  |  |  |  |  |  |  |  |  |
| BIOW | 1998 | 206 | 141 | 139 | 213 | 75 | 113 | **97** |
| BIOW | 2010 | **100** | 108 | **105** | 87 | **105** | **99** | **104** |
| BIOW | 2015 | 100 | 100 | 100 | 99 | 100 | 100 | 100 |
|  |  |  |  |  |  |  |  |  |
| MSW | 1998 | 206 | 141 | 139 | 213 | 75 | 113 | **97** |
| MSW | 2010 | 94 | **105** | **99** | 84 | **104** | **99** | **98** |
| MSW | 2015 | 100 | 100 | 100 | 100 | 100 | 100 | 100 |
|  |  |  |  |  |  |  |  |  |
| FYM | 1998 | 206 | 141 | 139 | 213 | 75 | 113 | **97** |
| FYM | 2010 | 93 | 109 | **100** | 89 | 106 | **97** | **103** |
| FYM | 2015 | 100 | 100 | 100 | 101 | 100 | 100 | 100 |
|  |  |  |  |  |  |  |  |  |
| CN | 1998 | 206 | 141 | 139 | 213 | 75 | 113 | **97** |
| CN | 2010 | **98** | **103** | 111 | 85 | **105** | **96** | **104** |
| CN | 2015 | 100 | 100 | 100 | 100 | 100 | 100 | 100 |

|  | **total 2010/2015** | **Cd** | **Cr** | **Cu** | **Hg** | **Ni** | **Pb** | **Zn** | **Percentage** |
| --- | --- | --- | --- | --- | --- | --- | --- | --- | --- |
| > 105 | 7 | 0 | 2 | 2 | 0 | 2 | 0 | 1 | 10 |
| < 95 | 7 | 2 | 0 | 0 | 5 | 0 | 0 | 0 | 10 |
| 95 - 105 | **56** | 8 | 8 | 8 | 5 | 8 | 10 | 9 | **80*** |
| > 101 | 18 | 0 | 5 | 3 | 0 | 5 | 1 | 4 | 26 |
| < 99 | 11 | 3 | 0 | 0 | 5 | 0 | 2 | 1 | 16 |
| 99 - 101 | **41** | 7 | 5 | 7 | 5 | 5 | 7 | 5 | **59**** |
| Total | 70 | 10 | 10 | 10 | 10 | 10 | 10 | 10 | **100** |

** Percentage of the estimated values comprised in an interval of 95%-105% compared to the data measured in 2010 and 2015:*

*- 80%, with [TE]_sub,CN,2015_ as reference for the lacking [TE]_sub,i,1998_, and the [TE]_sub,OWP,2015_ measured in the OWP treatments in 2015 for the final TE contents in the linear equations.*

*- 73%, with [TE]_sub,CN,2010_ as reference for the lacking [TE]_sub,i,1998_, and the [TE]_sub,OWP,2010_ measured in the OWP treatments in 2010 for the final TE contents in the linear equations.*

*- 87%, with an average content [TE]_sub,CN,2010/2015_ as the lacking [TE]_sub,i,1998_, and an average [TE]_sub,OWP,2010/2015_ measured in the OWP treatments in 2010 and 2015 for the final TE contents in the linear equations.*

*** Percentage of the estimated values comprised in an interval of 99%-101% compared to the data measured in 2010 and 2015:*

*- 59%, with [TE]_sub,CN,2015_ as reference for the lacking [TE]_sub,i,1998_, and the [TE]_sub,OWP,2015_ measured in the OWP treatments in 2015 for the final TE contents in the linear equations.*

*- 23%, with [TE]_sub,CN,2010_ as reference for the lacking [TE]_sub,i,1998_, and the [TE]_sub,OWP,2010_ measured in the OWP treatments in 2010 for the final TE contents in the linear equations.*

*- 23%, with an average content [TE]_sub,CN,2010/2015_ as the lacking [TE]_sub,i,1998_, and an average [TE]_sub,OWP,2010/2015_ measured in the OWP treatments, in 2010 and 2015, for the final TE contents in the linear equations.*

# Appendix E: Calculation of corrected soil stocks of trace elements

### Table E.1: Trace element soil stock of the sub-layer (Q_sub,i,n_) to be added to the trace element soil stock of the topsoil layer

With soil stock of TE of the sub-layer, Q_sub,i,n_, using estimated values for the sub-layer of the table D.3 and the soil mass differences of the table B.4.

*Co-compost of sewage sludge and green waste (GWS), biowaste compost (BIOW), compost of residual municipal solid waste (MSW), farmyard manure (FYM), no organic amendment (CN).*

|  |  | **Cd** | **Cr** | **Cu** | **Hg** | **Ni** | **Pb** | **Zn** |
| --- | --- | --- | --- | --- | --- | --- | --- | --- |
| **Treatment** | **Year** | kg ha^-1^ | kg ha^-1^ | kg ha^-1^ | kg ha^-1^ | kg ha^-1^ | kg ha^-1^ | kg ha^-1^ |
| GWS | 1998 | 0.13 | 47.8 | 8.6 | 0.04 | 10.4 | 13.5 | 32.8 |
| GWS | 2002 | 0.13 | 44.4 | 8.5 | 0.04 | 9.6 | 13.0 | 31.7 |
| GWS | 2004 | 0.12 | 40.8 | 8.0 | 0.04 | 8.7 | 12.2 | 29.7 |
| GWS | 2006 | 0.11 | 39.0 | 7.9 | 0.04 | 8.3 | 11.9 | 29.0 |
| GWS | 2007 | 0.11 | 39.1 | 8.0 | 0.04 | 8.3 | 12.1 | 29.4 |
| GWS | 2009 | 0.11 | 37.3 | 7.8 | 0.04 | 7.9 | 11.8 | 28.6 |
| GWS | 2010 | 0.09 | 31.8 | 6.9 | 0.04 | 6.7 | 10.2 | 24.8 |
| GWS | 2013 | 0.08 | 28.1 | 6.2 | 0.04 | 5.9 | 9.2 | 22.3 |
| GWS | 2015 | 0.07 | 24.5 | 5.5 | 0.03 | 5.1 | 8.1 | 19.7 |
| BIOW | 1998 | 0.13 | 47.8 | 8.6 | 0.04 | 10.4 | 13.5 | 32.8 |
| BIOW | 2002 | 0.13 | 44.0 | 8.1 | 0.04 | 9.6 | 13.2 | 31.4 |
| BIOW | 2004 | 0.12 | 40.3 | 7.5 | 0.04 | 8.8 | 12.4 | 29.3 |
| BIOW | 2006 | 0.11 | 38.4 | 7.3 | 0.04 | 8.4 | 12.2 | 28.5 |
| BIOW | 2007 | 0.11 | 38.4 | 7.3 | 0.04 | 8.4 | 12.3 | 28.7 |
| BIOW | 2009 | 0.11 | 36.5 | 7.1 | 0.04 | 8.1 | 12.0 | 27.8 |
| BIOW | 2010 | 0.10 | 31.0 | 6.1 | 0.03 | 6.9 | 10.5 | 24.1 |
| BIOW | 2013 | 0.09 | 27.3 | 5.4 | 0.03 | 6.1 | 9.5 | 21.6 |
| BIOW | 2015 | 0.08 | 23.6 | 4.7 | 0.03 | 5.2 | 8.4 | 19.0 |
| MSW | 1998 | 0.13 | 47.8 | 8.6 | 0.04 | 10.4 | 13.5 | 32.8 |
| MSW | 2002 | 0.12 | 43.8 | 8.1 | 0.04 | 9.6 | 13.1 | 31.0 |
| MSW | 2004 | 0.11 | 40.0 | 7.5 | 0.04 | 8.8 | 12.3 | 28.7 |
| MSW | 2006 | 0.11 | 38.1 | 7.3 | 0.04 | 8.3 | 12.0 | 27.8 |
| MSW | 2007 | 0.11 | 38.0 | 7.3 | 0.04 | 8.3 | 12.2 | 27.9 |
| MSW | 2009 | 0.10 | 36.1 | 7.0 | 0.04 | 7.9 | 11.9 | 26.9 |
| MSW | 2010 | 0.09 | 30.6 | 6.0 | 0.04 | 6.7 | 10.3 | 23.1 |
| MSW | 2013 | 0.08 | 26.9 | 5.4 | 0.03 | 5.9 | 9.3 | 20.6 |
| MSW | 2015 | 0.07 | 23.2 | 4.7 | 0.03 | 5.1 | 8.2 | 18.1 |
| FYM | 1998 | 0.13 | 47.8 | 8.6 | 0.04 | 10.4 | 13.5 | 32.8 |
| FYM | 2002 | 0.12 | 44.5 | 8.2 | 0.04 | 9.6 | 12.9 | 31.4 |
| FYM | 2004 | 0.12 | 40.9 | 7.6 | 0.04 | 8.8 | 12.0 | 29.3 |
| FYM | 2006 | 0.11 | 39.2 | 7.4 | 0.04 | 8.4 | 11.7 | 28.4 |
| FYM | 2007 | 0.11 | 39.2 | 7.4 | 0.04 | 8.4 | 11.8 | 28.7 |
| FYM | 2009 | 0.11 | 37.5 | 7.1 | 0.04 | 8.0 | 11.4 | 27.7 |
| FYM | 2010 | 0.09 | 32.0 | 6.2 | 0.03 | 6.8 | 9.8 | 24.0 |
| FYM | 2013 | 0.08 | 28.3 | 5.5 | 0.03 | 6.0 | 8.8 | 21.5 |
| FYM | 2015 | 0.07 | 24.6 | 4.8 | 0.03 | 5.2 | 7.8 | 18.9 |
| CN | 1998 | 0.13 | 46.0 | 8.3 | 0.04 | 10.0 | 12.9 | 31.5 |
| CN | 2002 | 0.12 | 42.3 | 7.6 | 0.04 | 9.2 | 11.9 | 29.0 |
| CN | 2004 | 0.10 | 36.8 | 6.6 | 0.03 | 8.0 | 10.4 | 25.2 |
| CN | 2006 | 0.09 | 33.1 | 6.0 | 0.03 | 7.2 | 9.3 | 22.7 |
| CN | 2007 | 0.08 | 29.4 | 5.3 | 0.03 | 6.4 | 8.3 | 20.2 |
| CN | 2009 | 0.07 | 23.9 | 4.3 | 0.02 | 5.2 | 6.7 | 16.4 |
| CN | 2010 | 0.05 | 16.6 | 3.0 | 0.01 | 3.6 | 4.7 | 11.4 |
| CN | 2013 | 0.02 | 7.4 | 1.3 | 0.01 | 1.6 | 2.1 | 5.0 |
| CN | 2015 | 0.00 | 0.0 | 0.0 | 0.00 | 0.0 | 0.0 | 0.0 |

### Table E.2: Corrected soil stock of trace elements of the topsoil layer (Qc_top,i,n_)

With corrected soil stock of TE of the topsoil layer, Qc_top,i,n_, considering the following calculations:

(1) Calculation of the “raw” soil stock of trace elements (Q_top,i,n_);

(2) Addition of the TE soil stock of the sub-layer to be incorporated to the plough layer presented in the table E.1.

*Co-compost of sewage sludge and green waste (GWS), biowaste compost (BIOW), compost of residual municipal solid waste (MSW), farmyard manure (FYM), no organic amendment (CN).*

|  | | **Cd** | **Cr** | **Cu** | **Hg** | **Ni** | **Pb** | **Zn** |
| --- | --- | --- | --- | --- | --- | --- | --- | --- |
| **Treatment** | **Year** | kg ha^-1^ | kg ha^-1^ | kg ha^-1^ | kg ha^-1^ | kg ha^-1^ | kg ha^-1^ | kg ha^-1^ |
| GWS | 1998 | 0.88 | 189.0 | 45.6 | 0.35 | 56.5 | 108.1 | 195.9 |
| GWS | 2002 | 0.90 | 178.7 | 48.3 | 0.37 | 56.9 | 90.3 | 191.4 |
| GWS | 2004 | 0.88 | 185.6 | 52.7 | 0.36 | 56.3 | 106.6 | 200.0 |
| GWS | 2006 | 0.84 | 183.8 | 55.8 | 0.35 | 58.1 | 101.6 | 207.4 |
| GWS | 2007 | 0.86 | 183.1 | 58.7 | 0.39 | 56.7 | 90.8 | 212.6 |
| GWS | 2009 | 0.89 | 253.7 | 60.1 | 0.41 | 57.3 | 97.1 | 223.2 |
| GWS | 2010 | 0.87 | 255.4 | 65.4 | 0.36 | 58.3 | 92.1 | 234.1 |
| GWS | 2013 | 0.90 | 269.2 | 70.8 | 0.41 | 59.0 | 95.4 | 243.3 |
| GWS | 2015 | 0.93 | 279.4 | 74.8 | 0.44 | 59.4 | 103.1 | 256.6 |
| BIOW | 1998 | 0.86 | 191.3 | 46.8 | 0.37 | 56.8 | 91.2 | 191.3 |
| BIOW | 2002 | 0.90 | 184.3 | 49.1 | 0.36 | 59.2 | 93.5 | 201.3 |
| BIOW | 2004 | 0.88 | 190.4 | 51.3 | 0.32 | 58.4 | 101.9 | 208.2 |
| BIOW | 2006 | 0.88 | 188.1 | 52.5 | 0.29 | 59.6 | 99.2 | 217.8 |
| BIOW | 2007 | 0.87 | 188.8 | 53.5 | 0.31 | 58.6 | 101.1 | 223.9 |
| BIOW | 2009 | 0.89 | 249.2 | 53.9 | 0.32 | 59.0 | 106.8 | 219.4 |
| BIOW | 2010 | 0.86 | 256.0 | 55.8 | 0.28 | 59.8 | 93.4 | 225.8 |
| BIOW | 2013 | 0.88 | 263.4 | 59.7 | 0.30 | 60.6 | 105.1 | 231.9 |
| BIOW | 2015 | 0.94 | 284.5 | 56.8 | 0.31 | 60.5 | 99.2 | 229.4 |
| MSW | 1998 | 0.88 | 195.3 | 45.5 | 0.35 | 56.5 | 93.0 | 200.5 |
| MSW | 2002 | 0.90 | 184.0 | 50.2 | 0.41 | 57.9 | 96.7 | 202.3 |
| MSW | 2004 | 0.89 | 188.3 | 52.9 | 0.43 | 57.1 | 105.4 | 204.7 |
| MSW | 2006 | 0.83 | 187.6 | 55.2 | 0.37 | 58.5 | 108.7 | 207.9 |
| MSW | 2007 | 0.84 | 188.5 | 55.7 | 0.42 | 58.1 | 102.8 | 211.4 |
| MSW | 2009 | 0.87 | 260.0 | 56.2 | 0.38 | 57.9 | 102.4 | 217.5 |
| MSW | 2010 | 0.83 | 247.0 | 58.8 | 0.37 | 58.0 | 93.9 | 227.9 |
| MSW | 2013 | 0.87 | 272.7 | 60.9 | 0.41 | 59.7 | 99.5 | 225.6 |
| MSW | 2015 | 0.87 | 281.3 | 60.9 | 0.37 | 59.0 | 104.4 | 230.9 |
| FYM | 1998 | 0.88 | 187.9 | 48.3 | 0.35 | 57.0 | 92.5 | 195.8 |
| FYM | 2002 | 0.93 | 188.3 | 51.5 | 0.34 | 59.3 | 95.4 | 208.3 |
| FYM | 2004 | 0.91 | 188.9 | 53.6 | 0.34 | 58.6 | 113.9 | 213.4 |
| FYM | 2006 | 0.85 | 186.9 | 54.5 | 0.30 | 58.5 | 100.6 | 212.3 |
| FYM | 2007 | 0.87 | 181.3 | 56.6 | 0.33 | 57.4 | 94.4 | 220.1 |
| FYM | 2009 | 0.93 | 256.3 | 57.2 | 0.37 | 57.8 | 116.1 | 225.8 |
| FYM | 2010 | 0.86 | 249.8 | 58.1 | 0.28 | 59.0 | 91.3 | 222.6 |
| FYM | 2013 | 0.87 | 275.2 | 60.1 | 0.30 | 59.2 | 97.1 | 228.3 |
| FYM | 2015 | 0.90 | 282.1 | 58.6 | 0.30 | 59.2 | 95.7 | 231.3 |
| CN | 1998 | 0.89 | 194.1 | 46.3 | 0.34 | 58.1 | 88.1 | 193.2 |
| CN | 2002 | 0.86 | 184.1 | 45.5 | 0.30 | 59.3 | 93.0 | 185.6 |
| CN | 2004 | 0.86 | 189.3 | 47.0 | 0.29 | 56.9 | 94.4 | 193.5 |
| CN | 2006 | 0.83 | 193.4 | 47.1 | 0.25 | 59.3 | 89.5 | 192.2 |
| CN | 2007 | 0.79 | 176.4 | 46.5 | 0.26 | 57.2 | 82.0 | 191.9 |
| CN | 2009 | 0.83 | 272.3 | 45.9 | 0.27 | 57.5 | 86.4 | 190.5 |
| CN | 2010 | 0.78 | 271.3 | 46.5 | 0.23 | 58.0 | 83.2 | 187.9 |
| CN | 2013 | 0.81 | 283.3 | 49.1 | 0.28 | 59.1 | 86.0 | 192.2 |
| CN | 2015 | 0.78 | 275.4 | 45.7 | 0.25 | 58.2 | 81.0 | 187.4 |

# Appendix F: Organic waste products characteristics

### Table F.1: Main characteristics of applied organic waste products (OWP) over the period 1998-2013

With applied quantity, dry matter (DM), pH, content of carbonates (CaCO_3_), organic carbon (Org. C), organic nitrogen (Org. N), phosphorus (P_2_O_5_) and potassium (K), the I_roc_ indicator (I_roc_); mean values ± standard deviation of the three replicates.

*Co-compost of sewage sludge and green waste (GWS), biowaste compost (BIOW), compost of residual municipal solid waste (MSW), farmyard manure (FYM).*

|  |  |  | **Applied quantity** |  | **Dry matter** | **pH** | **CaCO_3_** | **Org. C** | **Org. N** | **P (P_2_O_5_)** | **K** | **I_roc_** |
| --- | --- | --- | --- | --- | --- | --- | --- | --- | --- | --- | --- | --- |
| **OWP** | **Year** |  | t DM ha^-1^ |  | % fresh matter |  | g kg^-1^ DM | g kg^-1^ DM | g kg^-1^ DM | g kg^-1^ DM | g kg^-1^ DM |  |
| GWS | 1998 |  | 10.7 |  | 54 ± 0 | 8.5 ± 0.1 (ab) | 10 ± 1 (c) | 273 ± 11 (ab) | 26 ± 1 (a) | 19 ± 1 (a) | 18 ± 0 (ab) | 86 |
| GWS | 2000 |  | 19.8 |  | 60 ± 0 | 8.4 ± 0.1 (ab) | 10 ± 3 (c) | 191 ± 4 (bc) | 17 ± 0 (ab) | 16 ± 0 (a) | 24 ± 0 (ab) | 95 |
| GWS | 2002 |  | 18.5 |  | 53 ± 2 | 6.7 ± 0.1 (c) | 21 ± 5 (b) | 349 ± 47 (a) | 21 ± 2 (ab) | 27 ± 10 (a) | 9 ± 2 (b) | 74 ± 3 |
| GWS | 2004 |  | 17.3 |  | 63 ± 1 | 7.2 ± 0.1 (b) | 25 ± 3 (c) | 311 ± 22 (ab) | 18 ± 0 (ab) | 32 ± 3 (a) | 10 ± 1 (bc) | 65 ± 2 |
| GWS | 2006 |  | 17.7 |  | 67 ± 8 | 7.7 ± 0.1 (ab) | 38 ± 2 (b) | 245 ± 4 (bc) | 22 ± 1 (a) | 37 ± 0 (a) | 15 ± 1 (bc) | 74 ± 3 |
| GWS | 2007 |  | 15.8 |  | 59 ± 1 | 7.4 ± 0.0 (bc) | 35 ± 1 (b) | 238 ± 4 (bc) | 21 ± 0 (a) | 35 ± 0 (a) | 14 ± 0 (bc) | 78 ± 1 |
| GWS | 2009 |  | 17.2 |  | 77 ± 0 | 7.1 ± 0.1 (c) | 35 ± 3 (bc) | 256 ± 4 (b) | 21 ± 0 (ab) | 33 ± 0 (a) | 15 ± 0 (b) | 77 ± 1 |
| GWS | 2011 |  | 14.1 |  | 73 ± 0 | 6.7 ± 0.0 (c) | 32 ± 7 (bc) | 261 ± 17 (ab) | 20 ± 1 (ab) | 39 ± 3 (a) | 11 ± 0 (c) | 71 ± 2 |
| GWS | 2013 |  | 17.4 |  | 67 ± 1 | 7.7 ± 0.1 (b) | 44 ± 2 (ab) | 257 ± 3 (bc) | 19 ± 0 (a) | 28 ± 1 (a) | 17 ± 1 (bc) | 70 ± 1 |
| BIOW | 1998 |  | 16.2 |  | 77 ± 0 | 8.6 ± 0.1 (ab) | 56 ± 0 (ab) | 158 ± 13 (b) | 16 ± 1 (c) | 7 ± 0 (c) | 16 ± 0 (bc) | 77 |
| BIOW | 2000 |  | 24.5 |  | 61 ± 1 | 8.5 ± 0.2 (ab) | 89 ± 9 (a) | 176 ± 8 (c) | 12 ± 1 (b) | 9 ± 2 (ab) | 18 ± 0 (bc) | 89 |
| BIOW | 2002 |  | 25.8 |  | 69 ± 0 | 8.2 ± 0.1 (ab) | 164 ± 8 (a) | 177 ± 7 (b) | 13 ± 0 (c) | 9 ± 0 (ab) | 21 ± 0 (ab) | 75 ± 1 |
| BIOW | 2004 |  | 19.7 |  | 65 ± 1 | 8.6 ± 0.1 (ab) | 170 ± 29 (a) | 168 ± 2 (c) | 13 ± 0 (c) | 9 ± 1 (ab) | 23 ± 1 (ab) | 73 ± 1 |
| BIOW | 2006 |  | 19.4 |  | 60 ± 0 | 8.6 ± 0.0 (a) | 167 ± 2 (a) | 196 ± 10 (c) | 15 ± 0 (bc) | 10 ± 1 (bc) | 25 ± 0 (ab) | 77 ± 1 |
| BIOW | 2007 |  | 16.3 |  | 65 ± 1 | 7.6 ± 0.0 (ab) | 78 ± 4 (a) | 229 ± 5 (c) | 22 ± 0 (a) | 20 ± 1 (ab) | 19 ± 0 (ab) | 76 ± 1 |
| BIOW | 2009 |  | 18.2 |  | 83 ± 0 | 7.5 ± 0.1 (bc) | 47 ± 1 (ab) | 263 ± 14 (b) | 22 ± 1 (a) | 12 ± 0 (ab) | 23 ± 0 (ab) | 69 ± 2 |
| BIOW | 2011 |  | 12.3 |  | 81 ± 0 | 7.5 ± 0.0 (bc) | 23 ± 1 (c) | 295 ± 10 (a) | 23 ± 0 (a) | 12 ± 0 (b) | 22 ± 0 (ab) | 67 ± 2 |
| BIOW | 2013 |  | 19.7 |  | 76 ± 0 | 7.8 ± 0.1 (b) | 20 ± 1 (b) | 232 ± 5 (c) | 17 ± 0 (ab) | 9 ± 0 (b) | 23 ± 0 (ab) | 72 ± 2 |
| MSW | 1998 |  | 10.0 |  | 71 ± 0 | 8.1 ± 0.2 (b) | 71 ± 9 (a) | 304 ± 18 (a) | 19 ± 0 (bc) | 9 ± 1 (bc) | 11 ± 0 (c) | 61 |
| MSW | 2000 |  | 19.2 |  | 81 ± 1 | 7.0 ± 0.2 (b) | 53 ± 24 (ab) | 313 ± 3 (ab) | 20 ± 0 (a) | 8 ± 0 (b) | 10 ± 0 (c) | 59 |
| MSW | 2002 |  | 9.5 |  | 74 ± 0 | 7.3 ± 0.1 (bc) | 65 ± 4 (ab) | 277 ± 17 (ab) | 16 ± 1 (bc) | 6 ± 0 (b) | 9 ± 0 (b) | 48 ± 1 |
| MSW | 2004 |  | 14.6 |  | 84 ± 0 | 7.1 ± 0.0 (b) | 75 ± 1 (ab) | 263 ± 9 (bc) | 16 ± 1 (bc) | 7 ± 0 (b) | 9 ± 0 (c) | 43 ± 2 |
| MSW | 2006 |  | 10.0 |  | 56 ± 1 | 7.5 ± 0.0 (b) | 48 ± 20 (b) | 390 ± 12 (a) | 15 ± 0 (c) | 6 ± 0 (c) | 7 ± 0 (c) | 32 ± 2 |
| MSW | 2007 |  | 10.5 |  | 45 ± 0 | 7.0 ± 0.4 (c) | 61 ± 3 (ab) | 359 ± 6 (a) | 15 ± 1 (b) | 7 ± 1 (c) | 8 ± 0 (c) | 27 ± 1 |
| MSW | 2009 |  | 9.4 |  | 68 ± 0 | 7.8 ± 0.1 (ab) | 60 ± 0 (a) | 310 ± 22 (ab) | 19 ± 1 (b) | 8 ± 1 (b) | 15 ± 0 (b) | 61 ± 2 |
| MSW | 2011 |  | 13.1 |  | 62 ± 1 | 8.2 ± 0.1 (ab) | 126 ± 11 (a) | 249 ± 13 (ab) | 17 ± 0 (bc) | 12 ± 0 (b) | 16 ± 0 (bc) | 61 ± 1 |
| MSW | 2013 |  | 13.4 |  | 64 ± 0 | 7.9 ± 0.1 (ab) | 63 ± 3 (a) | 322 ± 5 (ab) | 16 ± 0 (b) | 9 ± 0 (b) | 10 ± 0 (c) | 44 ± 1 |
| FYM | 1998 |  | 13.1 |  | 24 ± 0 | 8.8 ± 0.1 (a) | 48 ± 6 (bc) | 287 ± 5 (a) | 23 ± 0 (ab) | 14 ± 1 (ab) | 34 ± 1 (a) | 71 |
| FYM | 2000 |  | 11.0 |  | 44 ± 1 | 9.0 ± 0.1 (a) | 18 ± 3 (bc) | 385 ± 33 (a) | 19 ± 1 (a) | 8 ± 1 (b) | 31 ± 1 (a) | 61 |
| FYM | 2002 |  | 15.7 |  | 38 ± 1 | 8.9 ± 0.1 (a) | 66 ± 10 (ab) | 345 ± 19 (a) | 26 ± 1 (a) | 16 ± 2 (a) | 36 ± 1 (a) | 74 ± 1 |
| FYM | 2004 |  | 13.7 |  | 37 ± 1 | 9.3 ± 0.0 (a) | 44 ± 10 (bc) | 384 ± 16 (a) | 23 ± 1 (a) | 10 ± 1 (ab) | 40 ± 1 (a) | 64 ± 1 |
| FYM | 2006 |  | 14.9 |  | 36 ± 1 | 8.8 ± 0.1 (a) | 59 ± 1 (ab) | 279 ± 19 (ab) | 22 ± 1 (ab) | 14 ± 1 (ab) | 39 ± 2 (a) | 76 ± 2 |
| FYM | 2007 |  | 13.3 |  | 36 ± 1 | 9.3 ± 0.0 (a) | 54 ± 7 (ab) | 275 ± 4 (ab) | 20 ± 1 (ab) | 14 ± 1 (bc) | 34 ± 1 (a) | 67 ± 3 |
| FYM | 2009 |  | 9.2 |  | 58 ± 1 | 9.2 ± 0.1 (a) | 25 ± 3 (c) | 407 ± 3 (a) | 22 ± 1 (a) | 13 ± 0 (ab) | 36 ± 1 (a) | 53 ± 2 |
| FYM | 2011 |  | 14.6 |  | 44 ± 1 | 9.4 ± 0.1 (a) | 64 ± 4 (ab) | 199 ± 5 (b) | 15 ± 0 (c) | 12 ± 1 (b) | 35 ± 1 (a) | 68 ± 1 |
| FYM | 2013 |  | 9.6 |  | 34 ± 1 | 8.8 ± 0.3 (a) | 46 ± 3 (ab) | 355 ± 14 (a) | 20 ± 1 (a) | 16 ± 0 (ab) | 32 ± 1 (a) | 56 ± 1 |
| **GWS** | mean 1998-2013 |  | 16.5 ± 2.7 |  | 63 ± 9 | 7.5 ± 0.6 | 28 ± 12 | 265 ± 45 | 21 ± 2 | 29 ± 8 | 15 ± 5 | 76.8 ± 9.1 |
| **BIOW** | mean 1998-2013 |  | 19.1 ± 4.2 |  | 70 ± 9 | 8.1 ± 0.5 | 90 ± 62 | 211 ± 47 | 17 ± 4 | 11 ± 4 | 21 ± 3 | 75.1 ± 6.4 |
| **MSW** | mean 1998-2013 |  | 12.2 ± 3.2 |  | 68 ± 13 | 7.5 ± 0.5 | 69 ± 23 | 310 ± 45 | 17 ± 2 | 8 ± 2 | 10 ± 3 | 48.5 ± 13.1 |
| **FYM** | mean 1998-2013 |  | 12.8 ± 2.3 |  | 40 ± 10 | 9.1 ± 0.3 | 47 ± 17 | 324 ± 68 | 21 ± 3 | 13 ± 3 | 35 ± 3 | 65.4 ± 7.9 |

Abc letters stand for significant difference between OWP treatments per year, according to Bilateral Dunn’s non-parametric tests.

### Table F.2: Trace element content in the applied OWP over the period 1998-2013

With content of Cd, Cr, Cu, Hg, Ni, Pb and Zn, expressed as mg kg^-1^ of dry matter (DM); mean values ± standard deviation of the three replicates; for data containing one or more value inferior to the quantification limit it is written into brackets < QL with the number of replicates inferior to the quantification limit.

*Co-compost of sewage sludge and green waste (GWS), biowaste compost (BIOW), compost of residual municipal solid waste (MSW), farmyard manure (FYM).*

|  |  |  |  | **Cd** | **Cr** | **Cu** | **Hg** | **Ni** | **Pb** | **Zn** |
| --- | --- | --- | --- | --- | --- | --- | --- | --- | --- | --- |
| **OWP** | **Year** |  |  | mg kg^-1^ DM | | | | | | |
| GWS | 1998 |  |  | 2.9 ± 0.2 (ab) | 30 ± 1 (a) | 154 ± 13 (ab) | 1.1 ± 0.1 (ab) | 37 ± 33 (a) | 85 ± 11 (a) | 383 ± 15 (ab) |
| GWS | 2000 |  |  | 0.7 ± 0.0 (ab) | 31 ± 2 (ab) | 86 ± 1 (ab) | 0.5 ± 0 (ab) | 14 ± 0 (ab) | 58 ± 3 (ab) | 187 ± 5 (ab) |
| GWS | 2002 |  |  | 1.0 ± 0.3 (a) | 34 ± 10 (ab) | 151 ± 39 (a) | 1.0 ± 0.4 (a) | 33 ± 9 (b) | 53 ± 21 (a) | 397 ± 126 (a) |
| GWS | 2004 |  |  | 0.9 ± 0.1 (ab) | 37 ± 3 (ab) | 167 ± 13 (b) | 0.8 ± 0.1 (b) | 34 ± 3 (b) | 52 ± 4 (a) | 415 ± 44 (b) |
| GWS | 2006 |  |  | 0.9 ± 0 (ab) | 48 ± 1 (a) | 181 ± 8 (b) | 1.0 ± 0.1 (b) | 27 ± 0 (a) | 59 ± 1 (a) | 444 ± 8 (b) |
| GWS | 2007 |  |  | 1.1 ± 0.1 (ab) | 41 ± 2 (3 < QL) | 194 ± 21 (ab) | 0.7 ± 0.0 (b) | 25 ± 1 (ab) | 71 ± 4 (ab) | 409 ± 10 (ab) |
| GWS | 2009 |  |  | 0.7 ± 0.1 | 52 ± 2 (b) | 187 ± 38 (a) | 0.5 ± 0.0 (b) | 32 ± 1 (b) | 65 ± 7 (b) | 455 ± 8 (b) |
| GWS | 2011 |  |  | 0.8 ± 0.0 | 43 ± 2 (b) | 240 ± 11 (b) | 1.3 ± 0.0 (a) | 23 ± 2 (b) | 53 ± 5 (3 < QL) | 490 ± 20 (b) |
| GWS | 2013 |  |  | 0.9 ± 0.1 | 43 ± 1 (ab) | 209 ± 12 (b) | 0.5 ± 0.0 (b) | 22 ± 0 (ab) | 66 ± 4 (1 < QL) | 471 ± 11 (ab) |
| BIOW | 1998 |  |  | 0.7 ± 0.0 (a) | 45 ± 6 (ab) | 58 ± 1 (a) | 0.5 ± 0.0 (ab) | 19 ± 3 (a) | 125 ± 8 (ab) | 211 ± 7 (a) |
| BIOW | 2000 |  |  | 2.5 ± 0.1 (b) | 67 ± 5 (ab) | 118 ± 40 (ab) | 0.3 ± 0.0 (ab) | 86 ± 2 (b) | 204 ± 25 (b) | 492 ± 14 (ab) |
| BIOW | 2002 |  |  | 0.8 ± 0.0 (a) | 44 ± 0 (ab) | 47 ± 1 (a) | 0.2 ± 0.0 (a) | 22 ± 0 (ab) | 75 ± 3 (a) | 223 ± 6 (a) |
| BIOW | 2004 |  |  | 0.3 ± 0.0 (a) | 45 ± 3 (ab) | 44 ± 2 (a) | 0.1 ± 0.0 (ab) | 26 ± 4 (ab) | 131 ± 91 (ab) | 223 ± 15 (a) |
| BIOW | 2006 |  |  | 0.4 ± 0.2 (a) | 40 ± 2 (a) | 52 ± 4 (a) | 0.1 ± 0.0 (ab) | 31 ± 19 (a) | 74 ± 19 (ab) | 231 ± 10 (a) |
| BIOW | 2007 |  |  | 0.5 ± 0.0 (a) | 28 ± 2 | 90 ± 2 (a) | 0.1 ± 0.0 (ab) | 11 ± 1 (a) | 51 ± 2 (a) | 234 ± 4 (a) |
| BIOW | 2009 |  |  | 0.3 ± 0.0 (3 < QL) | 25 ± 1 (ab) | 58 ± 4 (a) | 0.1 ± 0.0 (ab) | 10 ± 0 (ab) | 41 ± 2 (ab) | 172 ± 7 (ab) |
| BIOW | 2011 |  |  | 0.3 ± 0.0 (3 < QL) | 21 ± 2 (a) | 60 ± 3 (ab) | 0.2 ± 0.1 (a) | 9 ± 1 (a) | 39 ± 0 | 173 ± 5 (ab) |
| BIOW | 2013 |  |  | 0.3 ± 0.0 (3 < QL) | 23 ± 1 (ab) | 36 ± 1 (a) | 0.1 ± 0.0 (a) | 9 ± 1 (ab) | 44 ± 4 | 159 ± 2 (a) |
| MSW | 1998 |  |  | 2.3 ± 0.7 (ab) | 162 ± 22 (b) | 312 ± 76 (b) | 2.4 ± 0.2 (b) | 69 ± 10 (a) | 245 ± 47 (ab) | 574 ± 50 (ab) |
| MSW | 2000 |  |  | 1.7 ± 0.4 (ab) | 99 ± 16 (b) | 202 ± 2 (b) | 1.6 ± 0.0 (b) | 37 ± 10 (ab) | 172 ± 6 (ab) | 665 ± 18 (b) |
| MSW | 2002 |  |  | 1.5 ± 0.4 (a) | 167 ± 13 (b) | 168 ± 7 (a) | 1.1 ± 0.1 (a) | 29 ± 7 (ab) | 274 ± 43 (a) | 331 ± 12 (a) |
| MSW | 2004 |  |  | 1.8 ± 0.4 (b) | 129 ± 12 (b) | 95 ± 3 (ab) | 0.5 ± 0.0 (ab) | 25 ± 4 (ab) | 202 ± 22 (b) | 346 ± 11 (ab) |
| MSW | 2006 |  |  | 0.7 ± 0.1 (ab) | 43 ± 7 (a) | 104 ± 4 (ab) | 0.3 ± 0.0 (ab) | 23 ± 4 (a) | 63 ± 2 (ab) | 278 ± 4 (ab) |
| MSW | 2007 |  |  | 1.7 ± 0.7 (ab) | 50 ± 16 | 93 ± 0 (ab) | 0.3 ± 0.0 (ab) | 28 ± 10 (b) | 103 ± 16 (ab) | 280 ± 8 (ab) |
| MSW | 2009 |  |  | 0.4 ± 0.2 (2 < QL) | 23 ± 1 (ab) | 47 ± 12 (a) | 0.2 ± 0.0 (ab) | 10 ± 1 (ab) | 53 ± 7 (ab) | 158 ± 14 (a) |
| MSW | 2011 |  |  | 0.5 ± 0.0 (2 < QL) | 23 ± 1 (ab) | 56 ± 4 (ab) | 0.2 ± 0.0 (a) | 11 ± 0 (ab) | 32 ± 1 | 162 ± 2 (ab) |
| MSW | 2013 |  |  | 1.7 ± 0.1 | 77 ± 8 (b) | 208 ± 29 (ab) | 0.3 ± 0.0 (ab) | 40 ± 9 (b) | 116 ± 21 | 695 ± 35 (b) |
| FYM | 1998 |  |  | 3.6 ± 0.1 (b) | 78 ± 7 (ab) | 207 ± 4 (ab) | 0.2 ± 0.0 (a) | 35 ± 2 (a) | 404 ± 58 (b) | 716 ± 14 (b) |
| FYM | 2000 |  |  | 0.5 ± 0.0 (a) | 12 ± 3 (a) | 46 ± 4 (a) | 0.1 ± 0.0 (3 < QL) | 6 ± 1 (a) | 35 ± 4 (a) | 151 ± 10 (a) |
| FYM | 2002 |  |  | 0.7 ± 0.1 (a) | 24 ± 2 (a) | 83 ± 42 (a) | 0.1 ± 0.0 (a) | 8 ± 1 (a) | 68 ± 51 (a) | 313 ± 10 (a) |
| FYM | 2004 |  |  | 0.8 ± 0.2 (ab) | 26 ± 3 (a) | 81 ± 8 (ab) | 0.1 ± 0.0 (a) | 14 ± 2 (a) | 111 ± 25 (ab) | 372 ± 19 (ab) |
| FYM | 2006 |  |  | 1.2 ± 0.2 (b) | 48 ± 15 (a) | 101 ± 9 (ab) | 0.1 ± 0.0 (a) | 11 ± 2 (a) | 143 ± 18 (b) | 445 ± 139 (ab) |
| FYM | 2007 |  |  | 2.1 ± 0.5 (b) | 61 ± 14 | 215 ± 40 (b) | 0.1 ± 0.0 (a) | 15 ± 1 (ab) | 211 ± 11 (b) | 506 ± 43 (b) |
| FYM | 2009 |  |  | 0.3 ± 0.0 (3 < QL) | 9 ± 1 (a) | 46 ± 4 (a) | 0.0 ± 0.0 (a) | 4 ± 0 (a) | 27 ± 8 (a) | 205 ± 8 (ab) |
| FYM | 2011 |  |  | 0.3 ± 0.0 (3 < QL) | 33 ± 1 (ab) | 32 ± 1 (a) | 0.1 ± 0.0 (a) | 12 ± 0 (ab) | 22 ± 1 | 156 ± 9 (a) |
| FYM | 2013 |  |  | 0.5 ± 0.2 (2 < QL) | 14 ± 1 (a) | 44 ± 0 (ab) | 0.1 ± 0.0 (ab) | 5 ± 0 (a) | 14 ± 2 | 215 ± 3 (ab) |
| GWS | mean 1998-2013 |  |  | 1.1 ± 0.7 | 40 ± 7 | 174 ± 43 | 0.8 ± 0.3 | 27 ± 7 | 62 ± 11 | 406 ± 89 |
| BIOW | mean 1998-2013 |  |  | 0.7 ± 0.7 | 38 ± 15 | 62 ± 26 | 0.2 ± 0.1 | 25 ± 24 | 87 ± 56 | 235 ± 100 |
| MSW | mean 1998-2013 |  |  | 1.4 ± 0.7 | 86 ± 57 | 143 ± 87 | 0.8 ± 0.8 | 30 ± 18 | 140 ± 87 | 388 ± 206 |
| FYM | mean 1998-2013 |  |  | 1.1 ± 1.1 | 34 ± 24 | 95 ± 70 | 0.1 ± 0.0 | 12 ± 9 | 115 ± 127 | 342 ± 189 |

Abc letters stand for significant difference between OWP treatments per year, according to Newman-Keuls’s parametric and Bilateral Dunn’s non-parametric tests. Statistics were performed when mean values of each treatment were superior to the quantification limit (QL).

### Table F.3: Trace element input fluxes related to the applied OWP over the period 1998-2013 (Q_OWP,i,n_)

With applied quantity of OWP, input flux of organic C, Cd, Cr, Cu, Hg, Ni, Pb and Zn, expressed as kg ha^-1^.

*Co-compost of sewage sludge and green waste (GWS), biowaste compost (BIOW), compost of residual municipal solid waste (MSW), farmyard manure (FYM).*

|  |  |  | **Applied quantity** | **Applied quantity** |  | **Org. C** |  | **Cd** | **Cr** | **Cu** | **Hg** | **Ni** | **Pb** | **Zn** |
| --- | --- | --- | --- | --- | --- | --- | --- | --- | --- | --- | --- | --- | --- | --- |
| **OWP** | **Year** |  | t FM ha^-1^ | t DM ha^-1^ |  | kg ha^-1^ | | | | | | | | |
| GWS | 1998 |  | 20.0 | 10.7 |  | 2919 |  | 0.031 | 0.32 | 1.7 | 0.012 | 0.4 | 0.9 | 4.1 |
| GWS | 2000 |  | 33.0 | 19.8 |  | 3786 |  | 0.013 | 0.62 | 1.7 | 0.011 | 0.3 | 1.1 | 3.7 |
| GWS | 2002 |  | 34.8 | 18.5 |  | 6454 |  | 0.019 | 0.63 | 2.8 | 0.019 | 0.6 | 1.0 | 7.3 |
| GWS | 2004 |  | 27.4 | 17.3 |  | 5359 |  | 0.015 | 0.64 | 2.9 | 0.014 | 0.6 | 0.9 | 7.2 |
| GWS | 2006 |  | 26.5 | 17.7 |  | 4340 |  | 0.015 | 0.84 | 3.2 | 0.018 | 0.5 | 1.0 | 7.9 |
| GWS | 2007 |  | 26.7 | 15.8 |  | 3767 |  | 0.017 | 0.65 | 3.1 | 0.011 | 0.4 | 1.1 | 6.5 |
| GWS | 2009 |  | 22.3 | 17.2 |  | 4413 |  | 0.013 | 0.89 | 3.2 | 0.009 | 0.6 | 1.1 | 7.8 |
| GWS | 2011 |  | 19.3 | 14.1 |  | 3697 |  | 0.011 | 0.60 | 3.4 | 0.018 | 0.3 | 0.8 | 6.9 |
| GWS | 2013 |  | 25.9 | 17.4 |  | 4478 |  | 0.016 | 0.74 | 3.6 | 0.008 | 0.4 | 1.1 | 8.2 |
| BIOW | 1998 |  | 21.0 | 16.2 |  | 2557 |  | 0.011 | 0.73 | 0.9 | 0.009 | 0.3 | 2.0 | 3.4 |
| BIOW | 2000 |  | 40.3 | 24.5 |  | 4314 |  | 0.060 | 1.65 | 2.9 | 0.006 | 2.1 | 5.0 | 12.1 |
| BIOW | 2002 |  | 37.2 | 25.8 |  | 4586 |  | 0.021 | 1.15 | 1.2 | 0.004 | 0.6 | 1.9 | 5.8 |
| BIOW | 2004 |  | 30.3 | 19.7 |  | 3311 |  | 0.005 | 0.89 | 0.9 | 0.002 | 0.5 | 2.6 | 4.4 |
| BIOW | 2006 |  | 32.5 | 19.4 |  | 3799 |  | 0.007 | 0.77 | 1.0 | 0.003 | 0.6 | 1.4 | 4.5 |
| BIOW | 2007 |  | 25.2 | 16.3 |  | 3737 |  | 0.008 | 0.46 | 1.5 | 0.002 | 0.2 | 0.8 | 3.8 |
| BIOW | 2009 |  | 21.9 | 18.2 |  | 4782 |  | 0.005 | 0.46 | 1.1 | 0.002 | 0.2 | 0.7 | 3.1 |
| BIOW | 2011 |  | 15.2 | 12.3 |  | 3630 |  | 0.003 | 0.26 | 0.7 | 0.002 | 0.1 | 0.5 | 2.1 |
| BIOW | 2013 |  | 25.9 | 19.7 |  | 4587 |  | 0.006 | 0.45 | 0.7 | 0.002 | 0.2 | 0.9 | 3.1 |
| MSW | 1998 |  | 14.0 | 10.0 |  | 3039 |  | 0.023 | 1.62 | 3.1 | 0.024 | 0.7 | 2.5 | 5.7 |
| MSW | 2000 |  | 23.6 | 19.2 |  | 6002 |  | 0.033 | 1.89 | 3.9 | 0.031 | 0.7 | 3.3 | 12.7 |
| MSW | 2002 |  | 12.7 | 9.5 |  | 2624 |  | 0.014 | 1.58 | 1.6 | 0.011 | 0.3 | 2.6 | 3.1 |
| MSW | 2004 |  | 17.5 | 14.6 |  | 3850 |  | 0.027 | 1.89 | 1.4 | 0.008 | 0.4 | 2.9 | 5.1 |
| MSW | 2006 |  | 17.9 | 10.0 |  | 3900 |  | 0.007 | 0.43 | 1.0 | 0.003 | 0.2 | 0.6 | 2.8 |
| MSW | 2007 |  | 23.1 | 10.5 |  | 3773 |  | 0.018 | 0.53 | 1.0 | 0.003 | 0.3 | 1.1 | 2.9 |
| MSW | 2009 |  | 13.9 | 9.4 |  | 2926 |  | 0.003 | 0.22 | 0.4 | 0.002 | 0.1 | 0.5 | 1.5 |
| MSW | 2011 |  | 21.1 | 13.1 |  | 3264 |  | 0.007 | 0.30 | 0.7 | 0.002 | 0.1 | 0.4 | 2.1 |
| MSW | 2013 |  | 21.1 | 13.4 |  | 4327 |  | 0.023 | 1.04 | 2.8 | 0.005 | 0.5 | 1.6 | 9.3 |
| FYM | 1998 |  | 55.0 | 13.1 |  | 3756 |  | 0.048 | 1.02 | 2.7 | 0.002 | 0.5 | 5.3 | 9.4 |
| FYM | 2000 |  | 25.2 | 11.0 |  | 4242 |  | 0.006 | 0.13 | 0.5 | 0.001 | 0.1 | 0.4 | 1.7 |
| FYM | 2002 |  | 41.3 | 15.7 |  | 5400 |  | 0.011 | 0.00 | 1.3 | 0.002 | 0.1 | 1.1 | 4.9 |
| FYM | 2004 |  | 36.6 | 13.7 |  | 5268 |  | 0.011 | 0.35 | 1.1 | 0.001 | 0.2 | 1.5 | 5.1 |
| FYM | 2006 |  | 38.5 | 14.9 |  | 4141 |  | 0.018 | 0.71 | 1.5 | 0.001 | 0.2 | 2.1 | 6.6 |
| FYM | 2007 |  | 37.4 | 13.3 |  | 3662 |  | 0.028 | 0.82 | 2.9 | 0.001 | 0.2 | 2.8 | 6.7 |
| FYM | 2009 |  | 15.8 | 9.2 |  | 3738 |  | 0.002 | 0.09 | 0.4 | 0.000 | 0.0 | 0.2 | 1.9 |
| FYM | 2011 |  | 33.3 | 14.6 |  | 2902 |  | 0.005 | 0.48 | 0.5 | 0.002 | 0.2 | 0.3 | 2.3 |
| FYM | 2013 |  | 28.4 | 9.6 |  | 3416 |  | 0.005 | 0.13 | 0.4 | 0.001 | 0.1 | 0.1 | 2.1 |

# Appendix G: Characteristics of the organic fertilizers applied in 2014/2015

### Table G.1: Main characteristics of applied organic fertilizer in 2014 and 2015

With applied quantity, dry matter (DM), pH, content of carbonates (CaCO_3_), organic carbon (org. C), organic nitrogen (org. N), phosphorus (P_2_O_5_) and potassium (K); mean values ± standard deviation of the three replicates

*Organic waste products (OWP), no organic amendment (CN).*

| **Fertilizer application** |  |  | **Applied quantity** |  | **Dry matter** | **pH** | **CaCO_3_** | **Org. C** | **Org. N** | **P (P_2_O_5_)** | **K** |
| --- | --- | --- | --- | --- | --- | --- | --- | --- | --- | --- | --- |
| **on treatment** | **Year** |  | t DM ha^-1^ |  | % fresh matter |  | g kg^-1^ DM | g kg^-1^ DM | g kg^-1^ DM | g kg^-1^ DM | g kg^-1^ DM |
| CN | 2014 |  | 2.21 |  | 83.7 ± 0.1 | 6.4 ± 0.0 | 64.7 ± 0.8 | 410.3 ± 0.6 | 72.7 ± 0.9 | 48.3 ± 0.8 | 16.7 ± 0.2 |
| OWP + CN | 2015 |  | 0.76 |  | 85.9 ± 0.5 | 6.5 ± 0.0 | 54.3 ± 1.6 | 407.7 ± 1.5 | 69.4 ± 0.8 | 47.6 ± 0.2 | 16.6 ± 0.2 |

### Table G.2: Trace element content in the applied organic fertilizer in 2014 and 2015

With content of Cd, Cr, Cu, Hg, Ni, Pb and Zn; mean values ± standard deviation of the three replicates.

*Organic waste products (OWP), no organic amendment (CN).*

| **Fertilizer application** | |  | **Cd** | **Cr** | **Cu** | **Hg** | **Ni** | **Pb** | **Zn** |
| --- | --- | --- | --- | --- | --- | --- | --- | --- | --- |
| **on treatment** | **Year** |  | mg kg^-1^ DM | | | | | | |
| CN | 2014 |  | 0.22 ± 0.05 | 11.2 ± 0.1 | 35 ± 1.2 | 0.01 ± 0.00 | 4.5 ± 0.2 | 1.4 ± 0.3 | 264.7 ± 12.9 |
| OWP + CN | 2015 |  | 0.16 ± 0.00 | 6.9 ± 0.4 | 33.8 ± 0.6 | 0.00 ± 0.00 | 3.6 ± 0.2 | 1.7 ± 0.4 | 265.0 ± 4.6 |

### Table G.3: Trace element input fluxes related to applied organic fertilizer in 2014 and 2015

With input flux of Cd, Cr, Cu, Hg, Ni, Pb and Zn, and cumulated input flux, percentage of cumulated input flux with organic fertilizer compared to cumulated input flux with OWP (table F.3); mean values ± standard deviation of the three replicates.

*Organic waste products (OWP), no organic amendment (CN).*

| **Fertilizer application** | |  | **Cd** | **Cr** | **Cu** | **Hg** | **Ni** | **Pb** | **Zn** |
| --- | --- | --- | --- | --- | --- | --- | --- | --- | --- |
| **on treatment** | **Year** |  | g ha^-1^ | | | | | | |
| CN | 2014 |  | 0.17 ± 0.04 | 7 ± 0.6 | 26.6 ± 0.7 | 0.01 ± 0.00 | 3.4 ± 0.1 | 1.1 ± 0.2 | 201.0 ± 8.6 |
| OWP + CN | 2015 |  | 0.12 ± 0.00 | 5.2 ± 0.3 | 25.7 ± 0.5 | 0.00 ± 0.00 | 2.7 ± 0.1 | 1.3 ± 0.3 | 201.3 ± 2.3 |
| Cumulated | 2014/2015 |  | 0.29 | 12.2 | 52.3 | 0.01 | 6.1 | 2.4 | 402.4 |
| % compared to cumulated OWP input flux | 1998-2015 |  | 0.19 % | 0.21 % | 0.20 % | 0.01 % | 0.15 % | 0.03 % | 0.68 % |

# Appendix H: Amounts of trace elements in leaching waters in 2013/2015

Table H.1: Trace element contents in leaching waters

With contents of TE in the leaching waters calculated from measurements in the leaching periods 2013-2014 and 2014-2015, mean annual contents of TE (mean per year).

*Co-compost of sewage sludge and green waste (GWS), biowaste compost (BIOW), compost of residual municipal solid waste (MSW), farmyard manure (FYM), no organic amendment (CN).*

|  |  | **Cd** | **Cr** | **Cu** | **Ni** | **Pb** | **Zn** |
| --- | --- | --- | --- | --- | --- | --- | --- |
| **Treatment** | **Year** | µg L^-1^ | | | | | |
| GWS | 2013-2014 | 0.17 | 1.14 | 6.9 | 2.77 | 0.07 ^(<)^ | 17.1 |
| BIOW | 2013-2014 | 0.04 ^(<)^ | 0.97 | 5.3 | 1.90 | 0.05 ^(<)^ | 4.0 |
| MSW | 2013-2014 | 0.07 | 2.32 | 9.6 | 3.61 | 0.07 ^(<)^ | 6.0 |
| FYM | 2013-2014 | 0.04 ^(<)^ | 1.71 | 6.1 | 2.35 | 0.08 ^(<)^ | 5.7 |
| CN | 2013-2014 | 0.04 ^(<)^ | 1.57 | 3.8 | 1.72 | 0.25 ^(<)^ | 12.9 |
| GWS | 2014-2015 | 0.10 | 0.56 ^(<)^ | 8.3 | 2.86 | 0.09 | 10.6 |
| BIOW | 2014-2015 | 0.03 ^(<)^ | 0.95 | 6.7 | 2.12 | 0.06 ^(<)^ | 5.6 |
| MSW | 2014-2015 | 0.04 ^(<)^ | 1.01 | 8.7 | 3.31 | 0.07 ^(<)^ | 7.6 |
| FYM | 2014-2015 | 0.03 ^(<)^ | 1.48 | 6.9 | 2.86 | 0.47 | 8.2 |
| CN | 2014-2015 | 0.08 | 0.89 ^(<)^ | 4.2 | 1.44 | 0.12 ^(<)^ | 10.1 |
| GWS | mean per year | 0.13 | 0.85 | 7.6 | 2.82 | 0.08 | 13.8 |
| BIOW | mean per year | 0.03 | 0.96 | 6.0 | 2.01 | 0.06 | 4.8 |
| MSW | mean per year | 0.05 | 1.66 | 9.2 | 3.46 | 0.07 | 6.8 |
| FYM | mean per year | 0.04 | 1.59 | 6.5 | 2.60 | 0.28 | 7.0 |
| CN | mean per year | 0.06 | 1.23 | 4.0 | 1.58 | 0.18 | 11.5 |

^(<)^ at least half of values were inferior to the quantification limit

### Table H.2: Trace element output fluxes related to leaching waters (Q_lea,i,n_)

With amounts of TE in the leaching waters calculated from measurements in the leaching periods 2013-2014 and 2014-2015, mean annual cumulated leaching of TE (mean per year) and estimated cumulated leaching flux of TE over the period 1998-2015 (cumulated for 1998-2015).

*Co-compost of sewage sludge and green waste (GWS), biowaste compost (BIOW), compost of residual municipal solid waste (MSW), farmyard manure (FYM), no organic amendment (CN).*

|  |  | **Cd** | **Cr** | **Cu** | **Ni** | **Pb** | **Zn** |
| --- | --- | --- | --- | --- | --- | --- | --- |
| **Treatment** | **Year** | g ha^-1^ | | | | | |
| GWS | 2013-2014 | 0.30 | 1.73 | 12.9 | 5.23 | 0.12 | 30.5 |
| BIOW | 2013-2014 | 0.09 | 1.91 | 13.9 | 4.80 | 0.14 | 10.1 |
| MSW | 2013-2014 | 0.12 | 4.40 | 20.3 | 7.32 | 0.15 | 11.5 |
| FYM | 2013-2014 | 0.11 | 4.31 | 19.4 | 7.19 | 0.24 | 13.7 |
| CN | 2013-2014 | 0.10 | 2.82 | 9.2 | 4.31 | 0.57 | 33.9 |
| GWS | 2014-2015 | 0.16 | 0.94 | 12.4 | 4.11 | 0.15 | 15.1 |
| BIOW | 2014-2015 | 0.05 | 1.93 | 13.4 | 4.11 | 0.12 | 9.0 |
| MSW | 2014-2015 | 0.07 | 2.02 | 16.5 | 6.25 | 0.15 | 14.0 |
| FYM | 2014-2015 | 0.06 | 3.69 | 16.0 | 6.55 | 1.60 | 19.4 |
| CN | 2014-2015 | 0.13 | 1.49 | 7.7 | 2.59 | 0.37 | 16.8 |
| GWS | mean per year | 0.23 | 1.34 | 12.7 | 4.67 | 0.14 | 22.8 |
| BIOW | mean per year | 0.07 | 1.92 | 13.7 | 4.45 | 0.13 | 9.5 |
| MSW | mean per year | 0.09 | 3.21 | 18.4 | 6.78 | 0.15 | 12.7 |
| FYM | mean per year | 0.09 | 4.00 | 17.7 | 6.87 | 0.92 | 16.5 |
| CN | mean per year | 0.12 | 2.16 | 8.5 | 3.45 | 0.47 | 25.3 |
| GWS | cumulated for 1998-2015 | 3.88 | 22.75 | 215.6 | 79.38 | 2.30 | 387.9 |
| BIOW | cumulated for 1998-2015 | 1.15 | 32.70 | 232.5 | 75.66 | 2.19 | 162.0 |
| MSW | cumulated for 1998-2015 | 1.60 | 54.54 | 313.0 | 115.33 | 2.59 | 216.4 |
| FYM | cumulated for 1998-2015 | 1.48 | 67.99 | 301.1 | 116.86 | 15.60 | 281.2 |
| CN | cumulated for 1998-2015 | 1.98 | 36.65 | 143.8 | 58.65 | 7.98 | 430.9 |

# Appendix I: Yields and trace element contents in exported plants over the period 1998-2015

### Table I.1: Yields and trace element contents in exported wheat and barley grains over the period 1998-2015

With yields (dry matter, DM) and contents of Cd, Cr, Cu, Hg, Ni, Pb and Zn; mean values ± standard deviation of the four replicates; for data containing one or more value inferior to the quantification limit it is written into brackets < QL with the number of replicates inferior to the quantification limit.

*Co-compost of sewage sludge and green waste (GWS), biowaste compost (BIOW), compost of residual municipal solid waste (MSW), farmyard manure (FYM), no organic amendment (CN).*

| **Wheat / barley** | **Grains** |  | **Yield (DM)** |  | **Cd** | **Cr** | **Cu** | **Hg** | **Ni** | **Pb** | **Zn** |
| --- | --- | --- | --- | --- | --- | --- | --- | --- | --- | --- | --- |
|  |  |  | t DM ha^-1^ |  | mg kg^-1^ DM | mg kg^-1^ DM | mg kg^-1^ DM | mg kg^-1^ DM | mg kg^-1^ DM | mg kg^-1^ DM | mg kg^-1^ DM |
| GWS | 2000 |  | 7.4 ± 0.2 (a) |  | 0.013 ± 0.006 (2 < QL) | 0.44 ± 0.13 (a) | 2.6 ± 0.3 (a) (4 < QL) | 0.008 ± 0.009 (a) | 0.04 ± 0.08 (3 < QL) | 0.10 ± 0.00 (4 < QL) | 17 ± 1 (a) |
| BIOW | 2000 |  | 7.3 ± 0.4 (a) |  | 0.018 ± 0.010 (2 < QL) | 0.48 ± 0.3 (a) | 2.9 ± 0.2 (a) (3 < QL) | 0.071 ± 0.008 (a) | 0.13 ± 0.09 (1 < QL) | 0.10 ± 0.00 (4 < QL) | 18 ± 1 (a) |
| MSW | 2000 |  | 7.5 ± 0.4 (a) |  | 0.017 ± 0.009 (2 < QL) | 0.45 ± 0.03 (a) | 3.0 ± 0.1 (a) (3 < QL) | 0.004 ± 0.001 (a) | 0.20 ± 0.02 | 0.17 ± 0.08 (2 < QL) | 18 ± 1 (a) |
| FYM | 2000 |  | 7.8 ± 0.4 (a) |  | 0.016 ± 0.007 (2 < QL) | 0.30 ± 0.03 (a) | 2.7 ± 0.3 (a) (3 < QL) | 0.007 ± 0.004 (a) | 0.07 ± 0.06 (1 < QL) | 0.10 ± 0.00 (4 < QL) | 18 ± 1 (a) |
| CN | 2000 |  | 7.1 ± 0.3 (a) |  | 0.010 ± 0.000 (4 < QL) | 0.35 ± 0.08 (a) | 2.8 ± 0.3 (a) (3 < QL) | 0.005 ± 0.004 (a) | 0.11 ± 0.06 | 0.10 ± 0.00 (4 < QL) | 17 ± 1 (a) |
| ***Treatment effect*** | |  | ***ns*** |  |  | ***ns*** |  | ***ns*** |  |  | ***ns*** |
| GWS | 2002 |  | 7.1 ± 0.9 (a) |  | 0.016 ± 0.007 (2 < QL) | 0.21 ± 0.12 (1 < QL) | 3.0 ± 0.5 (a) | 0.009 ± 0.011 (a) | 0.12 ± 0.04 (a) | 0.10 ± 0.00 (< QL (4)) | 18 ± 1 (a) |
| BIOW | 2002 |  | 7.4 ± 0.4 (a) |  | 0.015 ± 0.006 (2 < QL) | 0.22 ± 0.08 | 3.2 ± 1 (a) | 0.010 ± 0.014 (a) | 0.14 ± 0.03 (a) | 0.10 ± 0.00 (< QL (4)) | 18 ± 1 (a) |
| MSW | 2002 |  | 7.9 ± 0.2 (a) |  | 0.018 ± 0.009 (2 < QL) | 0.23 ± 0.18 (1 < QL) | 3.1 ± 0.2 (a) | 0.014 ± 0.020 (a) | 0.14 ± 0.08 (a) | 0.10 ± 0.00 (< QL (4)) | 20 ± 1 (a) |
| FYM | 2002 |  | 7.2 ± 0.3 (a) |  | 0.018 ± 0.009 (2 < QL) | 0.26 ± 0.08 | 2.8 ± 0.1 (a) | 0.011 ± 0.014 (a) | 0.16 ± 0.08 (a) | 0.13 ± 0.06 (3 < QL) | 18 ± 0 (a) |
| CN | 2002 |  | 6.9 ± 0.6 (a) |  | 0.018 ± 0.009 (2 < QL) | 0.19 ± 0.09 (1 < QL) | 3.0 ± 0.4 (a) | 0.009 ± 0.010 (a) | 0.13 ± 0.04 (a) | 0.10 ± 0.00 (< QL 4) | 18 ± 1 (a) |
| ***Treatment effect*** | |  | ***ns*** |  |  |  | ***ns*** | ***ns*** | ***ns*** |  | ***ns*** |
| GWS | 2004 |  | 8.9 ± 0.3 (a) |  | 0.013 ± 0.005 (3 < QL) | 0.25 ± 0.09 (a) | 3.0 ± 0.1 (a) | 0.000 ± 0.000 (2 < QL) | 0.14 ± 0.05 (a) | 0.25 ± 0.03 (a) | 18 ± 1 (a) |
| BIOW | 2004 |  | 8.8 ± 0.3 (a) |  | 0.010 ± 0.000 (4 < QL) | 0.29 ± 0.08 (a) | 3.0 ± 0.3 (a) | 0.000 ± 0.000 (3 < QL) | 0.15 ± 0.04 (a) | 0.28 ± 0.05 (a) | 18 ± 1 (a) |
| MSW | 2004 |  | 8.7 ± 0.2 (a) |  | 0.010 ± 0.000 (4 < QL) | 0.39 ± 0.10 (a) | 4.0 ± 1.3 (a) | 0.000 ± 0.000 (3 < QL) | 0.20 ± 0.06 (a) | 0.33 ± 0.09 (a) | 20 ± 0 (a) |
| FYM | 2004 |  | 8.9 ± 0.3 (a) |  | 0.013 ± 0.005 (3 < QL) | 0.30 ± 0.10 (a) | 3.1 ± 0.2 (a) | 0.000 ± 0.000 (2 < QL) | 0.22 ± 0.08 (a) | 0.35 ± 0.05 (a) | 22 ± 5 (a) |
| CN | 2004 |  | 8.1 ± 0.5 (b) |  | 0.010 ± 0.000 (4 < QL) | 0.35 ± 0.11 (a) | 2.9 ± 0.0 (a) | 0.000 ± 0.000 (3 < QL) | 0.25 ± 0.07 (a) | 0.31 ± 0.03 (a) | 18 ± 2 (a) |
| ***Treatment effect*** | |  | ******* |  |  | ***ns*** | ***ns*** |  | ***ns*** | ***ns*** | ***ns*** |
| GWS | 2006 |  | 7.9 ± 0.2 (ab) |  | 0.018 ± 0.006 (a) (4 < QL) | 0.22 ± 0.12 (4 < QL) | 2.9 ± 0.3 (a) | 0.000 ± 0.000 (4 < QL) | 0.18 ± 0.15 (4 < QL) | 0.20 ± 0.14 (4 < QL) | 19 ± 1 (a) |
| BIOW | 2006 |  | 7.7 ± 0.3 (a) |  | 0.015 ± 0.006 (a) (4 < QL) | 0.18 ± 0.08 (4 < QL) | 3.2 ± 0.2 (a) | 0.000 ± 0.000 (4 < QL) | 0.06 ± 0.04 (4 < QL) | 0.20 ± 0.15 (4 < QL) | 18 ± 0 (a) |
| MSW | 2006 |  | 8.0 ± 0.3 (ab) |  | 0.017 ± 0.006 (a) (4 < QL) | 0.24 ± 0.09 (4 < QL) | 3.2 ± 0.4 (a) | 0.000 ± 0.000 (4 < QL) | 0.12 ± 0.18 (4 < QL) | 0.15 ± 0.22 (4 < QL) | 21 ± 1 (a) |
| FYM | 2006 |  | 8.1 ± 0.4 (a) |  | 0.017 ± 0.008 (a) (4 < QL) | 0.21 ± 0.13 (3 < QL) | 3.2 ± 0.2 (a) | 0.000 ± 0.000 (4 < QL) | 0.10 ± 0.04 (4 < QL) | 0.15 ± 0.1 (4 < QL) | 20 ± 1 (a) |
| CN | 2006 |  | 7.3 ± 0.4 (b) |  | 0.020 ± 0.011 (a) (4 < QL) | 0.28 ± 0.16 (3 < QL) | 3.5 ± 0.4 (a) | 0.000 ± 0.000 (4 < QL) | 0.41 ± 0.41 (3 < QL) | 0.11 ± 0.07 (4 < QL) | 20 ± 4 (a) |
| ***Treatment effect*** | |  | ******* |  |  |  | ***ns*** |  |  |  | ***ns*** |
| GWS | 2007 |  | 7.4 ± 0.6 (ab) |  | 0.002 ± 0.002 (4 < QL) | 0.39 ± 0.13 (3 < QL) | 5.5 ± 0.7 (ab) | 0.002 ± 0.001 (4 < QL) | 0.19 ± 0.16 (4 < QL) | 0.08 ± 0.10 (4 < QL) | 32 ± 1 (a) |
| BIOW | 2007 |  | 6.9 ± 0.3 (bc) |  | 0.002 ± 0.001 (4 < QL) | 0.34 ± 0.05 (4 < QL) | 5.5 ± 0.9 (ab) | 0.002 ± 0.001 (4 < QL) | 0.09 ± 0.05 (4 < QL) | 0.07 ± 0.07 (4 < QL) | 28 ± 2 (bc) |
| MSW | 2007 |  | 7.4 ± 0.1 (ab) |  | 0.005 ± 0.005 (4 < QL) | 0.37 ± 0.04 (3 < QL) | 5.8 ± 0.6 (a) | 0.002 ± 0.001 (4 < QL) | 0.11 ± 0.05 (4 < QL) | 0.07 ± 0.07 (4 < QL) | 30 ± 1 (ab) |
| FYM | 2007 |  | 7.7 ± 0.5 (a) |  | 0.004 ± 0.003 (4 < QL) | 0.37 ± 0.03 (4 < QL) | 6.3 ± 0.6 (a) | 0.001 ± 0.000 (4 < QL) | 0.13 ± 0.05 (4 < QL) | 0.1 ± 0.06 (4 < QL) | 30 ± 1 (ab) |
| CN | 2007 |  | 6.4 ± 0.6 (c) |  | 0.005 ± 0.004 (4 < QL) | 0.34 ± 0.05 (4 < QL) | 4.9 ± 0.2 (b) | 0.001 ± 0.000 (4 < QL) | 0.08 ± 0.06 (4 < QL) | 0.10 ± 0.10 (4 < QL) | 24 ± 1 (c) |
| ***Treatment effect*** | |  | ******* |  |  |  | ***ns*** |  |  |  | ******** |
| GWS | 2009 |  | 9.0 ± 0.3 (b) |  | 0.009 ± 0.006 (4 < QL) | 0.11 ± 0.08 (4 < QL) | 2.9 ± 0.3 (a) | 0.000 ± 0.000 (4 < QL) | 0.01 ± 0.01 (4 < QL) | 0.07 ± 0.05 (4 < QL) | 23 ± 1 (a) |
| BIOW | 2009 |  | 8.6 ± 0.4 (ab) |  | 0.003 ± 0.003 (4 < QL) | 0.43 ± 0.49 (3 < QL) | 3.5 ± 0.1 (ab) | 0.001 ± 0.000 (4 < QL) | 0.10 ± 0.15 (4 < QL) | 0.06 ± 0.06 (4 < QL) | 23 ± 1 (a) |
| MSW | 2009 |  | 8.8 ± 0.3 (ab) |  | 0.009 ± 0.003 (4 < QL) | 0.16 ± 0.04 (4 < QL) | 3.8 ± 0.2 (b) | 0.000 ± 0.000 (4 < QL) | 0.07 ± 0.14 (4 < QL) | 0.07 ± 0.06 (4 < QL) | 24 ± 1 (a) |
| FYM | 2009 |  | 8.9 ± 0.5 (ab) |  | 0.011 ± 0.010 (4 < QL) | 0.17 ± 0.05 (4 < QL) | 3.3 ± 0.1 (ab) | 0.000 ± 0.000 (4 < QL) | 0.02 ± 0.02 (4 < QL) | 0.07 ± 0.05 (4 < QL) | 24 ± 1 (a) |
| CN | 2009 |  | 8.1 ± 0.1 (a) |  | 0.020 ± 0.002 (4 < QL) | 0.17 ± 0.03 (4 < QL) | 3.8 ± 0.4 (b) | 0.000 ± 0.000 (4 < QL) | 0.11 ± 0.06 (4 < QL) | 0.09 ± 0.04 (4 < QL) | 22 ± 1 (a) |
| ***Treatment effect*** | |  | ******* |  |  |  | ******** |  |  |  | ***ns*** |
| GWS | 2011 |  | 7.9 ± 0.3 (a) |  | 0.018 ± 0.002 (c) | 0.27 ± 0.18 (a) | 2.4 ± 0.3 (a) | 0.001 ± 0.001 (4 < QL) | 0.22 ± 0.05 (bc) | 0.09 ± 0.05 (1 < QL) | 17 ± 1 (b) |
| BIOW | 2011 |  | 8.1 ± 0.2 (a) |  | 0.021 ± 0.002 (bc) | 0.19 ± 0.03 (a) | 2.9 ± 0.1 (ab) | 0.000 ± 0.000 (4 < QL) | 0.18 ± 0.03 (c) | 0.07 ± 0.03 (2 < QL) | 20 ± 1 (a) |
| MSW | 2011 |  | 7.8 ± 0.5 (a) |  | 0.023 ± 0.002 (b) | 0.10 ± 0.01 (a) | 3.2 ± 0.3 (b) | 0.001 ± 0.001 (4 < QL) | 0.16 ± 0.01 (c) | 0.21 ± 0.15 (1 < QL) | 20 ± 1 (a) |
| FYM | 2011 |  | 8.2 ± 0.4 (a) |  | 0.023 ± 0.002 (b) | 0.21 ± 0.07 (a) | 3.1 ± 0.4 (ab) | 0.000 ± 0.000 (4 < QL) | 0.24 ± 0.03 (b) | 0.20 ± 0.14 (1 < QL) | 20 ± 1 (a) |
| CN | 2011 |  | 7.0 ± 0.6 (b) |  | 0.029 ± 0.002 (a) | 0.19 ± 0.07 (a) | 3 ± 0.1 (ab) | 0.000 ± 0.000 (4 < QL) | 0.31 ± 0.04 (a) | 0.18 ± 0.09 | 18 ± 1 (a) |
| ***Treatment effect*** | |  | ******** |  | ******** | ***ns*** | ******* |  | ******** |  | ******** |
| GWS | 2013 |  | 8.0 ± 0.1 (a) |  | 0.017 ± 0.002 (c) | 0.02 ± 0.03 (3 < QL) | 3.9 ± 0.3 (a) | 0.000 ± 0.000 (4 < QL) | 0.08 ± 0.02 | 0.04 ± 0.01 (3 < QL) | 28 ± 1 (a) |
| BIOW | 2013 |  | 7.5 ± 0.3 (b) |  | 0.016 ± 0.002 (c) | 0.00 ± 0.01 (4 < QL) | 4 ± 0.1 (a) | 0.001 ± 0.001 (4 < QL) | 0.05 ± 0.01 (1 < QL) | 0.05 ± 0.02 (1 < QL) | 27 ± 2 (a) |
| MSW | 2013 |  | 6.9 ± 0.3 (c) |  | 0.020 ± 0.003 (c) | 0.04 ± 0.07 (4 < QL) | 4.1 ± 0.4 (a) | 0.001 ± 0.000 (4 < QL) | 0.12 ± 0.07 | 0.11 ± 0.13 (1 < QL) | 28 ± 1 (a) |
| FYM | 2013 |  | 7.3 ± 0.4 (b) |  | 0.025 ± 0.005 (b) | 0.01 ± 0.01 (3 < QL) | 4.2 ± 0.2 (a) | 0.001 ± 0.001 (4 < QL) | 0.07 ± 0.02 | 0.05 ± 0.01 (1 < QL) | 28 ± 1 (a) |
| CN | 2013 |  | 6.2 ± 0.2 (d) |  | 0.036 ± 0.002 (a) | 0.01 ± 0.01 (3 < QL) | 4.1 ± 0.1 (a) | 0.001 ± 0.000 (4 < QL) | 0.19 ± 0.01 | 0.05 ± 0.02 (1 < QL) | 27 ± 1 (a) |
| ***Treatment effect*** | |  | ******** |  | ******** |  | ***ns*** |  |  |  | ***ns*** |
| GWS | 2015 |  | 6.9 ± 0.7 (a) |  | 0.005 ± 0.001 (4 < QL) | 0.00 ± 0.00 (4 < QL) | 3.9 ± 0.1 (a) | 0.000 ± 0.000 (4 < QL) | 0.01 ± 0.01 (4 < QL) | 0.03 ± 0.01 (4 < QL) | 28 ± 1 (b) |
| BIOW | 2015 |  | 6.5 ± 0.3 (a) |  | 0.005 ± 0.002 (4 < QL) | 0.00 ± 0.00 (4 < QL) | 4.1 ± 0.3 (a) | 0.000 ± 0.000 (4 < QL) | 0.03 ± 0.03 (4 < QL) | 0.04 ± 0 (4 < QL) | 28 ± 1 (b) |
| MSW | 2015 |  | 5.8 ± 0.7 (ab) |  | 0.007 ± 0.001 (4 < QL) | 0.00 ± 0.00 (4 < QL) | 4.1 ± 0.1 (a) | 0.000 ± 0.000 (4 < QL) | 0.04 ± 0.03 (4 < QL) | 0.05 ± 0.02 (4 < QL) | 29 ± 1 (a) |
| FYM | 2015 |  | 5.9 ± 0.7 (ab) |  | 0.007 ± 0.002 (4 < QL) | 0.00 ± 0.00 (4 < QL) | 4.0 ± 0.1 (a) | 0.000 ± 0.000 (4 < QL) | 0.05 ± 0.07 (3 < QL) | 0.06 ± 0.02 (4 < QL) | 27 ± 1 (bc) |
| CN | 2015 |  | 5.0 ± 0.1 (b) |  | 0.009 ± 0.003 (2 < QL) | 0.03 ± 0.04 (2 < QL) | 4.0 ± 0.1 (a) | 0.000 ± 0.000 (4 < QL) | 0.07 ± 0.04 (3 < QL) | 0.41 ± 0.68 (2 < QL) | 27 ± 0 (c) |
| ***Treatment effect*** | |  | ******* |  |  |  | ***ns*** |  |  |  | ******** |

ANOVA and Kruskal-Wallis tests were performed to test the treatment effect: ns stand for no significant difference while * and ** indicate significant difference at the probability level of *P<0.05* and *P<0.01* respectively. Abc letters stand for significant difference between treatments per year, according to Newman-Keuls’s parametric and Bilateral Dunn’s non-parametric tests.

Statistics were performed when mean values of each treatment were superior to the quantification limit (QL).

### Table I.2: Yields and trace element contents in exported wheat and barley residues over the period 1998-2015

With yields (dry matter, DM) and contents of Cd, Cr, Cu, Hg, Ni, Pb and Zn; mean values ± standard deviation of the four replicates; for data containing one or more value inferior to the quantification limit it is written into brackets < QL with the number of replicates inferior to the quantification limit.

*Co-compost of sewage sludge and green waste (GWS), biowaste compost (BIOW), compost of residual municipal solid waste (MSW), farmyard manure (FYM), no organic amendment (CN).*

| **Wheat / barley** | **Residues** |  | **Yield (DM)** |  | **Cd** | **Cr** | **Cu** | **Hg** | **Ni** | **Pb** | **Zn** |
| --- | --- | --- | --- | --- | --- | --- | --- | --- | --- | --- | --- |
|  |  |  | t DM ha^-1^ |  | mg kg^-1^ DM | mg kg^-1^ DM | mg kg^-1^ DM | mg kg^-1^ DM | mg kg^-1^ DM | mg kg^-1^ DM | mg kg^-1^ DM |
| GWS | 2000 |  | 5.38 ± 0.10 (b) |  | 0.055 ± 0.005 (b) | 2.0 ± 0.3 (a) | 2.2 ± 0.6 (4 < QL) | 0.082 ± 0.030 (a) | 0.71 ± 0.17 (a) | 0.96 ± 0.24 (a) | 8.4 ± 2.3 (a) |
| BIOW | 2000 |  | 5.21 ± 0.15 (b) |  | 0.060 ± 0.005 (ab) | 2.1 ± 0.6 (a) | 2.2 ± 0.7 (3 < QL) | 0.090 ± 0.022 (a) | 0.96 ± 0.35 (a) | 1.10 ± 0.07 (a) | 7.8 ± 1.6 (a) |
| MSW | 2000 |  | 5.36 ± 0.51 (b) |  | 0.066 ± 0.008 (a) | 1.7 ± 0.2 (a) | 2.3 ± 0.6 (4 < QL) | 0.071 ± 0.035 (a) | 0.68 ± 0.09 (a) | 0.95 ± 0.17 (a) | 7.5 ± 1.1 (a) |
| FYM | 2000 |  | 5.76 ± 0.16 (a) |  | 0.006 ± 0.006 (ab) | 2.3 ± 0.5 (a) | 2.3 ± 0.6 (3 < QL) | 0.061 ± 0.013 (a) | 0.88 ± 0.28 (a) | 0.97 ± 0.13 (a) | 6.9 ± 1.3 (a) |
| CN | 2000 |  | 53.00 ± 0.12 (b) |  | 0.065 ± 0.007 (a) | 1.9 ± 0.2 (a) | 1.9 ± 0.1 (4 < QL) | 0.076 ± 0.015 (a) | 0.77 ± 0.19 (a) | 0.86 ± 0.12 (a) | 7.1 ± 0.8 (a) |
| ***Treatment effect*** | |  | ******* |  | ***ns*** | ***ns*** |  | ***ns*** | ***ns*** | ***ns*** | ***ns*** |
| GWS | 2002 |  | 4.23 ± 0.49 (ab) |  | 0.047 ± 0.006 (a) | 0.4 ± 0.0 (b) | 2.4 ± 0.7 (a) | 0.109 ± 0.031 (a) | 0.23 ± 0.08 (a) | 0.27 ± 0.13 (1 < QL) | 6.5 ± 0.5 (a) |
| BIOW | 2002 |  | 4.42 ± 0.15 (ab) |  | 0.043 ± 0.008 (a) | 0.5 ± 0.1 (ab) | 2.1 ± 0.4 (a) | 0.116 ± 0.037 (a) | 0.28 ± 0.1 (a) | 0.34 ± 0.06 | 5.5 ± 0.6 (a) |
| MSW | 2002 |  | 4.75 ± 0.22 (a) |  | 0.049 ± 0.008 (a) | 0.7 ± 0.2 (a) | 2.9 ± 0.4 (a) | 0.140 ± 0.101 (a) | 0.33 ± 0.1 (a) | 0.34 ± 0.16 (1 < QL) | 6.6 ± 0.8 (a) |
| FYM | 2002 |  | 4.38 ± 0.24 (ab) |  | 0.044 ± 0.007 (a) | 0.6 ± 0.2 (ab) | 2.6 ± 0.7 (a) | 0.174 ± 0.063 (a) | 0.35 ± 0.1 (a) | 0.30 ± 0.17 (1 < QL) | 6.1 ± 0.8 (a) |
| CN | 2002 |  | 3.99 ± 0.32 (b) |  | 0.052 ± 0.012 (a) | 0.7 ± 0.1 (a) | 1.7 ± 0.4 (a) | 0.132 ± 0.039 (a) | 0.31 ± 0.05 (a) | 0.28 ± 0.13 (1 < QL) | 5.7 ± 0.6 (a) |
| ***Treatment effect*** | |  | ******* |  | ***ns*** | ******* | ***ns*** | ***ns*** | ***ns*** |  | ***ns*** |
| GWS | 2004 |  | 6.34 ± 0.21 (a) |  | 0.054 ± 0.005 (a) | 0.5 ± 0.2 (a) | 2 ± 0.2 (a) | 0.015 ± 0.005 (a) | 0.17 ± 0.08 (a) | 0.27 ± 0.02 | 6.8 ± 1.4 (a) |
| BIOW | 2004 |  | 6.25 ± 0.23 (b) |  | 0.043 ± 0.002 (a) | 0.5 ± 0.0 (a) | 1.6 ± 0.1 (a) | 0.013 ± 0.003 (a) | 0.15 ± 0.02 (a) | 0.18 ± 0.09 (2 < QL) | 6.7 ± 0.6 (b) |
| MSW | 2004 |  | 6.25 ± 0.2(a) |  | 0.052 ± 0.006 (a) | 0.6 ± 0.1 (a) | 1.9 ± 0.1 (a) | 0.012 ± 0.001 (a) | 0.18 ± 0.04 (a) | 0.21 ± 0.07 (1 < QL) | 7 ± 1.3 (a) |
| FYM | 2004 |  | 6.53 ± 0.34 (b) |  | 0.055 ± 0.010 (a) | 0.5 ± 0.1 (a) | 2.0 ± 0.8 (a) | 0.014 ± 0.005 (a) | 0.19 ± 0.11 (a) | 0.3 ± 0.23 (1 < QL) | 6.9 ± 1 (b) |
| CN | 2004 |  | 5.77 ± 0.35 (b) |  | 0.050 ± 0.007 (a) | 0.6 ± 0.1 (a) | 2.1 ± 0.7 (a) | 0.011 ± 0.004 (a) | 0.15 ± 0.04 (a) | 0.2 ± 0.15 (2 < QL) | 5.4 ± 0.9 (b) |
| ***Treatment effect*** | |  | ******** |  | ***ns*** | ***ns*** | ***ns*** | ***ns*** | ***ns*** |  | ***ns*** |
| GWS | 2006 |  | 5.53 ± 0.33 (a) |  | 0.049 ± 0.013 (4 < QL) | 0.4 ± 0.1 (3 < QL) | 2.1 ± 0.2 (2 < QL) | 0.019 ± 0.002 (a) | 0.16 ± 0.13 (4 < QL) | 0.24 ± 0.11 (4 < QL) | 4.1 ± 1 (a) |
| BIOW | 2006 |  | 5.37 ± 0.26 (a) |  | 0.033 ± 0.005 (4 < QL) | 0.5 ± 0.1 | 1.9 ± 0.4 (3 < QL) | 0.016 ± 0.004 (a) | 0.38 ± 0.19 (2 < QL) | 0.33 ± 0.07 (4 < QL) | 3.5 ± 0.4 (a) |
| MSW | 2006 |  | 5.67 ± 0.29 (a) |  | 0.042 ± 0.006 (4 < QL) | 0.6 ± 0.2 (1 < QL) | 2.1 ± 0.4 (2 < QL) | 0.015 ± 0.001 (a) | 0.45 ± 0.13 (1 < QL) | 0.27 ± 0.04 (4 < QL) | 4.2 ± 0.7 (a) |
| FYM | 2006 |  | 5.43 ± 0.22 (a) |  | 0.015 ± 0.003 (4 < QL) | 1.0 ± 0.1 (2 < QL) | 2.8 ± 0.2 (3 < QL) | 0.011 ± 0.001 (a) | 0.72 ± 0.19 (2 < QL) | 0.19 ± 0.07 (4 < QL) | 9.1 ± 1.2 (a) |
| CN | 2006 |  | 5.09 ± 0.29 (a) |  | 0.049 ± 0.012 (4 < QL) | 0.5 ± 0.1 (1 < QL) | 1.8 ± 0.2 (4 < QL) | 0.016 ± 0.004 (a) | 0.19 ± 0.15 (3 < QL) | 0.27 ± 0.06 (4 < QL) | 3.4 ± 0.8 (a) |
| ***Treatment effect*** | |  | ***ns*** |  |  |  |  | ***ns*** |  |  | ***ns*** |
| GWS | 2007 |  | 6.09 ± 0.19 (a) |  | 0.019 ± 0.000 (4 < QL) | 0.7 ± 0.1 (b) | 3.1 ± 0.2 (a) | 0.013 ± 0.002 | 0.41 ± 0.12 (2 < QL) | 0.27 ± 0.02 (4 < QL) | 10 ± 1.3 (a) |
| BIOW | 2007 |  | 4.77 ± 0.30 (bc) |  | 0.044 ± 0.066 (4 < QL) | 0.8 ± 0.2 (ab) | 2.8 ± 0.6 (ab) | 0.009 ± 0.005 (1 < QL) | 0.48 ± 0.18 (2 < QL) | 0.18 ± 0.05 (4 < QL) | 7.2 ± 0.8 (bc) |
| MSW | 2007 |  | 4.94 ± 0.07 (bc) |  | 0.016 ± 0.003 (4 < QL) | 1 ± 0.3 (ab) | 2.9 ± 0.2 (ab) | 0.012 ± 0.000 | 0.60 ± 0.21 (1 < QL) | 0.23 ± 0.03 (4 < QL) | 10.1 ± 1.2 (a) |
| FYM | 2007 |  | 5.87 ± 0.37 (ab) |  | 0.043 ± 0.001 (4 < QL) | 0.5 ± 0.2 (a) | 2.0 ± 0.1 (ab) | 0.018 ± 0.001 (1 < QL) | 0.31 ± 0.38 (1 < QL) | 0.22 ± 0.04 (4 < QL) | 4.3 ± 1.1 (ab) |
| CN | 2007 |  | 3.84 ± 0.38 (c) |  | 0.017 ± 0.008 (4 < QL) | 1.0 ± 0.1 (ab) | 2.6 ± 0.2 (b) | 0.010 ± 0.001 (2 < QL) | 0.56 ± 0.14 (2 < QL) | 0.17 ± 0.03 (4 < QL) | 5.1 ± 0.9 (c) |
| ***Treatment effect*** | |  | ******** |  |  | ***ns*** | ***ns*** |  |  |  | ******** |
| GWS | 2009 |  | 5.66 ± 0.14 (ab) |  | 0.043 ± 0.003 | 1.0 ± 0.8 (2 < QL) | 2.1 ± 0.2 (a) | 0.014 ± 0.001 (ab) | 0.66 ± 0.75 (2 < QL) | 0.13 ± 0.02 (4 < QL) | 4.7 ± 0.6 (a) |
| BIOW | 2009 |  | 5.48 ± 0.19 (ab) |  | 0.037 ± 0.007 (1 < QL) | 0.9 ± 0.9 (1 < QL) | 2.2 ± 0.2 (a) | 0.014 ± 0.001 (b) | 0.47 ± 0.78 (3 < QL) | 0.17 ± 0.05 (3 < QL) | 5.1 ± 0.6 (a) |
| MSW | 2009 |  | 5.60 ± 0.35 (ab) |  | 0.052 ± 0.007 | 0.7 ± 0.6 (2 < QL) | 2.2 ± 0.1 (a) | 0.014 ± 0.002 (ab) | 1.56 ± 2.37 (2 < QL) | 0.15 ± 0.02 (4 < QL) | 4.7 ± 0.4 (a) |
| FYM | 2009 |  | 5.84 ± 0.20 (b) |  | 0.046 ± 0.008 | 1.5 ± 0.8 | 2.3 ± 0.1 (a) | 0.013 ± 0.001 (ab) | 1.06 ± 0.68 (1 < QL) | 0.19 ± 0.02 (3 < QL) | 5.2 ± 0.2 (a) |
| CN | 2009 |  | 5.02 ± 0.34 (a) |  | 0.074 ± 0.011 | 1.1 ± 0.7 | 2.0 ± 0.2 (a) | 0.011 ± 0.001 (a) | 0.61 ± 0.62 (2 < QL) | 0.13 ± 0.03 (4 < QL) | 4.2 ± 0.6 (a) |
| ***Treatment effect*** | |  | ******* |  |  |  | ***ns*** | ******* |  |  | ***ns*** |
| GWS | 2011 |  | 5.52 ± 0.31 (a) |  | 0.046 ± 0.007 (b) | 0.4 ± 0.1 (a) | 1.8 ± 0.1 (a) | 0.011 ± 0.003 (2 < QL) | 0.33 ± 0.15 (a) | 0.29 ± 0.11 (a) | 3.2 ± 0.5 (b) |
| BIOW | 2011 |  | 5.46 ± 0.17 (a) |  | 0.043 ± 0.008 (b) | 0.3 ± 0.1 (a) | 2.2 ± 0.7 (a) | 0.011 ± 0.001 | 0.27 ± 0.06 (a) | 0.25 ± 0.12 (a) | 4.3 ± 0.5 (a) |
| MSW | 2011 |  | 5.25 ± 0.41 (a) |  | 0.050 ± 0.004 (b) | 0.4 ± 0.1 (a) | 1.9 ± 0.2 (a) | 0.010 ± 0.002 (1 < QL) | 0.30 ± 0.08 (a) | 0.27 ± 0.13 (a) | 4.3 ± 0.7 (a) |
| FYM | 2011 |  | 5.84 ± 0.15 (a) |  | 0.046 ± 0.007 (b) | 0.4 ± 0.1 (a) | 1.9 ± 0.2 (a) | 0.012 ± 0.002 | 0.33 ± 0.15 (a) | 0.22 ± 0.04 (a) | 4.3 ± 0.5 (a) |
| CN | 2011 |  | 4.54 ± 0.25 (b) |  | 0.061 ± 0.002 (a) | 0.4 ± 0.1 (a) | 1.7 ± 0.1 (a) | 0.012 ± 0.002 | 0.28 ± 0.07 (a) | 0.30 ± 0.15 (a) | 4.0 ± 0.8 (a) |
| ***Treatment effect*** | |  | ******* |  | ******** | ***ns*** | ***ns*** |  | ***ns*** | ***ns*** | ******* |
| GWS | 2013 |  | 6.55 ± 0.12 (b) |  | 0.039 ± 0.006 (a) | 0.4 ± 0.1 (ab) | 2.3 ± 0.2 (ab) | 0.016 ± 0.002 (a) | 0.24 ± 0.05 (a) | 0.18 ± 0.03 (a) | 6.5 ± 0.7 (a) |
| BIOW | 2013 |  | 5.75 ± 0.27 (ab) |  | 0.039 ± 0.002 (a) | 0.4 ± 0.1 (ab) | 2.6 ± 0.3 (a) | 0.017 ± 0.003 (a) | 0.19 ± 0.04 (a) | 0.18 ± 0.01 (a) | 6.5 ± 1 (a) |
| MSW | 2013 |  | 5.27 ± 0.54 (ab) |  | 0.056 ± 0.004 (ab) | 0.6 ± 0.1 (a) | 2.6 ± 0.2 (a) | 0.017 ± 0.002 (a) | 0.23 ± 0.04 (a) | 0.19 ± 0.04 (a) | 6.7 ± 1.1 (a) |
| FYM | 2013 |  | 5.88 ± 0.28 (ab) |  | 0.047 ± 0.005 (ab) | 0.4 ± 0.1 (b) | 2.2 ± 0.1 (b) | 0.014 ± 0.002 (a) | 0.20 ± 0.04 (a) | 0.13 ± 0.02 (a) | 6 ± 0.3 (a) |
| CN | 2013 |  | 4.56 ± 0.12 (a) |  | 0.111 ± 0.009 (b) | 0.5 ± 0.1 (a) | 2.1 ± 0.1 (b) | 0.013 ± 0.002 (a) | 0.21 ± 0.04 (a) | 0.14 ± 0.02 (a) | 6.3 ± 0.8 (a) |
| ***Treatment effect*** | |  | ******* |  | ******** | ******* | ******** | ******* | ***ns*** | ******* | ***ns*** |
| GWS | 2015 |  | 3.54 ± 0.33 (a) |  | 0.015 ± 0.008 (2 < QL) | 0.7 ± 0.1 (a) | 2.7 ± 0.5 (ab) | 0.011 ± 0.001 | 0.59 ± 0.14 (a) | 0.13 ± 0.04 (1 < QL) | 8.4 ± 0.4 (ab) |
| BIOW | 2015 |  | 3.48 ± 0.16 (a) |  | 0.012 ± 0.003 (2 < QL) | 0.9 ± 0.2 (ab) | 2.2 ± 0.1 (b) | 0.010 ± 0.001 | 0.6 ± 0.11 (a) | 0.12 ± 0.03 | 7.1 ± 0.5 (c) |
| MSW | 2015 |  | 2.82 ± 0.47 (ab) |  | 0.017 ± 0.003 | 1.1 ± 0.3 (ab) | 2.3 ± 0.4 (ab) | 0.009 ± 0.001 | 0.75 ± 0.19 (a) | 0.21 ± 0.12 | 8.8 ± 0.3 (a) |
| FYM | 2015 |  | 2.93 ± 0.56 (ab) |  | 0.015 ± 0.002 | 1.1 ± 0.1 (ab) | 2.2 ± 0.3 (b) | 0.009 ± 0.001 (3 < QL) | 0.80 ± 0.11 (a) | 0.11 ± 0.03 (1 < QL) | 7.6 ± 0.8 (bc) |
| CN | 2015 |  | 2.13 ± 0.12 (b) |  | 0.018 ± 0.006 | 1.4 ± 0.4 (b) | 2.8 ± 0.4 (a) | 0.009 ± 0.001 (2 < QL) | 0.72 ± 0.12 (a) | 0.13 ± 0.04 (1 < QL) | 7.0 ± 0.2 (c) |
| ***Treatment effect*** | |  | ******* |  |  | ******* | ******* |  | ***ns*** |  | ******** |

ANOVA and Kruskal-Wallis tests were performed to test the treatment effect: ns stand for no significant difference while * and ** indicate significant difference at the probability level of *P<0.05* and *P<0.01* respectively. Abc letters stand for significant difference between treatments per year, according to Newman-Keuls’s parametric and Bilateral Dunn’s non-parametric tests.

Statistics were performed when mean values of each treatment were superior to the quantification limit (QL).

### Table I.3: Yields and trace element contents in exported maize grains over the period 1998-2015

With yields (dry matter, DM) and contents of Cd, Cr, Cu, Hg, Ni, Pb and Zn; mean values ± standard deviation of the four replicates; for data containing one or more value inferior to the quantification limit it is written into brackets < QL with the number of replicates inferior to the quantification limit.

*Co-compost of sewage sludge and green waste (GWS), biowaste compost (BIOW), compost of residual municipal solid waste (MSW), farmyard manure (FYM), no organic amendment (CN).*

| **Maize** | **Grains** |  | **Yield (DM)** |  | **Cd** | **Cr** | **Cu** | **Hg** | **Ni** | **Pb** | **Zn** |
| --- | --- | --- | --- | --- | --- | --- | --- | --- | --- | --- | --- |
|  |  |  | t DM ha^-1^ |  | mg kg^-1^ DM | mg kg^-1^ DM | mg kg^-1^ DM | mg kg^-1^ DM | mg kg^-1^ DM | mg kg^-1^ DM | mg kg^-1^ DM |
| GWS | 1999 |  | 10.3 ± 0.3 (b) |  | 0.005 ± 0.000 (4 < QL) | 0.05 ± 0.03 (2 < QL) | 2.8 ± 0.4 (a) | 0.001 ± 0.000 (a) | 0.87 ± 0.97 (a) | 0.05 ± 0.00 (4 < QL) | 14.3 ± 1.5 (a) |
| BIOW | 1999 |  | 10.2 ± 0.3 (b) |  | 0.005 ± 0.000 (4 < QL) | 0.05 ± 0.05 (3 < QL) | 2.8 ± 0.4 (a) | 0.001 ± 0.000 (a) | 0.31 ± 0.09 (a) | 0.05 ± 0.00 (4 < QL) | 14.6 ± 1.2 (a) |
| MSW | 1999 |  | 10.4 ± 0.3 (b) |  | 0.007 ± 0.003 (3 < QL) | 0.21 ± 0.38 (3 < QL) | 2.8 ± 0.3 (a) | 0.001 ± 0.000 (a) | 0.42 ± 0.10 (a) | 0.05 ± 0.00 (4 < QL) | 14.9 ± 2.4 (a) |
| FYM | 1999 |  | 10.9 ± 0.3 (a) |  | 0.005 ± 0.000 (4 < QL) | 0.06 ± 0.07 (3 < QL) | 2.8 ± 0.5 (a) | 0.001 ± 0.000 (a) | 0.33 ± 0.07 (a) | 0.09 ± 0.08 (34 < QL) | 14.4 ± 1.0 (a) |
| CN | 1999 |  | 10.4 ± 0.2 (b) |  | 0.007 ± 0.004 (3 < QL) | 0.04 ± 0.03 (3 < QL) | 2.9 ± 0.2 (a) | 0.001 ± 0.000 (a) | 0.33 ± 0.07 (a) | 0.05 ± 0.00 (4 < QL) | 13.9 ± 0.7 (a) |
| ***Treatment effect*** | |  | ******** |  |  |  | ***ns*** | ***ns*** | ***ns*** |  | ***ns*** |
| GWS | 2001 |  | 9.3 ± 0.4 (b) |  | 0.010 ± 0 (4 < QL) | 0.19 ± 0.05 (a) | 1.9 ± 0.4 (4 < QL) | 0.000 ± 0.000 (a) | 0.22 ± 0.09 (a) | 0.10 ± 0.00 (4 < QL) | 19.6 ± 1.9 (a) |
| BIOW | 2001 |  | 9.3 ± 0.4 (b) |  | 0.010 ± 0 (4 < QL) | 0.16 ± 0.03 (a) | 2 ± 0.2 (4 < QL) | 0.000 ± 0.000 (a) | 0.20 ± 0.03 (a) | 0.15 ± 0.10 (3 < QL) | 18.7 ± 1.2 (a) |
| MSW | 2001 |  | 9.9 ± 0.1 (a) |  | 0.015 ± 0.01 (3 < QL) | 0.18 ± 0.02 (a) | 2.1 ± 0.3 (4 < QL) | 0.001 ± 0.002 (a) | 0.19 ± 0.02 (a) | 0.10 ± 0.00 (4 < QL) | 19.8 ± 0.5 (a) |
| FYM | 2001 |  | 9.2 ± 0.5 (b) |  | 0.010 ± 0 (4 < QL) | 0.16 ± 0.02 (a) | 1.8 ± 0.6 (4 < QL) | 0.000 ± 0.000 (a) | 0.23 ± 0.04 (a) | 0.13 ± 0.06 (3 < QL) | 19.9 ± 1.9 (a) |
| CN | 2001 |  | 9.0 ± 0.5 (b) |  | 0.010 ± 0 (4 < QL) | 0.21 ± 0.08 (a) | 1.9 ± 0.1 (4 < QL) | 0.000 ± 0.000 (a) | 0.28 ± 0.10 (a) | 0.15 ± 0.09 (3 < QL) | 19.2 ± 1.9 (a) |
| ***Treatment effect*** | |  | ******* |  |  | ***ns*** |  | ***ns*** | ***ns*** |  | ***ns*** |
| GWS | 2003 |  | 7.8 ± 0.3 (a) |  | 0.010 ± 0 (4 < QL) | 0.05 ± 0.00 (4 < QL) | 2.4 ± 0.3 (a) | 0.000 ± 0.000 (2 < QL) | 0.36 ± 0.07 (a) | 0.10 ± 0.00 (4 < QL) | 19.3 ± 1.3 (a) |
| BIOW | 2003 |  | 8.1 ± 0.3 (a) |  | 0.016 ± 0.01 (3 < QL) | 0.05 ± 0.00 (4 < QL) | 2.5 ± 0.3 (a) | 0.000 ± 0.000 (4 < QL) | 0.34 ± 0.05 (a) | 0.10 ± 0.00 (4 < QL) | 19.9 ± 2 (a) |
| MSW | 2003 |  | 8.1 ± 0.7 (a) |  | 0.010 ± 0 (4 < QL) | 0.05 ± 0.00 (4 < QL) | 2.5 ± 0.1 (a) | 0.000 ± 0.000 (3 < QL) | 0.31 ± 0.07 (a) | 0.13 ± 0.06 (3 < QL) | 20.6 ± 1.4 (a) |
| FYM | 2003 |  | 8.3 ± 0.4 (a) |  | 0.010 ± 0 (4 < QL) | 0.05 ± 0.00 (4 < QL) | 2.5 ± 0.2 (a) | 0.000 ± 0.000 (3 < QL) | 0.37 ± 0.08 (a) | 0.10 ± 0.00 (4 < QL) | 22.1 ± 2.1 (a) |
| CN | 2003 |  | 8.3 ± 0.4 (a) |  | 0.010 ± 0 (4 < QL) | 0.05 ± 0.00 (4 < QL) | 2.4 ± 0.1 (a) | 0.000 ± 0.000 (3 < QL) | 0.40 ± 0.10 (a) | 0.10 ± 0.00 (4 < QL) | 18.8 ± 1.3 (a) |
| ***Treatment effect*** | |  | ***ns*** |  |  |  | ***ns*** |  | ***ns*** |  | ***ns*** |
| GWS | 2005 |  | 8.9 ± 0.3 (a) |  | 0.001 ± 0.002 (4 < QL) | 0.13 ± 0.05 (1 < QL) | 1.9 ± 0.2 (3 < QL) | 0.000 ± 0.000 (4 < QL) | 0.33 ± 0.11 (a) | 0.10 ± 0.07 (4 < QL) | 15.6 ± 0.6 (b) |
| BIOW | 2005 |  | 8.8 ± 0.4 (a) |  | 0.002 ± 0.003 (4 < QL) | 0.16 ± 0.02 | 2.3 ± 0.4 (1 < QL) | 0.000 ± 0.000 (4 < QL) | 0.32 ± 0.02 (a) | 0.12 ± 0.11 (3 < QL) | 18.4 ± 1.1 (a) |
| MSW | 2005 |  | 8.7 ± 0.5 (a) |  | 0.005 ± 0.006 (4 < QL) | 0.14 ± 0.03 | 2.0 ± 0.2 (2 < QL) | 0.000 ± 0.000 (4 < QL) | 0.30 ± 0.01 (a) | 0.09 ± 0.10 (4 < QL) | 17.0 ± 1.5 (ab) |
| FYM | 2005 |  | 9.1 ± 0.3 (a) |  | 0.003 ± 0.005 (4 < QL) | 0.14 ± 0.06 (1 < QL) | 2.0 ± 0.2 (3 < QL) | 0.000 ± 0.000 (4 < QL) | 0.29 ± 0.04 (a) | 0.05 ± 0.06 (4 < QL) | 16.7 ± 1.0 (ab) |
| CN | 2005 |  | 7.9 ± 0.2 (a) |  | 0.001 ± 0.002 (4 < QL) | 0.14 ± 0.02 | 2.0 ± 0.2 (2 < QL) | 0.000 ± 0.000 (4 < QL) | 0.35 ± 0.05 (a) | 0.07 ± 0.06 (4 < QL) | 17.4 ± 1.1 (ab) |
| ***Treatment effect*** | |  | ***ns*** |  |  |  | ***ns*** |  | ***ns*** |  | ******* |
| GWS | 2008 |  | 8.9 ± 0.5 (ab) |  | 0.001 ± 0.001 (4 < QL) | 0.00 ± 0.00 (4 < QL) | 2.3 ± 0.2 (a) | 0.000 ± 0.000 (4 < QL) | 0.2 ± 0.03 (4 < QL) | 0.00 ± 0.00 (4 < QL) | 15.9 ± 0.7 (a) |
| BIOW | 2008 |  | 8.9 ± 0.6 (ab) |  | 0.000 ± 0.000 (4 < QL) | 0.01 ± 0.02 (4 < QL) | 2.6 ± 0.1 (ab) | 0.000 ± 0.000 (4 < QL) | 0.18 ± 0.03 (4 < QL) | 0.00 ± 0.00 (4 < QL) | 17.5 ± 1.2 (a) |
| MSW | 2008 |  | 8.7 ± 0.4 (ab) |  | 0.001 ± 0.011 (4 < QL) | 0.00 ± 0.00 (4 < QL) | 2.7 ± 0.1 (b) | 0.000 ± 0.000 (4 < QL) | 0.19 ± 0.04 (4 < QL) | 0.00 ± 0.00 (4 < QL) | 18.8 ± 1.4 (a) |
| FYM | 2008 |  | 9.2 ± 0.4 (a) |  | 0.002 ± 0.004 (4 < QL) | 0.02 ± 0.01 (4 < QL) | 2.5 ± 0.1 (ab) | 0.000 ± 0.000 (4 < QL) | 0.19 ± 0.01 (4 < QL) | 0.00 ± 0.00 (4 < QL) | 17.1 ± 0.7 (a) |
| CN | 2008 |  | 8.3 ± 0.4 (b) |  | 0.000 ± 0.000 (4 < QL) | 0.00 ± 0.01 (4 < QL) | 2.6 ± 0.1 (ab) | 0.000 ± 0.000 (4 < QL) | 0.22 ± 0.02 (4 < QL) | 0.01 ± 0.01 (4 < QL) | 17.4 ± 1.0 (a) |
| ***Treatment effect*** | |  | ***ns*** |  |  |  | ***ns*** |  |  |  | ***ns*** |
| GWS | 2010 |  | 9.3 ± 0.3 (a) |  | 0.015 ± 0.00 (4 < QL) | 0.06 ± 0.02 (4 < QL) | 2.3 ± 0.2 (a) | 0.000 ± 0.000 (4 < QL) | 0.17 ± 0.10 (4 < QL) | 0.00 ± 0.00 (4 < QL) | 17.3 ± 1.1 (b) |
| BIOW | 2010 |  | 9.4 ± 0.5 (a) |  | 0.015 ± 0.00 (4 < QL) | 0.07 ± 0.05 (4 < QL) | 2.3 ± 0.1 (a) | 0.000 ± 0.000 (4 < QL) | 0.09 ± 0.04 (4 < QL) | 0.00 ± 0.00 (4 < QL) | 20.6 ± 0.6 (a) |
| MSW | 2010 |  | 9.5 ± 0.5 (a) |  | 0.023 ± 0.016 (3 < QL) | 0.09 ± 0.04 (4 < QL) | 2.6 ± 0.3 (a) | 0.000 ± 0.000 (4 < QL) | 0.13 ± 0.05 (4 < QL) | 0.02 ± 0.04 (4 < QL) | 20.4 ± 1.2 (a) |
| FYM | 2010 |  | 9.7 ± 0.4 (a) |  | 0.015 ± 0.00 (4 < QL) | 0.05 ± 0.02 (4 < QL) | 2.5 ± 0.2 (a) | 0.000 ± 0.000 (4 < QL) | 0.10 ± 0.02 (4 < QL) | 0.02 ± 0.03 (4 < QL) | 19.5 ± 1.2 (a) |
| CN | 2010 |  | 9.0 ± 0.4 (a) |  | 0.015 ± 0.00 (4 < QL) | 0.04 ± 0.02 (4 < QL) | 2.5 ± 0.1 (a) | 0.000 ± 0.000 (4 < QL) | 0.13 ± 0.01 (4 < QL) | 0.11 ± 0.14 (4 < QL) | 19.3 ± 1.4 (a) |
| ***Treatment effect*** | |  | ***ns*** |  |  |  | ***ns*** |  |  |  | ******** |
| GWS | 2012 |  | 9.5 ± 0.3 (ab) |  | 0.000 ± 0.000 (4 < QL) | 0.12 ± 0.02 (a) | 1.3 ± 0.1 (b) | 0.000 ± 0.000 (4 < QL) | 0.09 ± 0.02 (2 < QL) | 0.01 ± 0.01 (4 < QL) | 19.4 ± 0.9 (ab) |
| BIOW | 2012 |  | 9.6 ± 0.5 (ab) |  | 0.000 ± 0.000 (4 < QL) | 0.11 ± 0.02 (a) | 1.7 ± 0.2 (a) | 0.000 ± 0.000 (4 < QL) | 0.09 ± 0.03 (2 < QL) | 0.01 ± 0.02 (4 < QL) | 21.6 ± 1.3 (ab) |
| MSW | 2012 |  | 9.0 ± 0.5 (ab) |  | 0.000 ± 0.000 (4 < QL) | 0.18 ± 0.05 (a) | 1.5 ± 0.1 (a) | 0.000 ± 0.000 (4 < QL) | 0.14 ± 0.06 (2 < QL) | 0.04 ± 0.02 (4 < QL) | 23.0 ± 3.3 (a) |
| FYM | 2012 |  | 10.1 ± 0.3 (b) |  | 0.000 ± 0.000 (4 < QL) | 0.14 ± 0.03 (a) | 1.5 ± 0.1 (a) | 0.000 ± 0.000 (4 < QL) | 0.14 ± 0.06 (1 < QL) | 0.01 ± 0.01 (4 < QL) | 18.7 ± 0.6 (b) |
| CN | 2012 |  | 8.3 ± 0.3 (a) |  | 0.002 ± 0.00 (4 < QL) | 0.19 ± 0.05 (a) | 1.5 ± 0.1 (a) | 0.000 ± 0.000 (4 < QL) | 0.2 ± 0.05 | 0.00 ± 0.00 (4 < QL) | 21.0 ± 1.3 (ab) |
| ***Treatment effect*** | |  | ******* |  |  | ***ns*** | ******** |  |  |  | ******* |
| GWS | 2014 |  | 8.4 ± 0.7 (a) |  | 0.001 ± 0.001 (4 < QL) | 0.00 ± 0.01 (4 < QL) | 1.5 ± 0.1 (a) | 0.000 ± 0.000 (4 < QL) | 0.04 ± 0.02 (2 < QL) | 0.08 ± 0.09 (1 < QL) | 21.0 ± 1.4 (c) |
| BIOW | 2014 |  | 8.6 ± 1.0 (a) |  | 0.000 ± 0.001 (4 < QL) | 0.03 ± 0.03 (1 < QL) | 1.7 ± 0.5 (a) | 0.000 ± 0.000 (4 < QL) | 0.06 ± 0.03 (1 < QL) | 0.06 ± 0.08 (3 < QL) | 22.3 ± 1.0 (bc) |
| MSW | 2014 |  | 7.3 ± 1.5 (a) |  | 0.001 ± 0.001 (4 < QL) | 0.03 ± 0.04 (2 < QL) | 1.6 ± 0.4 (a) | 0.000 ± 0.000 (4 < QL) | 0.09 ± 0.02 | 0.05 ± 0.03 (2 < QL) | 25.1 ± 1.4 (ab) |
| FYM | 2014 |  | 7.8 ± 0.3 (ab) |  | 0.001 ± 0.000 (4 < QL) | 0.01 ± 0.02 (3 < QL) | 1.5 ± 0.2 (a) | 0.000 ± 0.000 (4 < QL) | 0.08 ± 0.02 | 0.07 ± 0.03 (1 < QL) | 25.2 ± 4.1 (ab) |
| CN | 2014 |  | 3.9 ± 0.7 (b) |  | 0.001 ± 0.002 (4 < QL) | 0.04 ± 0.04 (1 < QL) | 1.5 ± 0.2 (a) | 0.000 ± 0.000 (4 < QL) | 0.18 ± 0.04 | 0.31 ± 0.29 (1 < QL) | 26.1 ± 1.2 (a) |
| ***Treatment effect*** | |  | ******* |  |  |  | ***ns*** |  |  |  | ******* |

ANOVA and Kruskal-Wallis tests were performed to test the treatment effect: ns stand for no significant difference while * and ** indicate significant difference at the probability level of *P<0.05* and *P<0.01* respectively. Abc letters stand for significant difference between treatments per year, according to Newman-Keuls’s parametric and Bilateral Dunn’s non-parametric tests.

Statistics were performed when mean values of each treatment were superior to the quantification limit (QL).

# Appendix J: Mean annual input and output measured in the QualiAgro device 1998-2015

### Table: Mean annual input (Q_m_ input) and output (Q_m_ output) fluxes measured in the QualiAgro device 1998-2015, compared to fluxes cited in the literature

With mean input fluxes stand for atmospheric deposits and OWP input (in italics for spreading amounts of OWP corrected to represent the extreme local scenario), output fluxes stand for water drainage and plant exportations. The output fluxes of Cd, Cr, Hg, Ni and Pb are only estimations, since most of the trace element contents were lower than the quantification limits in grains.

*Co-compost of sewage sludge and green waste (GWS), biowaste compost (BIOW), compost of residual municipal solid waste (MSW), farmyard manure (FYM), no organic amendment (CN).*

|  | **Mean annual flux (1998-2015)** | **Cd** | **Cr** | **Cu** | **Hg** | **Ni** | **Pb** | **Zn** |
| --- | --- | --- | --- | --- | --- | --- | --- | --- |
|  |  | g ha^-1^ year^-1^ | | | | | | |
| **GWS** | Input OWP flux | 8.8 | 348.8 | 1505.9 | 7.1 | 235.3 | 535.3 | 3505.9 |
|  | *Corrected input OWP flux* | *5.9* | *232.5* | *1003.9* | *4.7* | *156.9* | *356.9* | *2337.3* |
|  | Plant exportations | 0.2 | 2.6 | 26.9 | 0.1 | 2.4 | 1.3 | 179.5 |
|  | Drainage outputs | 0.2 | 1.3 | 12.7 | na | 4.7 | 0.1 | 22.8 |
| **BIOW** | Input OWP flux | 7.6 | 401.8 | 641.2 | 1.8 | 282.4 | 935.3 | 2488.2 |
|  | *Corrected input OWP flux* | *5.1* | *267.8* | *427.5* | *1.2* | *188.2* | *623.5* | *1658.8* |
|  | Plant exportations | 0.2 | 2.7 | 28.1 | 0.2 | 1.9 | 1.3 | 181.5 |
|  | Drainage outputs | 0.1 | 1.9 | 13.7 | na | 4.5 | 0.1 | 9.5 |
| **MSW** | Input OWP flux | 8.8 | 558.8 | 941.2 | 5.3 | 200.0 | 911.8 | 2664.7 |
|  | *Corrected input OWP flux* | *5.9* | *372.5* | *627.5* | *3.5* | *133.3* | *607.8* | 1776.5 |
|  | Plant exportations | 0.1 | 1.4 | 23.1 | 0.0 | 1.2 | 0.8 | 154.4 |
|  | Drainage outputs | 0.1 | 3.2 | 18.4 | na | 6.8 | 0.2 | 12.7 |
| **FYM** | Input OWP flux | 7.6 | 241.8 | 664.7 | 0.6 | 88.2 | 817.6 | 2388.2 |
|  | *Corrected input OWP flux* | *5.1* | *161.2* | *443.1* | *0.4* | *58.8* | *545.1* | *1592.2* |
|  | Plant exportations | 0.1 | 1.3 | 23.2 | 0.0 | 1.2 | 0.8 | 148.7 |
|  | Drainage outputs | 0.1 | 4.0 | 17.7 | na | 6.9 | 0.9 | 16.5 |
| **CN** | Plant exportations | 0.1 | 1.0 | 16.7 | 0.0 | 1.2 | 0.7 | 106.9 |
|  | Drainage outputs | 0.1 | 2.2 | 8.5 | na | 3.4 | 0.5 | 25.3 |
| Belon et al. 2012 | Mean annual atmospheric inputs in France | 0.03 | 2.39 | 0.30 | 3.00 | 0.01 | 0.50 | 0.20 |
|  | TE maximum inputs in France | 6.1 | 146.2 | 696.5 | 1.5 | 66.8 | 205.4 | 2433.0 |
|  | TE average inputs in France | 1.8 | 33.9 | 164.7 | 0.4 | 19.2 | 23.5 | 514.0 |
|  | TE maximum inputs flows in French standard* | 15.0 | 600.0 | 1000.0 | 10.0 | 300.0 | 900.0 | 3000.0 |

** NFU 44-051 and NFU 44-095*

*na: not available*

# Appendix K: Simulation model calculations and accuracy

### Tables: Simulations of trace element contents in soils for the period 1998-2015, using data of Appendix J and the equation (8)

[TE]_top,n_ = [TE]_top, n – 1_ + $\frac{Qm input-Qm output}{Wtop}$ (8)

Tables contain:

- simulated data calculated using the actual QualiAgro input fluxes of OWP,
- a percentage of deviation between simulated data and data measured *in situ* in 2007 for Cr (change of the analytical method after 2007) and 2015 for Cd, Cu, Hg, Ni, Pb and Zn (data in Appendix C), to evaluate the accuracy of the simulation model to estimate data.

*Co-compost of sewage sludge and green waste (GWS), biowaste compost (BIOW), compost of residual municipal solid waste (MSW), farmyard manure (FYM), no organic amendment (CN).*

|  | **Cd** | | | | | **Cr** | | | | |
| --- | --- | --- | --- | --- | --- | --- | --- | --- | --- | --- |
|  | mg kg^-1^ DM | | | | | mg kg^-1^ DM | | | | |
|  | **GWS** | **BIOW** | **MSW** | **FYM** | **CN** | **GWS** | **BIOW** | **MSW** | **FYM** | **CN** |
| 1998 | 0.24 | 0.24 | 0.24 | 0.24 | 0.24 | 45.66 | 45.66 | 45.66 | 45.66 | 45.66 |
| 1999 | 0.24 | 0.24 | 0.24 | 0.24 | 0.24 | 45.75 | 45.76 | 45.81 | 45.72 | 45.66 |
| 2000 | 0.24 | 0.24 | 0.24 | 0.24 | 0.24 | 45.84 | 45.87 | 45.95 | 45.78 | 45.66 |
| 2001 | 0.24 | 0.24 | 0.24 | 0.24 | 0.24 | 45.93 | 45.97 | 46.10 | 45.85 | 45.66 |
| 2002 | 0.25 | 0.25 | 0.25 | 0.25 | 0.24 | 46.02 | 46.08 | 46.24 | 45.91 | 45.66 |
| 2003 | 0.25 | 0.25 | 0.25 | 0.25 | 0.24 | 46.11 | 46.18 | 46.39 | 45.97 | 45.66 |
| 2004 | 0.25 | 0.25 | 0.25 | 0.25 | 0.24 | 46.20 | 46.29 | 46.54 | 46.03 | 45.65 |
| 2005 | 0.25 | 0.25 | 0.25 | 0.25 | 0.24 | 46.30 | 46.39 | 46.68 | 46.10 | 45.65 |
| 2006 | 0.26 | 0.25 | 0.26 | 0.25 | 0.24 | 46.39 | 46.50 | 46.83 | 46.16 | 45.65 |
| **2007** | 0.26 | 0.26 | 0.26 | 0.26 | 0.24 | **46.48** | **46.60** | **46.97** | **46.22** | **45.65** |
| 2008 | 0.26 | 0.26 | 0.26 | 0.26 | 0.24 | 46.57 | 46.71 | 47.12 | 46.28 | 45.65 |
| 2009 | 0.26 | 0.26 | 0.26 | 0.26 | 0.24 | 46.66 | 46.81 | 47.26 | 46.34 | 45.65 |
| 2010 | 0.26 | 0.26 | 0.27 | 0.26 | 0.24 | 46.75 | 46.91 | 47.41 | 46.41 | 45.65 |
| 2011 | 0.27 | 0.26 | 0.27 | 0.26 | 0.24 | 46.84 | 47.02 | 47.56 | 46.47 | 45.65 |
| 2012 | 0.27 | 0.27 | 0.27 | 0.27 | 0.24 | 46.93 | 47.12 | 47.70 | 46.53 | 45.65 |
| 2013 | 0.27 | 0.27 | 0.27 | 0.27 | 0.24 | 47.02 | 47.23 | 47.85 | 46.59 | 45.65 |
| 2014 | 0.27 | 0.27 | 0.27 | 0.27 | 0.24 | 47.11 | 47.33 | 47.99 | 46.66 | 45.65 |
| **2015** | **0.28** | **0.27** | **0.28** | **0.27** | **0.24** | 47.20 | 47.44 | 48.14 | 46.72 | 45.65 |
| **% deviation 2015** | ***11.9%*** | ***9.4%*** | ***19.3%*** | ***14.1%*** | ***16.3%*** | ***-35.7%*** | ***-36.8%*** | ***-35.2%*** | ***-36.9%*** | ***-37.0%*** |
| **% deviation 2007** |  |  |  |  |  | ***5.7%*** | ***1.5%*** | ***2.3%*** | ***6.6%*** | ***5.6%*** |

|  | **Cu** | | | | | **Hg** | | | | |
| --- | --- | --- | --- | --- | --- | --- | --- | --- | --- | --- |
|  | mg kg^-1^ DM | | | | | mg kg^-1^ DM | | | | |
|  | **GWS** | **BIOW** | **MSW** | **FYM** | **CN** | **GWS** | **BIOW** | **MSW** | **FYM** | **CN** |
| 1998 | 12.03 | 12.03 | 12.03 | 12.03 | 12.03 | 0.26 | 0.26 | 0.26 | 0.26 | 0.26 |
| 1999 | 12.42 | 12.19 | 12.27 | 12.19 | 12.02 | 0.26 | 0.26 | 0.26 | 0.26 | 0.26 |
| 2000 | 12.80 | 12.35 | 12.50 | 12.36 | 12.02 | 0.26 | 0.26 | 0.26 | 0.26 | 0.26 |
| 2001 | 13.19 | 12.50 | 12.74 | 12.52 | 12.01 | 0.27 | 0.26 | 0.26 | 0.26 | 0.26 |
| 2002 | 13.57 | 12.66 | 12.98 | 12.69 | 12.00 | 0.27 | 0.26 | 0.27 | 0.26 | 0.26 |
| 2003 | 13.96 | 12.82 | 13.21 | 12.85 | 12.00 | 0.27 | 0.26 | 0.27 | 0.26 | 0.26 |
| 2004 | 14.35 | 12.98 | 13.45 | 13.02 | 11.99 | 0.27 | 0.26 | 0.27 | 0.26 | 0.26 |
| 2005 | 14.73 | 13.13 | 13.69 | 13.18 | 11.98 | 0.27 | 0.26 | 0.27 | 0.26 | 0.26 |
| 2006 | 15.12 | 13.29 | 13.92 | 13.34 | 11.98 | 0.27 | 0.26 | 0.27 | 0.26 | 0.26 |
| 2007 | 15.50 | 13.45 | 14.16 | 13.51 | 11.97 | 0.28 | 0.26 | 0.27 | 0.26 | 0.26 |
| 2008 | 15.89 | 13.61 | 14.40 | 13.67 | 11.96 | 0.28 | 0.26 | 0.27 | 0.26 | 0.26 |
| 2009 | 16.27 | 13.77 | 14.63 | 13.84 | 11.96 | 0.28 | 0.26 | 0.28 | 0.26 | 0.26 |
| 2010 | 16.66 | 13.92 | 14.87 | 14.00 | 11.95 | 0.28 | 0.26 | 0.28 | 0.26 | 0.26 |
| 2011 | 17.05 | 14.08 | 15.11 | 14.16 | 11.94 | 0.28 | 0.27 | 0.28 | 0.26 | 0.26 |
| 2012 | 17.43 | 14.24 | 15.34 | 14.33 | 11.94 | 0.29 | 0.27 | 0.28 | 0.26 | 0.26 |
| 2013 | 17.82 | 14.40 | 15.58 | 14.49 | 11.93 | 0.29 | 0.27 | 0.28 | 0.26 | 0.26 |
| 2014 | 18.20 | 14.55 | 15.82 | 14.66 | 11.92 | 0.29 | 0.27 | 0.28 | 0.26 | 0.26 |
| **2015** | **18.59** | **14.71** | **16.06** | **14.82** | **11.92** | **0.29** | **0.27** | **0.28** | **0.26** | **0.26** |
| **% deviation 2015** | ***-6.7%*** | ***-1.8%*** | ***-0.7%*** | ***-4.2%*** | ***-0.9%*** | ***146.7%*** | ***221.6%*** | ***189.3%*** | ***228.1%*** | ***299.9%*** |

|  | **Ni** | | | | | **Pb** | | | | |
| --- | --- | --- | --- | --- | --- | --- | --- | --- | --- | --- |
|  | mg kg^-1^ DM | | | | | mg kg^-1^ DM | | | | |
|  | **GWS** | **BIOW** | **MSW** | **FYM** | **CN** | **GWS** | **BIOW** | **MSW** | **FYM** | **CN** |
| 1998 | 14.76 | 14.76 | 14.76 | 14.76 | 14.76 | 25.74 | 25.74 | 25.74 | 25.74 | 25.74 |
| 1999 | 14.82 | 14.83 | 14.81 | 14.78 | 14.76 | 25.88 | 25.99 | 25.98 | 25.95 | 25.74 |
| 2000 | 14.88 | 14.91 | 14.86 | 14.80 | 14.76 | 26.02 | 26.23 | 26.22 | 26.17 | 25.74 |
| 2001 | 14.94 | 14.98 | 14.91 | 14.82 | 14.76 | 26.16 | 26.48 | 26.46 | 26.38 | 25.74 |
| 2002 | 15.00 | 15.05 | 14.96 | 14.84 | 14.76 | 26.30 | 26.72 | 26.70 | 26.60 | 25.74 |
| 2003 | 15.06 | 15.12 | 15.01 | 14.87 | 14.75 | 26.44 | 26.97 | 26.94 | 26.81 | 25.74 |
| 2004 | 15.12 | 15.20 | 15.06 | 14.89 | 14.75 | 26.58 | 27.21 | 27.18 | 27.03 | 25.74 |
| 2005 | 15.18 | 15.27 | 15.11 | 14.91 | 14.75 | 26.72 | 27.46 | 27.42 | 27.24 | 25.74 |
| 2006 | 15.24 | 15.34 | 15.16 | 14.93 | 14.75 | 26.86 | 27.71 | 27.66 | 27.46 | 25.74 |
| 2007 | 15.30 | 15.41 | 15.21 | 14.95 | 14.75 | 27.00 | 27.95 | 27.90 | 27.67 | 25.74 |
| 2008 | 15.36 | 15.49 | 15.27 | 14.97 | 14.75 | 27.14 | 28.20 | 28.14 | 27.89 | 25.74 |
| 2009 | 15.42 | 15.56 | 15.32 | 14.99 | 14.75 | 27.29 | 28.44 | 28.38 | 28.10 | 25.74 |
| 2010 | 15.48 | 15.63 | 15.37 | 15.01 | 14.75 | 27.43 | 28.69 | 28.62 | 28.32 | 25.74 |
| 2011 | 15.54 | 15.70 | 15.42 | 15.03 | 14.74 | 27.57 | 28.93 | 28.86 | 28.53 | 25.74 |
| 2012 | 15.60 | 15.78 | 15.47 | 15.06 | 14.74 | 27.71 | 29.18 | 29.10 | 28.75 | 25.74 |
| 2013 | 15.66 | 15.85 | 15.52 | 15.08 | 14.74 | 27.85 | 29.43 | 29.34 | 28.96 | 25.74 |
| 2014 | 15.72 | 15.92 | 15.57 | 15.10 | 14.74 | 27.99 | 29.67 | 29.58 | 29.18 | 25.74 |
| **2015** | **15.78** | **15.99** | **15.62** | **15.12** | **14.74** | **28.13** | **29.92** | **29.81** | **29.39** | **25.73** |
| **% deviation 2015** | ***0.9%*** | ***0.6%*** | ***0.7%*** | ***-2.8%*** | ***-3.8%*** | ***2.9%*** | ***14.6%*** | ***7.7%*** | ***16.1%*** | ***20.7%*** |

|  | **Zn** | | | | |
| --- | --- | --- | --- | --- | --- |
|  | mg kg^-1^ DM | | | | |
|  | **GWS** | **BIOW** | **MSW** | **FYM** | **CN** |
| 1998 | 51.60 | 51.60 | 51.60 | 51.60 | 51.60 |
| 1999 | 52.46 | 52.20 | 52.25 | 52.18 | 51.56 |
| 2000 | 53.33 | 52.80 | 52.91 | 52.77 | 51.53 |
| 2001 | 54.20 | 53.41 | 53.57 | 53.35 | 51.49 |
| 2002 | 55.07 | 54.01 | 54.22 | 53.94 | 51.46 |
| 2003 | 55.94 | 54.62 | 54.88 | 54.52 | 51.42 |
| 2004 | 56.81 | 55.22 | 55.54 | 55.11 | 51.39 |
| 2005 | 57.68 | 55.83 | 56.20 | 55.69 | 51.35 |
| 2006 | 58.55 | 56.43 | 56.85 | 56.28 | 51.32 |
| 2007 | 59.42 | 57.04 | 57.51 | 56.86 | 51.28 |
| 2008 | 60.29 | 57.64 | 58.17 | 57.45 | 51.25 |
| 2009 | 61.16 | 58.25 | 58.83 | 58.03 | 51.21 |
| 2010 | 62.03 | 58.85 | 59.48 | 58.62 | 51.18 |
| 2011 | 62.90 | 59.45 | 60.14 | 59.20 | 51.14 |
| 2012 | 63.77 | 60.06 | 60.80 | 59.79 | 51.11 |
| 2013 | 64.64 | 60.66 | 61.45 | 60.37 | 51.07 |
| 2014 | 65.51 | 61.27 | 62.11 | 60.96 | 51.04 |
| **2015** | **66.38** | **61.87** | **62.77** | **61.54** | **51.00** |
| **% deviation 2015** | ***-2.6%*** | ***2.2%*** | ***2.5%*** | ***0.7%*** | ***3.4%*** |
